# Heme Interactions as Regulators of the Alternative Pathway Complement Responses and Implications for Heme-Associated Pathologies

**DOI:** 10.3390/cimb45060330

**Published:** 2023-06-16

**Authors:** Stefanos A. Tsiftsoglou

**Affiliations:** Laboratory of Pharmacology, School of Pharmacy, Faculty of Health Sciences, Aristotle University of Thessaloniki, 54124 Thessaloniki, Greece; satsiftsoglou@pharm.auth.gr or stefanos.tsiftsoglou@gmail.com

**Keywords:** ferroptosis, heme, HeBPs, HBMs, complement, alternative pathway, genetic, genomic, SNPs, epigenetic, PTMs, hemostasis, pathology, ClinVar

## Abstract

Heme (Fe^2+^-protoporphyrin IX) is a pigment of life, and as a prosthetic group in several hemoproteins, it contributes to diverse critical cellular processes. While its intracellular levels are tightly regulated by networks of heme-binding proteins (HeBPs), labile heme can be hazardous through oxidative processes. In blood plasma, heme is scavenged by hemopexin (HPX), albumin and several other proteins, while it also interacts directly with complement components C1q, C3 and factor I. These direct interactions block the classical pathway (CP) and distort the alternative pathway (AP). Errors or flaws in heme metabolism, causing uncontrolled intracellular oxidative stress, can lead to several severe hematological disorders. Direct interactions of extracellular heme with alternative pathway complement components (APCCs) may be implicated molecularly in diverse conditions at sites of abnormal cell damage and vascular injury. In such disorders, a deregulated AP could be associated with the heme-mediated disruption of the physiological heparan sulphate–CFH coat of stressed cells and the induction of local hemostatic responses. Within this conceptual frame, a computational evaluation of HBMs (heme-binding motifs) aimed to determine how heme interacts with APCCs and whether these interactions are affected by genetic variation within putative HBMs. Combined computational analysis and database mining identified putative HBMs in all of the 16 APCCs examined, with 10 exhibiting disease-associated genetic (SNPs) and/or epigenetic variation (PTMs). Overall, this article indicates that among the pleiotropic roles of heme reviewed, the interactions of heme with APCCs could induce differential AP-mediated hemostasis-driven pathologies in certain individuals.

## 1. Introduction

### 1.1. Pleiotropic Functions of Heme, Transport and Heme-Associcated Pathologies

Heme (Fe^2+^-protoporphyrin IX) is a pigment of life in all organisms ranging from bacteria to mammals [1,2,3,4]. In terms of structure, heme exhibits a protoporphyrin IX tetrapyrrole ring system that is coordinated by a central iron ion through the four nitrogen atoms of the assembled moiety [5]. Heme also exhibits eight alkyl substituents (four methyl, two propionates and two vinyl groups) attached to its pyrrole rings. As a covalent prosthetic group in several vital hemoproteins, such as hemoglobins, myoglobins, cytochromes and enzymes, it serves as the essential gas carrier of oxygen (O_2_), nitrogen oxide (NO) and carbon monoxide (CO) [6,7,8,9].

In the hemoglobin chains, the iron ion is bound to a histidine residue and to oxygen which binds at the other coordinated position of iron. The iron ion in hemoglobin is in its ferrous state (Fe^2+^) facilitating the reversible association with molecular oxygen. When the oxidation of hemoglobin occurs, iron transitions to its ferric state (Fe^3+^), thus converting hemoglobin to methemoglobin, which has limited oxygen-carrying capacity. In the presence of chloride (Cl^−^) ions, heme is converted to hemin, the oxidized form of iron protoporphyrin IX [5]. Our current knowledge about the functions of heme has been derived from experimental work using hemin, the oxidized form of heme (Fe^3+^-protoporphyrin IX) with a chloride ligand.

Heme is a major activator and regulator of erythropoiesis [5,10,11,12], an essential constituent of the red blood cells (RBCs), and a central element in cellular metabolism and mitochondrial bioenergetics. In addition, heme contributes to globin biosynthesis [12,13], induces cell signaling and sensing pathways [14,15], and it also facilitates proteolysis via ubiquitination [14,15,16] among its several pleiotropic biological activities and properties summarized in Table 1 as examples.

Heme is synthesized de novo in the mitochondria [3,5,17], while it is catabolized by heme oxygenases (HOs) into bilirubin and CO_2_ [4,5,18,19,20]. Unfortunately, despite being essential for erythropoiesis and pivotal for several other molecular processes, heme as a free agent can be hazardous as a potent oxidant in the formation of volatile radical oxygen species (ROS) [14,21,22,23].

The diverse effects of heme suggest that under healthy conditions, its intracellular levels and trafficking are constantly monitored, and tightly regulated, by an extensively network of heme-binding proteins (HeBPs) [24,25,26,27,28,29,30]. These proteins are of diverse ontologies and contain often multiple heme-binding motifs (HBMs) that bind labile heme (biologically available and non-covalently bound) transiently with various affinities (K_d_) [1,5,26,31,32,33,34]. These classes of motifs exhibit a primary architecture such as X4(C/H/Y)^0^X4 and contain an amino acid, histidine (H), tyrosine (Y), or cysteine (C), coordinated to the iron ion of heme and surrounded by positively-charged amino acids or cysteine–proline motifs (CP motifs) or cysteine [35,36]. The transport of labile heme in and out of the cells is also achieved through its transient binding to several shuttle proteins, receptors and complexes [27,37,38,39]. Heme is extracellularly sequestered when damaged or ruptured cells release considerable amounts of hemoproteins and eventually labile heme into tissues, organs and into the circulation [22].

**Table 1:** Heme in diverse molecular processes and pathologies ^1^.

**Beneficial Effects (+)**
**Serves as prosthetic group in hemoproteins such as hemoglobin, myoglobin, cytochromes and enzymes** [1,2,3,4,5,10]
**Acts as a gas carrier for O_2_, CO and NO** [6,7,8,9]
**Enhances globin mRNA translation** [12,13]
**Induces hemoglobin biosynthesis and erythropoiesis** [5,11,13,40,41,42,43]
**Activates cell signaling and regulates sensing** [14,15]
**Regulates mitochondrial respiratory bioenergetics** [17,44,45]
**Binds to DNA G4 structural domains** [46]
**Regulates the transcriptional dynamics of several genes** [5,10]
**Activates chaperones such as the heat shock proteins HSP70 and HSP90** [47]
**Forms conjugation adducts with N-acetyl cysteine (NAC) and other thiols** [21]
**Harmful Effects (−)**
**Stimulates toll-like receptors (TLRs) affecting the immune response** [48,49]
**Regulates complement and coagulation responses** [50,51,52,53,54,55]
**Promotes ubiquitination and proteolysis** [14,16]
**Acts as a major oxidant promoting ROS accumulation and cell stress** [14,22,24]
**Stimulates stroke cell lysis and neuron ferroptosis** [56,57,58]
**Inhibits neuronal functions such as the low conductance K^+^ channels** [59,60]
**Heme-Associated Pathologies**
**Severe hematological disorders such as acute intermittent porphyrias** [61] **and anemias** [5] **that include congenital sideroblastic anemia** [62] **and Diamond–Blackfan anemia** [63,64]
**Inflammation** [48,49,65]
**Hemolytic syndromes** [66]
**Severe sepsis** [65]
**Stroke** [38,39,56,57,58]
**Neurodegeneration** [67,68]
**Neurological disorders** [69,70]
**Cardiovascular arrythmias** [71,72]
**Heme-Associated Complementopathies** [73]
**Hemostasis-driven thromboinflammation** [74]
**Paroxysmal nocturnal hemoglobinuria (PNH)** [75,76]
**Hemolytic diseases and cell lysis conditions such as hemolytic uremic syndromes, hemorrhage, sepsis and sickle cell disease** [53,77,78]
**Age-related macular degeneration (AMD)** [75,79,80]
**Ferroptosis in traumatic brain injury** [68]
**Ischemic stroke with cerebral hemorrhage** [81]
**Neurodegeneration** [82]
**Huntington’s disease** [83]

### 1.2. Interactions of Heme with Complement Components

**Figure 1:**
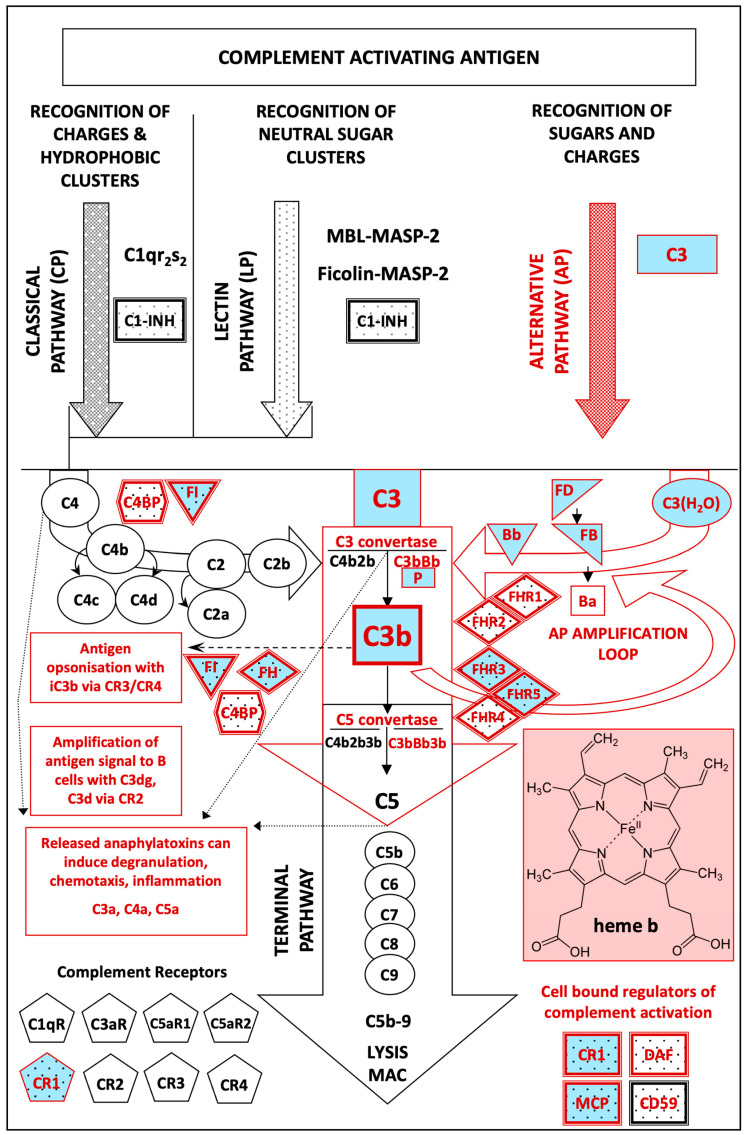
**A schematic representation of the human complement system.** The three activation pathways of the system (classical, lectin and alternative) are represented together with the terminal pathway that eventually leads to the formation of the membrane attack complex (MAC) after complement activation. Regulatory inhibitory proteins are shown in shaded boxes and complement receptors in pentagons. One positive regulator, properdin (P), which stabilizes the C3 convertase C3bBb is also shown. This study investigates the transient interactions of heme with the AP components and their effects on associated products and processes. Overall, 16 AP components, highlighted in red, appeared to contain putative HBMs, while 10 of them, highlighted in red and pale blue shades, contain putative HBMs which overlay with sites or residues that exhibit genetic variation (encoded SNPs: single nucleotide polymorphisms). Some of these variations may increase, directly or indirectly, the avidity of certain complement components for heme, increasing its capacity to deregulate the AP amplification loop dynamics in certain carriers of the mutations. The deregulation of the AP amplification loop by heme can facilitate a faster consumption of C3, inefficient opsonization and activation of hemostasis. Increased turnover of C3 can also gradually cripple the activation of the downstream terminal complement components. This can eventually lead to reduction in C5a anaphylatoxin release and limited or no formation of the terminal MAC. In such an asynchronous setting, C3a becomes dominant over C5a, as the main complement anaphylatoxin released locally and in circulation (C3a > > > C5a). The AP has multidimensional involvement in important immune [88,89,90] and hemostatic responses and associated processes [74]. Figure 1 is adapted from [97] with minor modifications.

Extracellularly, in plasma, heme is scavenged by hemopexin (HPX) [84], albumin and several other proteins [85], while it also interacts directly with the complement components C1q [55], C3 [54] and factor I [51] (Figure 1). These direct interactions influence the activation and regulation dynamics of the classical (CP) and alternative (AP) complement pathways. Heme can interact with C1q and inhibit the classical complement pathway that is typically associated with the specific recognition and tagging of surface blebs of apoptotic vascular endothelial cells [55,86,87]. In addition, the association of heme with C3 at sites of endothelial damage was found to downregulate the expression of CD46/MCP and CD55/DAF, thus limiting the decay accelerative capacity of the compromised cells mainly to locally available CFH, and therefore promoting the formation of a hyperactive AP C3 convertase [54]. The interaction of heme with CFI blocks its proteolytic capacity against C3b, therefore also supporting the formation of a hyperactive AP C3 convertase [51].

The AP has recently attracted renewed interest due to its multidimensional involvement in important immune [88,89,90] and hemostatic processes [74]. Interestingly and in terms of the competing biochemical dynamics between the CP and AP, recent data have suggested that the contribution of the AP in complement activation on cell surfaces depends on the strength of CP initiation [91]. In that perspective, a heme-crippled C1q can enhance the AP activation dynamics, if there is lack of effective decay accelerating activity to control the formation of a C3bBb convertase. 

Heme can downregulate CD46/MCP and CD55/DAF limiting the local decay accelerator factor potential to CFH, while it can also distort C3 [54] and block the proteolytic capacity of CFI [51]. The exposure of endothelia to heme can also promote the rapid exocytosis of Weibel–Palade bodies, the TLR4-dependent surface membrane expression of P-selectin known to bind C3b/C3(H_2_O) and trigger the AP, and the release of the prothrombotic von Willebrand factor [54,77]. The occurrence of local noncanonical AP activation and its association with the induction of thrombosis hemostatic responses has been recently discussed for SARS-CoV-2 infection in COVID-19 [92,93,94,95,96]. In both of these quite different scenarios, the heme-induced stress and the viral infection, the disruption of the physiological heparan sulphate–CFH coating could be a common and pivotal attribute for the maintenance of a deregulated AP amplification loop [79]. Other parameters in the host background, such as natural genetic variation (e.g., indels, SNPs) and epigenetic modifications (e.g., phosphorylation) of complement AP components, may also synergistically favor the enhanced assembly of a deregulated AP amplification loop.

### 1.3. Heme Interactions with APCCs and Complement Deregulation

Therefore, the direct extracellular interactions of heme with complement components, and in particular with AP complement components (APCCs), are of particular interest towards understanding molecularly, diverse heme-associated pathologies mediated by complement deregulation. Such heme-associated complementopathies [73] (Table 1) are characterized by cell populations or sites of abnormal cellular damage and vascular injury. This potential involvement of the AP activation as a mediator of disease pathologies, triggered by heme-induced stress, formed the conceptual basis for investigating the heme binding interactions with APCCs. Given the recent progress in the advanced computational prediction of HBMs in HeBPs, the questions of whether the APCCs carry putative HBMs and whether these HBMs overlay with sites or residues that may genetically (encoded SNPs) and/or epigenetically (PTMs: post-translational modifications) vary among individuals were assessed. Such natural variability could be interesting in explaining, mechanistically, a tendency towards the deregulation of the AP, identifying potential personalized biomarkers of susceptibility for advanced diagnostics and revealing common targets for personalized pharmacological intervention in a diverse range of diseases induced by poorly controlled heme-driven cell stress. Using the UniProt database, the HeMoQuest-WESA algorithms, as well as the PhosphoSitePlus and ClinVar databases, this study investigated combinatorically 16 unique genes encoding APCCs for the presence of HBMs, and identified 10 with sites of encoded SNP and mapped PTM variabilities. The results of this analysis as well as the nature of these parameters, their potential biochemical synergies and implications are presented in the results and discussed in this manuscript.

## 2. Computational Evaluation of Heme Interactions with APCCs

### 2.1. Identification of Putative HBMs in APCCs

In order to explore whether APCCs carry putative HBMs, the UniProt database (https://www.uniprot.org/, accessed on 19 October 2022) [98] was utilized first to retrieve the reviewed canonical as well as the alternative spliced encoded isoforms of the *C3*, *C4BPA, C4BPB*, *CFB*, *CFD*, *CFH*, *CFHR1*, *CFHR2*, *CFHR3*, *CFHR4*, *CFHR5*, *CFI*, *CFP*, *CR1*, *DAF* and *MCP* genes [16 genes, 46 full length sequences in total] [99]. Second, upon consideration, the two genes encoding the C4BP chains were also added as C4BP can act as a cofactor for the degradation of the activated C3b (Figure 1) [100]. All the retrieved protein sequences were then submitted to the HeMoQuest server (SeqD-HBM algorithm) (https://www.pharma.uni-bonn.de/www-en/pharmazeutische-biochemie-und-bioanalytik-en/hemoquest, accessed on 30 October 2022) [35,36] for the identification of putative heme-binding motifs.

All the submissions included the online option for solvent accessibility predicted by the WESA algorithm. WESA is a sequence-based solvent accessibility meta-predictor program that has been incorporated into HeMoQuest for the prediction of protein surfaces with exposed HBMs [101,102]. Using this forward approach described, the recovered protein sequences were screened for solvent-exposed HBMs. The sum of all the putative HBMs discovered, as well as their predicted affinities for heme, is reported in the original HeMoQuest report submitted in the Supplementary Materials of this manuscript.

The analysis of all the retrieved APCC protein sequences in the HeMoQuest server revealed the presence of several putative HBMs in each of the sequences examined, all with variable Net charges and Predicted K_d_ (μM) (Supplementary Materials and Figure 1). The majority of the putative HBMs appeared scattered in both the soluble as well as the cell-anchored receptors (Supplementary Materials). There are sequence regions that exhibit shorter (C3, CFB, CFHR3, CFHR4-2) and in a few cases extended (CFP, CFH, DAF-5, CR1-Intra and C4BPA) stretches of consecutive or overlapping HBMs. Interestingly, among all those examined, CFP appears to contain the highest percentage of putative HBM sequences compared to the overall length of the mature protein, with 158 out of the total 442 aa (~36%). In addition, CFD, CFHR4 and C4BPA appear to contain a putative HBM in their corresponding signal guidance sequences.

### 2.2. Exploration of Genetic and Epigenetic Variation in Putative HBMs of APCCs

To determine the biological significance of the putative HBMs identified in APCCs, the presence of residual genetic (encoded SNPs) and epigenetic (PTMs) variations of interest in any of the identified HBMs was reviewed. These two layers were explored on the conceptual basis that some of the HBMs may be located in regions, folds or domains of each molecular structure, which may contribute significantly to certain functions of each complement component. In terms of the analysis, the identified putative HBMs of each APCC were manually accessed through the UniProt database for the identification of only short disease-associated variants, such as missense SNPs which reside within any of the identified putative HBMs for each molecule. Nearly all of the disease-associated encoded SNPs curated in this study are reported in the dbSNP [103] and ClinVar [104] NCBI databases. All the findings and positionings of the curated HBM missense SNP variants in the protein sequences are also reported in the Supplementary Materials and Table 2 of this manuscript.

To further investigate the variations detected within the putative HBMs, the presence of any reported epigenetic marks in residues that lie within the identified putative HBMs was also explored through the PhosphoSitePlus (PSP) database [105]. For clarity reasons, among the marks reported in the PSP database, only the ones identified in multiple high- and low-throughput studies (≥5 cited references) were recorded. All the findings and positionings of the curated HBM PTM variants in the protein sequences are also reported in the Supplementary Materials and Table 2 of this manuscript.

The detection of genetic variations within the identified putative HBMs indicated the presence of clinical-disease-relevant missense SNPs in HBMs of 10 out of the initial 16 assessed genes (Figure 1 and Table 2). These are functionally relevant variants that have been identified through genomic studies of complement AP-mediated diseases as discussed earlier. The majority of these coding SNPs are located in the positions surrounding the residue with heme coordinating roles, while in some cases the latter may also vary (Table 2). Some of the HBM sequences listed in Table 2 exhibit variations in multiple residues and in some cases this could be important in terms of understanding, combinatorically, the changes in local motif microstructures and charges.

Although there is natural diversity in the amino acid substitutions listed in Table 2, several of the reported disease-associated variations can introduce structural and/or complement-relevant functional changes in each APCC. Such changes may affect either aspects of protein production, stability and secretion, or may function in relation to the physiologically complement-relevant partners or competitors. Some of these disease-relevant effects may also be charge-related, as some amino acid substitutions introduce point charge alterations depending on their associated neutral, basic or acidic R groups.

Some of the charge-associated and disease-related variations, however, may also be relevant for the dynamics of the HBMs to interact with excess of heme when available. As the HBMs–heme dynamics rely on coordinated electrostatic interactions, amino acid substitutions that introduce basic residues (R > K > H charge strength) can increase the potential of certain HBMs to bind heme more competitively. Reversibly, amino acid substitutions that replace basic residues with others that are non-charged probably weaken these interactions. In case there is more than one such substitution in each HBM, the effects are dependent on the overall charge change. Based on this concept, in Table 2, 12 unique red (●) or 15 unique green (●) marks were introduced to tag variants that probably strengthen (●/+) or weaken (●/−) the potential HBM–heme interaction dynamics. Hence, some of the red (●)-tagged variants in Table 2 may predispose to increased susceptibility of heme-induced and complement-mediated pathologies (Table 1). Reversibly, some of the green (●) tagged variants may protect from heme-induced and complement-mediated stress responses. Among the red (●)-tagged variants that introduce noticeable charge changes in the identified putative HBMs and are: **for C3: the rs1967565177 (C873R), for CFH: the rs201671665 (Q400K)-rs1061170 (Y402H), rs886039869 (C984R), VAR_025872 (C1043R), rs55679475 (Y1058H) and rs121913055 (L1189R), for CFI: the rs141853578 (G119R) and rs75612300 (H183R), 1 for CFP: the VAR_083039 (E244K) and 1 for CR1: the rs2274567 (H1208R)**.

In order to examine whether epigenetic marks of post-translational modifications (PTMs) could contribute mechanistically to variability in heme interaction at the protein level, similarly as for the SNPs/HBMs overlay, repeated marks of PTMs from the PhosphoSitePlus database were identified only in CFH (Table 2). In particular, this included the phosphorylation of threonine (T) 1193, which replaces a neutral hydroxyl group with a negatively charged phosphate. This phosphorylation introduces a potential decrease (−) in the overall charge of this HBM, which may weaken its affinity for heme. It is interesting to note that within the same HBM **^1186^KQKLYSRTG^1194^** located in the CCP/Sushi 20 domain (Table 2), the rs121913055 (L1189R) potentially increases (+) the local charge, while the rs761877050 (G1194D) potentially decreases (−) the local charge. Thus, in terms of the local motif architecture, the potential affinity of this HBM for heme may vary depending on its genetic and/or epigenetic variation among individuals.

## 3. The Conceptual Basis of Heme-Mediated Alternative Pathway Deregulation

The central core presented in this manuscript describes a conceptual effort to investigate the molecular interactions of cell stress released heme with complement components, and builds primarily on years of progress in understanding the multidimensional roles and dynamic interactions of heme in cells and tissues. As an approach, it is primarily based on advances in computational analysis for the identification of HBMs, as well as the database organization of recorded disease-relevant genomic variation and epigenetic marks. This effort expands on earlier findings and observations describing interactions of heme with specific complement components [51,54,55], and attempts to bridge them with the involvement of complement in inflammation and hemostasis at sites of abnormal cellular damage and vascular injury. As the biochemistry of complement components has been intensely investigated for decades and the majority of its structures have now been solved in high resolution [106,107], this computational evaluation aimed to explore the interactions of heme with all known APCCs, determine their impact in the AP as a system unit [88,89], and eventually uncover potential sources of genetic and epigenetic variability among individuals.

One of the initial findings that appeared interesting is the fact that although the study included the HeMoQuest analysis of isoforms encoded by 16 genes, only 10 of them exhibited HBMs that overlayed disease-associated SNPs (Section 2.2 and Table 2). This could be due to the lack of further additional mapped disease variants for the remaining candidates, or, unexpectedly, it could be mechanistically associated with a preferable tendency of heme to interact primarily first with a given set of APCCs with more reachable or more chemically active HBMs. As all solvent-exposed HBMs can be competitively occupied by heme, the presence of multiple putative HBMs indicates that when heme is in various excess gradients, it can bind through multiple nodes to all the components examined and compete with their physiologically relevant partners and competitors. This could be important for APCCs heavily coated with putative HBMs such as CFP (Section 2.1).

The binding of heme to certain HBMs may directly distort important physiological protein–protein interactions critical for complexes such as the one that stabilizes the critical AP C3bBb C3 convertase (Section 2.1 and Figure 1) [106], and/or reduces the catalytic activity of certain proteases such CFI [108], all potentially influencing aspects of AP regulation [89]. Other effects may be potentially indirect, as labile heme may distort the interactions of APCCs with other important cellular constituents such as heparan sulfate in the case of CFH [79,109]. Thus, in this context, the transient effects of labile heme and its associated disruptions are influenced by aspects of availability and chemical avidity of the HBMs for heme. As the approach described in this study does not examine the structural interactions of heme with every APCC at the atomic level, we cannot exclude from case-to-case indirect contributions from other factors such as contributions from distal or proximal folds, do-mains and residues.

**Figure 2:**
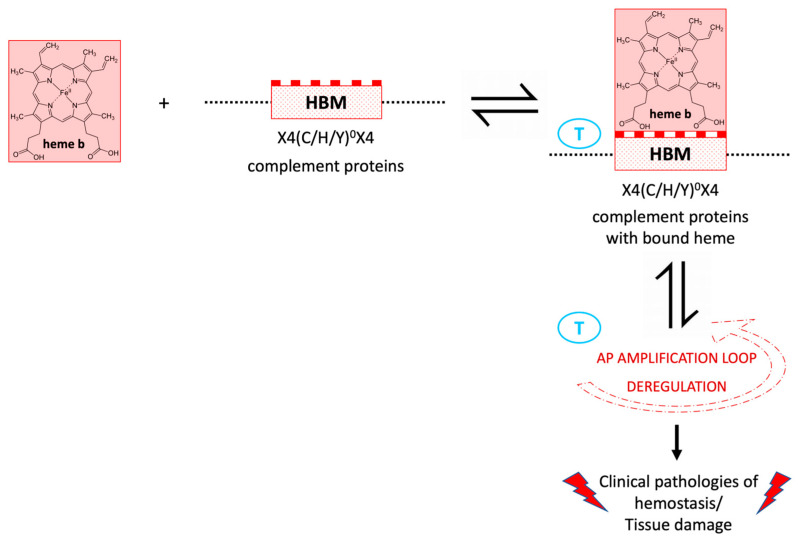
Schematic illustration of heme–complement protein interactions through heme-binding motifs (HBMs) that can lead to the deregulation of the alternative pathway (AP) amplification loop. In clinical pathologies characterized by the uncontrolled release of cell-stressed intracellular heme, heme can locally deplete complement C3 and induce activation of hemostasis responses. The oval Ts in blue indicate potential sites for therapeutic intervention, either by scavenging the excess of heme released, and/or by blocking the deregulation of the AP amplification loop using advanced next-generation complement inhibitors.

In terms of the HBM chemical potency for heme, SNP-encoded changes in the local charges may enhance the HBM–heme interaction dynamics (Figure 2). Clinically relevant and disease-associated missense SNPs in HBMs may genetically enhance the association dynamics with heme by encoding amino acid substitutions that enhance heme–complement protein interface interactions and increase the predisposition of certain individuals to AP deregulation upon certain suitable stimulation [89], as in aHUS [53], stroke [81] or neurodegeneration [82].

Among the red (●)-tagged variants described in Section 2.2 (Table 2), some appeared quite interesting, three of which were for **CFH**. The **rs201671665 (Q400K)** and **rs1061170 (Y402H)** variants reside very close in the same HBM within the **CCP/Sushi 7** domain of CFH, which physiologically interacts with various polyanions present in host cell surface groups such as sialic acids and glycosaminoglycans (GAGs), namely heparan sulfate [79,96,109]. Similarly, the **rs121913055 (L1189R)** is also located in an HBM within the C-terminal end **CCP/Sushi 20** domain of CFH, which can also physiologically interact with various polyanion ligands such as heparan sulfate and microbes [90,96,109]. Such interactions at the C-terminal end physiologically promote the oligomerization and deposition of CFH in a higher density on the surface of host cells, which facilitates the inactivation of C3b through the N-terminal region of factor H [109].

In individuals carrying these three CFH SNPs, heme might outcompete the host cell surface polyanions such as heparan sulfate and sialic acids [96]. Recombinant fragments of CFH with the Y402H polymorphism have shown impaired interaction with various ligands including heparin, C reactive protein, and fibromodulin; interestingly, the same polymorphism has also been intracellularly associated with reduced mitochondrial function, increased oxidative stress and the accumulation of oxidized lipids in AMD [79,109]. Such effects, which may include CFH in individuals with combinations of the three variants discussed, might also resemble more disease conditions similarly characterized by oxidative stress [56,57,58,68] (e.g., ferroptosis). In terms of disease mechanisms, the potential involvement of the AP could be associated with the heme-mediated disruption of the physiological heparan sulphate–CFH coating of cells undergoing ferroptosis, or suffering from significant heme-mediated oxidative stress. Such a disruption, could induce cell surface noncanonical AP activation in a heme-skewed microenvironment that promotes its deregulation.

Heme accumulated and released during cell damage may also compete with heparan sulphate for CFH and bind CFI. Τhe heme-mediated disruption of the physiological heparan sulphate–CFH coating of cells [96] could induce cell surface noncanonical AP activation in a heme-skewed microenvironment that favors deregulation leading to local C3 depletion. The presence of heme can downregulate CD46/MCP and CD55/DAF limiting the local decay accelerator factor potential to CFH, while it can also distort C3 [54] and block the proteolytic capacity of CFI [51]. The exposure of endothelia to heme can also promote the rapid exocytosis of Weibel–Palade bodies, the TLR4-dependent surface membrane expression of p-selectin known to bind C3b/C3(H_2_O) and trigger the AP, and the release of the prothrombotic von Willebrand factor [54,77].

Within a spin-off concept of the frame presented, CFH in individuals carrying combinations of the three variants discussed might be more prone and susceptible to competitive disruption by other non-polyanion ligands utilized by various microbes such as heme [90,92,95]. In this context, heme might be cleverly utilized by several microbes as a means of anchoring to target cells through CFH and specific cell-surface antigen (entry receptor) binding [94,95,96,110,111,112]. This mode probably also enables them to bypass the innate immune responses through various further means which may include C3 depletion by non-canonical AP deregulation and C3 convertase hijacking (Table 2 and Figure 2) [90,92].

The identified red (●)-tagged genetic variants of **CFI**, the **rs141853578 (G119R)** and **rs75612300 (H183R)**, are located in the **SRCR** non-catalytic heavy-chain domain and in certain individuals may increase the local affinity for heme. Computational modeling has indicated that the putative site of interaction of heme to factor I is at the interface between the heavy- and the light-chain of factor I [51], and thus may potentially affect its proteolytic activity and the integration with the C3b/factor H and, by homology, the C3b/CR1 complex [108,113]. The identified red (●)-tagged genetic variant **rs1967565177 (C873R)** of **C3** resides in the **MG7** domain of the α’-chain which is a binding region for several C3 inhibitors that block the assembly of the AP pro-convertase and the degradation of C3b by FI [106].

The **epigenetic phosphorylation of CFH at threonine (T) 1193**, which replaces a neutral hydroxyl group with a negatively charged phosphate, introduces a local charge decrease (−) that may limit its affinity for the negatively charged cell surface polyanions and/or heme. This may act as a functional switch influencing the oligomerization state of CFH and to a further extent its interactions with C3 and CFI. In past studies, phosphorylation marks on C3 after platelet activation had been found to promote the dynamics of the AP amplification loop [114,115] (Figure 1).

In terms of pharmacological targeting, if the identified red (●)-tagged variations increase the strength of interactions of the APCC putative HBMs with heme, one rational approach for handling oxidative stress and AP deregulation might aim at controlling and reducing the levels of heme in patients carrying combinations of the associated polymorphisms. This could be achieved with heme neutralizers such **NAC** that can act both intracellularly and extracellularly. Heme forms conjugation adducts with N-acetyl cysteine and glutathione [21,116], as well as others extracellularly with protein scavengers such as hemopexin and albumin. In concept, such small-molecule heme neutralizers could be utilized alongside selected AP complement inhibitors aimed at C3 [117,118,119], CFB and CFD [120,121,122], as well as potentially other APCCs [75]. Therefore, various potential therapeutic schemes could be custom designed to stabilize disease progression and potentially ameliorate some heme-associated clinical phenotypes (Table 1 and Table 2), if applied relatively early in individuals diagnosed with a predisposition to AP deregulation [123].

In conclusion, this article indicates that extracellular heme can interact directly with multiple APCCs through putative HBMs causing AP deregulation at sites of abnormal cell damage and vascular injury. Individuals carrying genetic variations that increase the strength of interactions of the APCC putative HBMs with heme may be more prone to developing complementopathies associated with the disruption of the physiological heparan sulphate–CFH coat of stressed cells and the induction of local hemostatic responses.

## Figures and Tables

**Table 2:** Identification of 10 APCCs with putative HBMs containing reported disease-associated encoding SNPs.

*Symbol*, Gene ID ^1,2^	Predicted HBMs ^3,4^	Corresponding SNPs ^4,5,6,7^	ClinVar/UniProt Disease Associations ^6^
***C3*,** **718**	**In P01024:**		
	**^150^** **FTVNH** **K** **LLP^158^,** **^734^** **L** **RR** **QHARASHLGLA^747^** **^869^** **NPAF** **C** **SLATTKR** **R** **HQQTV^886^** **^1097^** **SQV** **L** **CGAVK^1105^**	**rs147859257,155,K>Q [++>~]**(●) **rs578116271,736,R>Q [+++>~]**(●) **rs1967565177,873,C>R [~>+++](●)** **rs750654763,1100,L>P [~>~]**	**Age-related macular degeneration 9** **Atypical hemolytic-uremic syndrome with C3 anomaly, complement component 3 deficiency**
	**^734^** **L** **RR** **QHARASHLGLA^747^** **^1460^** **AFKV** **H** **QYFNVE^1470^**	**rs117793540,735,R>W [+++>~]**(●) **AR_063220,1464,H>D [+>--]**(●)	**Hemolytic uremic syndrome atypical 5 (AHUS5)**
	**^869^** **NPAF** **C** **SLATTKR** **R** **HQQTV^886^**	**rs1443451793,881,R>H [+++>+]**(●)	**Atypical hemolytic-uremic syndrome**
***CFB*,** **629**	**In P00751/P00751-2:**		
	**^299^** **KVASYGV** **K** **P^307^** **^504^** **PSKG** **H** **ESCM^512^**	**rs374738591,306,K>R [++>+++](●)** **rs138207668,508,H>Q [+>~]**(●)	**Atypical hemolytic-uremic syndrome with B factor anomaly** **Macular degeneration**
***CFD*,** **1675**	**In P00746:**		
	**^154^** **G** **I** **VNHAGRR^162^**	**rs373019471,155,I>V [~>~]**	**Recurrent Neisseria infections due to factor D deficiency**
***CFH*,** **3075**	**In P08603/P08603-2:**		
	**^295^** **RNGFYPAT** **R** **^303^**	**rs142937931,303,R>W [+++>~]**(●)	**Age-related macular degeneration 4** **CFH-Related dense deposit disease/membranoproliferative glomerulonephritis type II** **Hemolytic uremic syndrome, atypical, susceptibility to, 1**
	**^398^** **YN** **Q** **N** **Y** **GRKF^406^**	**rs201671665,400,Q>K [~>++](●)**	**Factor H deficiency**
	**^398^** **YN** **Q** **N** **Y** **GRKF^406^**	**rs1061170,402,Y>H [~>+](●)**	**Age-related macular degeneration 4** **Basal laminar drusen**
	**In P08603:**		
	**^759^** **I** **I** **LEEHLKNK^768^** **^807^** **Q** **I** **QLCPPPP^815^** **^976^** **EK** **W** **SHP** **P** **S** **C** **IKTDCLSLP^993^** **^1054^** **VQNA** **Y** **I** **V** **SR^1062^**	**rs772553879,760,I>L [~>~]** **rs752302466,808,I>M [~>~]** **rs149938052,982,P>S [~>~]** **rs55679475,1058,Y>H [~>+](●)** **rs55771831,1060,V>L [~>~]**	**Age-related macular degeneration 4** **Basal laminar drusen** **CFH-Related dense deposit disease/membranoproliferative glomerulonephritis type II** **Hemolytic uremic syndrome, atypical, susceptibility to, 1**
	**^976^** **EK** **W** **SHP** **P** **S** **C** **IKTDCLSLP^993^** **^1039^** **GRPT** **C** **RDTSCVNPP^1052^** **^1161^** **PK** **C** **LHPCV** **I** **^1169^** **^1186^** **KQK** **L** **Y** **S** **R** ** T ** **G** **^1194^** **^1208^** **SS** **R** **SHTL** **R** **TTCWDGK^1222^**	**VAR_025870,978,W>C [~>~]** **rs886039869,984,C>R [~>+++](●)** **VAR_025872,1043,C>R [~>+++](●)** **VAR_025878,1163,C>W [~>~]** **VAR_063650,1169,I>L [~>~]** **rs121913055,1189,L>R [~>+++](●)** **rs460897,1191,S>L [~>~]** **T1193,phosphorylation ^5^** **rs761877050,1194,G>D [~>--]** **rs121913059,1210,R>C [+++>~]**(●) rs121913051,1215,R>*/R>G [+++>~](●)	**Hemolytic uremic syndrome, atypical, susceptibility to, 1**
	**^1208^** **SS** **R** **SHTL** **R** **TTCWDGK^1222^**	**rs121913059,1210,R>C [+++>~]**(●) rs121913051,1215,R>* VAR_025887,1215,R>Q [+++>~](●)	**CFH-Related disorders** **Complement factor H deficiency**
***CFHR3*,** **10878**	**In Q02985:**		**Age-related macular degeneration 1**
	**^260^** **EPPRCIHP** **C** **IITE^272^**	**rs745503234,268,C>F [~/~]**	
	**In Q02985-2:**		
	**^199^** **EPPRCIHP** **C** **II^209^**	**rs745503234,207,C>F [~/~]**	
***CFHR5*,** **81494**	**In Q9BXR6:**		
	**^25^** **F** **P** **KIHHGFLY^34^** **^136^** **TPPICSFT** **K** **GECHVPIL^152^**	**rs1653577983,26,P>S [~>~]** **rs181511327,144,K>N [++>~]**(●)	**CFH-Related dense deposit disease/membranoproliferative glomerulonephritis type II**
***CFI*,** **3426**	**In P05156:**		
	**^91^** **LECLHPG** **T** **K^99^** **^330^** **KNRMHI** **R** **RK^338^**	**rs1478686846,98,T>A [~>~]** rs759676430,336,R>*	**Atypical hemolytic-uremic syndrome**
	**^114^** **VSLKH** **G** **NTD^122^**	**rs141853578,119,G>R [~>+++](●)**	**Atypical hemolytic-uremic syndrome 3 (AHUS3)** **Age-related macular degeneration-13 (ARMD13)**
	**^179^** **TECL** **H** **VHC** **R** **GL^189^**	**rs75612300,183,H>R [+>+++](●)**	**Hemolytic uremic syndrome atypical 3 (AHUS3)**
	**^179^** **TECL** **H** **VHC** **R** **GL^189^** **^261^** **G** **KGFHCKSG^269^** **^369^** **YI** **G** **G** **C** **WILT^377^** **^384^** **ASK** **T** **H** **R** **YQI^392^** **^567^** **DWI** **S** **YHVGRP^576^**	rs368615806,187,R>* rs143366614,187,R>Q [+++>~](●) **rs547901965,261,G>S [~/~]** **rs763931500,371,G>V [~/~]** **rs1579173999,373,C>S [~/~]** **rs1373768125,387,T>I [~/~]** **rs1292929833,389,R>C [+++>~]**(●) **rs200973120,570,S>T [~/~]**	**Atypical hemolytic-uremic syndrome with I factor anomaly**
***CFP*,** **5199**	**In P27918:**		
	**^97^** **SQL** **R** **YRRCV^105^**	**rs132630259,100,R>W [+++>~]**(●)	**Properdin deficiency, types I–III**
	**^161^** **R** **ACNHPAPKCGGHCPGQ^177^** **^201^** **PWTPC** **S** **ASCHGGPHEPKE^218^**	rs132630258,161,R>* rs132630260,206,S>*	**Properdin deficiency, X-linked**
	**^239^** **PGLAY** **E** **QRRCTGLP^252^**	**VAR_083039,244,E>K [-->++](●)**	**Properdin deficiency, type II**
	**^410^** **LLPK** **Y** **PPTV^418^**	**rs132630261,414,Y>D [~>--]**(●)	**Properdin deficiency, type III**
***CD35/CR1*,** **1378**	In P17927 (CR1*1/A/F):		**Malaria, severe, resistance to**
	**^1208^** **H** **TPSHQDNF^1216^**	**rs2274567,1208,H>R [+>+++](●)**	
	In E9PDY4 (CR1*2/B/S):		
	**^1658^** **H** **TPSHQDNF^1666^**	**rs2274567,1658,H>R [+>+++](●)**	
***CD46/MCP*,** **4179**	**In P15529/P15529-2** **→** **15:**		**Atypical hemolytic-uremic syndrome with MCP/CD46 anomaly**
	**^80^** **CD** **R** **NHTWLP^88^**	**rs761000846,82,R>Q [+++>~]**(●)	
	**In P15529:**		
	**^317^** **PRPTYKP** **P** **V^325^**	**rs41317833,324,P>L [~>~]**	
	**In P15529-3/-8/-11:**		
	**^302^** **PRPTYKP** **P** **V^310^**	**rs41317833,309,P>L [~>~]**	
	**In P15529-4/-9/-12:**		
	**^287^** **PRPTYKP** **P** **V^295^**	**rs41317833,294,P>L [~>~]**	
	**In P15529-7/-15:**		
	**^283^** **CLKGY** **P** **KPE^291^**	**rs886045838,288,P>A [~>~]**	

Notes: ^1^. **NCBI Gene** database, ^2^. **In red:** 10 genes encoding complement components of the alternative pathway that contain putative heme-binding motifs with disease associated SNPs as reported in the **NCBI ClinVar** and **UniProt databases**, ^3^. The numberings on the listed HBMs indicate the position of each motif in each encoded isoform as listed in the **UniProt database**, ^4^. **Highlighted in closed boxes** are SNPs that encode for amino acid substitutions in positions occupied by cysteines (C), histidines (H) or tyrosines (Y) that have **heme coordinating roles** within the corresponding HBMs, as reported in the **HeMoQuest** analysis report, ^5^. **Underlined** are residues in HBMs that exhibit post-translational variation with repeatedly detected (≥5 references) epigenetic marks of post-translational modifications as reported in the **PhosphoSitePlus** database, ^6^. Disease conditions in the **NCBI ClinVar** and **UniProt** databases associated with the identified SNPs of interest, ^7^. A total of 12 unique red (●) or 15 unique green (●) marks were introduced to tag variants that probably strengthen (●/+) or weaken (●/−) the potential HBM–heme dynamics. In this context, some of the red (●)-tagged variants may predispose to increased susceptibility to heme-induced and complement-mediated pathologies, while some of the green (●)-tagged variants may protect from heme-induced and complement-mediated stress responses.

## Data Availability

All the data supporting the findings of this study are available within the paper and its supplementary information files.

## Supplementary Materials

The following supporting information can be downloaded at: https://www.mdpi.com/article/10.3390/cimb45060330/s1. All relevant data are submitted along with the manuscript material.

## Abbreviations

AMD: age-related macular degeneration, AP: alternative pathway, APCCs: alternative pathway complement components, C3: complement component C3, C4BP: C4b binding protein, CFB: complement factor B, CFD: complement factor D, CFH: complement factor H, CFHR: complement factor H-related protein, CFI: complement factor I, CFP: complement factor properdin, CO: carbon monoxide, CP: classical pathway, CP motifs: cysteine–proline motifs, DAF: decay-accelerating factor, GAGs: glycosaminoglycans, HeBPs: heme-binding proteins, HBMs: heme-binding motifs, HO: heme oxygenase, HPX: hemopexin, MAC: membrane attack complex, MCP: membrane cofactor protein, NAC: N-acetyl cysteine, PNH: paroxysmal nocturnal hemoglobinuria, PSP: PhosphoSitePlus database, PTMs: post-translational modifications, RBC: red blood cell, ROS: reactive oxygen species, SNPs: single nucleotide polymorphisms, SRCR: scavenger receptor cysteine-rich domain, TLR: toll-like receptor, WESA: weighted ensemble solvent accessibility algorithm.
