# Supplementary material for: Heme Interactions as Regulators of the Alternative Pathway Complement Responses and Implications for Heme-Associated Pathologies
Source: Curr Issues Mol Biol. 2023 Jun 16;45(6):5198–214. doi: 10.3390/cimb45060330 (PMC10297344; doi:10.3390/cimb45060330)
Supplement: Supplementary file 1 [file cimb-2412718-supplementary.pdf › cimb-2412718-supplementary.pdf]

# HeMoQuest heme-binding prediction report for the submission number 1641

**Submitted on** Oct. 30, 2022, 7:42 a.m.

**Current status of the job**

As of Sun Oct 30 2022 20:24:42 GMT+0200 (EET)

**45** out of **46** sequences have been processed.

Update Results

The full analysis for the job can be downloaded as plain text, once all sequences are processed here.

## Results for the sequence(s) successfully processed:

### 1. sp|P01024|CO3_HUMAN Complement C3 OS=Homo sapiens OX=9606 GN=C3 PE=1 SV=2

```
MGPTSGPSLLLLLLTHLPLALGSPMYSIITPNILRLESEETMVLEAHDAQGDVPVTVTVHDFPGKKLVLS
SEKTVLTPATNHMGNVTFTIPANREFKSEKGRNKFVTVQATFGTQVVEKVVLVSLQSGYLFIQTDKTIYT
PGSTVLYRIFTVNHKLLPVGRTVMVNIENPEGIPVKQDSLSSQNQLGVLPLSWDIPELVNMGQWKIRAYY
ENSPQQVFSTEFEVKEYVLPSFEVIVEPTEKFYYIYNEKGLEVTITARFLYGKKVEGTAFVIFGIQDGEQ
RISLPESLKRIPIEDGSGEVVLSRKVLLDGVQNPRAEDLVGKSLYVSATVILHSGSDMVQAERSGIPIVT
SPYQIHFTKTPKYFKPGMPFDLMVFVTNPDGSPAYRVPVAVQGEDTVQSLTQGDGVAKLSINTHPSQKPL
SITVRTKKQELSEAEQATRTMQALPYSTVGNSNNYLHLSVLRTELRPGETLNVNFLLRMDRAHEAKIRYY
TYLIMNKGRLLKAGRQVREPGQDLVVLPLSITTDFIPSFRLVAYYTLIGASGQREVVADSVWVDVKDSCV
GSLVVKSGQSEDRQPVPGQQMTLKIEGDHGARVVLVAVDKGVFVLNKKNKLTQSKIWDVVEKADIGCTPG
SGKDYAGVFSDAGLTFTSSSGQQTAQRAELQCPQPAARRRRSVQLTEKRMDKVGKYPKELRKCCEDGMRE
NPMRFSCQRRTRFISLGEACKKVFLDCCNYITELRRQHARASHLGLARSNLDEDIIAEENIVSRSEFPES
WLWNVEDLKEPPKNGISTKLMNIFLKDSITTWEILAVSMSDKKGICVADPFEVTVMQDFFIDLRLPYSVV
RNEQVEIRAVLYNYRQNQELKVRVELLHNPAFCSLATTKRRHQQTVTIPPKSSLSVPYVIVPLKTGLQEV
EVKAAVYHHFISDGVRKSLKVVPEGIRMNKTVAVRTLDPERLGREGVQKEDIPPADLSDQVPDTESETRI
LLQGTPVAQMTEDAVDAERLKHLIVTPSGCGEQNMIGMTPTVIAVHYLDETEQWEKFGLEKRQGALELIK
KGYTQQLAFRQPSSAFAAFVKRAPSTWLTAYVVKVFSLAVNLIAIDSQVLCGAVKWLILEKQKPDGVFQE
DAPVIHQEMIGGLRNNNEKDMALTAFVLISLQEAKDICEEQVNSLPGSITKAGDFLEANYMNLQRSYTVA
IAGYALAQMGRLKGPLLNKFLTTAKDKNRWEDPGKQLYNVEATSYALLALLQLKDFDFVPPVVRWLNEQR
YYGGGYGSTQATFMVFQALAQYQKDAPDHQELNLDVSLQLPSRSSKITHRIHWESASLLRSEETKENEGF
TVTAEGKGQGTLSVVTMYHAKAKDQLTCNKFDLKVTIKPAPETEKRPQDAKNTMILEICTRYRGDQDATM
SILDISMMTGFAPDTDDLKQLANGVDRYISKYELDKAFSDRNTLIIYLDKVSHSEDDCLAFKVHQYFNVE
LIQPGAVKVYAYYNLEESCTRFYHPEKEDGKLNKLCRDELCRCAEENCFIQKSDDKVTLEERLDKACEPG
VDYVYKTRLVKVQLSNDFDEYIMAIEQTIKSGSDEVQVGQQRTFISPIKCREALKLEEKKHYLMWGLSSD
FWGEKPNLSYIIGKDTWVEHWPEEDECQDEENQKQCQDLGAFTESMVVFGCPN
```

*Solvent accessibility predicted by WESA*

| S.No | Coord. residue | 9mer motif | Net charge | Comment | Predicted $K_D$ (µM) |
|---|---|---|---|---|---|
| 1 | H154 | FTVNHKLLP | +2 | | 1.92 |
| 2 | Y209 | KIRAYYENS | +1 | | 4.45 |
| 3 | H356 | PYQIHFTKT | +2 | | 5.29 |
| 4 | Y385 | GSPAYRVPV | +1 | | 13.65 |
| 5 | H414 | SINTHPSQK | +2 | | 2.62 |
| 6 | H483 | MDRAHEAKI | +1 | | 3.63 |
| 7 | H738 | LRRQHARAS | +4 | | 1.95 |
| 8 | H743 | ARASHLGLA | +2 | | 8.08 |
| 9 | C873 | NPAFCSLAT | 0(CYS motif) | | 2.16 |
| 10 | H882 | TKRRHQQTV | +4 | | 7.39 |
| 11 | Y917 | KAAVYHHFI | +3 | | 3.82 |
| 12 | H918 | AAVYHHFIS | +2 | | 4.12 |
| 13 | H919 | AVYHHFISD | +1 | | 2.99 |
| 14 | H1002 | ERLKHLIVT | +2 | | 4.48 |
| 15 | C1101 | SQVLCGAVK | +1 | | 1.93 |
| 16 | H1312 | THRIHWESA | +2 | | 7.98 |
| 17 | H1464 | AFKVHQYFN | +2 | | 7.16 |
| 18 | Y1466 | KVHQYFNVE | +1 | | 2.7 |
| 19 | H1494 | TRFYHPEKE | +1 | | 6.03 |
| 20 | H1601 | EEKKHYLMW | +1 | | 2.35 |

### 2. sp|P00751|CFAB_HUMAN Complement factor B OS=Homo sapiens OX=9606 GN=CFB PE=1 SV=2

```
MGSNLSPQLCLMPFILGLLSGGVTTTPWSLARPQGSCSLEGVEIKGGSFRLLQEGQALEYVCPSGFYPYP
VQTRTCRSTGSWSTLKTQDQKTVRKAECRAIHCPRPHDFENGEYWPRSPYYNVSDEISFHCYDGYTLRGS
ANRTCQVNGRWSGQTAICDNGAGYCSNPGIPIGTRKVGSQYRLEDSVTYHCSRGLTLRGSQRRTCQEGGS
WSGTEPSCQDSFMYDTPQEVAEAFLSSLTETIEGVDAEDGHGPGEQQKRKIVLDPSGSMNIYLVLDGSDS
IGASNFTGAKKCLVNLIEKVASYGVKPRYGLVTYATYPKIWVKVSEADSSNADWVTKQLNEINYEDHKLK
SGTNTKKALQAVYSMMSWPDDVPPEGWNRTRHVIILMTDGLHNMGGDPITVIDEIRDLLYIGKDRKNPRE
DYLDVYVFGVGPLVNQVNINALASKKDNEQHVFKVKDMENLEDVFYQMIDESQSLSLCGMVWEHRKGTDY
HKQPWQAKISVIRPSKGHESCMGAVVSEYFVLTAAHCFTVDDKEHSIKVSVGGEKRDLEIEVVLFHPNYN
INGKKEAGIPEFYDYDVALIKLKNKLKYGQTIRPICLPCTEGTTRALRLPPTTTCQQQKEELLPAQDIKA
LFVSEEEKKLTRKEVYIKNGDKKGSCERDAQYAPGYDKVKDISEVVTPRFLCTGGVSPYADPNTCRGDSG
GPLIVHKRSRFIQVGVISWGVVDVCKNQKRQKQVPAHARDFHINLFQVLPWLKEKLQDEDLGFL
```

*Solvent accessibility predicted by WESA*

| S.No | Coord. residue | 9mer motif | Net charge | Comment | Predicted $K_D$ (µM) |
|---|---|---|---|---|---|
| 1 | H102 | CRAIHCPRP | +3 | Possible S-S bond | 7.36 |
| 2 | Y120 | PRSPYYNVS | +1 | | 0.27 |
| 3 | Y303 | KVASYGVKP | +2 | | 2.36 |
| 4 | Y317 | TYATYPKIW | +1 | | 1.24 |
| 5 | H347 | NYEDHKLKS | +1 | | 5.49 |
| 6 | H484 | MVWEHRKGT | +2 | | 5.13 |
| 7 | Y490 | KGTDYHKQP | +2 | | 3.45 |
| 8 | H491 | GTDYHKQPW | +1 | | 4.25 |
| 9 | H508 | PSKGHESCM | +1 | Possible S-S bond | 6.2 |
| 10 | Y646 | RKEVYIKNG | +2 | | 3.51 |
| 11 | Y666 | YAPGYDKVK | +1 | | 2.93 |
| 12 | H706 | PLIVHKRSR | +4 | | 1.84 |
| 13 | H737 | QVPAHARDF | +1 | | 0.16 |

### 3. sp|P00751-2|CFAB_HUMAN Isoform 2 of Complement factor B OS=Homo sapiens OX=9606 GN=CFB

```
MGSNLSPQLCLMPFILGLLSGGVTTTPWSLARPQGSCSLEGVEIKGGSFRLLQEGQALEYVCPSGFYPYP
VQTRTCRSTGSWSTLKTQDQKTVRKAECRAIHCPRPHDFENGEYWPRSPYYNVSDEISFHCYDGYTLRGS
ANRTCQVNGRWSGQTAICDNGAGYCSNPGIPIGTRKVGSQYRLEDSVTYHCSRGLTLRGSQRRTCQEGGS
WSGTEPSCQDSFMYDTPQEVAEAFLSSLTETIEGVDAEDGHGPGEQQKRKIVLDPSGSMNIYLVLDGSDS
IGASNFTGAKKCLVNLIEKVASYGVKPRYGLVTYATYPKIWVKVSEADSSNADWVTKQLNEINYEDHKLK
SGTNTKKALQAVYSMMSWPDDVPPEGWNRTRHVIILMTDGLHNMGGDPITVIDEIRDLLYIGKDRKNPRE
DYLDVYVFGVGPLVNQVNINALASKKDNEQHVFKVKDMENLEDVFYQMIDESQSLSLCGMVWEHRKGTDY
HKQPWQAKISVIRPSKGHESCMGAVVSEYFVLTAAHCFTVDDKEHSIKVSVGKDATEGPGLHLCSPGNTS
HFLQILHSTHPQCSPIPCTPDQSGMGEDVKLGMTRGQRQEAAHKEVVPTLLLQEGRSGTWR
```

*Solvent accessibility predicted by WESA*

| S.No | Coord. residue | 9mer motif | Net charge | Comment | Predicted $K_D$ (µM) |
|---|---|---|---|---|---|
| 1 | H102 | CRAIHCPRP | +3 | Possible S-S bond | 7.07 |
| 2 | Y120 | PRSPYYNVS | +1 | | 4.66 |
| 3 | H190 | SVTYHCSRG | +2 | Possible S-S bond | 9.23 |
| 4 | Y303 | KVASYGVKP | +2 | | 1.54 |
| 5 | Y317 | TYATYPKIW | +1 | | 6.36 |
| 6 | H347 | NYEDHKLKS | +1 | | 4.91 |
| 7 | H484 | MVWEHRKGT | +2 | | 2.99 |
| 8 | Y490 | KGTDYHKQP | +2 | | 8.93 |
| 9 | H491 | GTDYHKQPW | +1 | | 3.87 |
| 10 | H508 | PSKGHESCM | +1 | Possible S-S bond | 0.71 |
| 11 | H570 | LHSTHPQCS | +2 | Possible S-S bond | 4.92 |

### 4. sp|P05156|CFAI_HUMAN Complement factor I OS=Homo sapiens OX=9606 GN=CFI PE=1 SV=2

```
MKLLHVFLLFLCFHLRFCKVTYTSQEDLVEKKCLAKKYTHLSCDKVFCQPWQRCIEGTCVCKLPYQCPKN
GTAVCATNRRSFPTYCQQKSLECLHPGTKFLNNGTCTAEGKFSVSLKHGNTDSEGIVEVKLVDQDKTMFI
CKSSWSMREANVACLDLGFQQGADTQRRFKLSDLSINSTECLHVHCRGLETSLAECTFTKRRTMGYQDFA
DVVCYTQKADSPMDDFFQCVNGKYISQMKACDGINDCGDQSDELCCKACQGKGFHCKSGVCIPSQYQCNG
EVDCITGEDEVGCAGFASVTQEETEILTADMDAERRRIKSLLPKLSCGVKNRMHIRRKRIVGGKRAQLGD
LPWQVAIKDASGITCGGIYIGGCWILTAAHCLRASKTHRYQIWTTVVDWIHPDLKRIVIEYVDRIIFHEN
YNAGTYQNDIALIEMKKDGNKKDCELPRSIPACVPWSPYLFQPNDTCIVSGWGREKDNERVFSLQWGEVK
LISNCSKFYGNRFYEKEMECAGTYDGSIDACKGDSGGPLVCMDANNVTYVWGVVSWGENCGKPEFPGVYT
KVANYFDWISYHVGRPFISQYNV
```

*Solvent accessibility predicted by WESA*

| S.No | Coord. residue | 9mer motif | Net charge | Comment | Predicted $K_D$ (µM) |
|---|---|---|---|---|---|
| 1 | H95 | LECLHPGTK | +1 | Possible S-S bond | 0.69 |
| 2 | H118 | VSLKHGNTD | +1 | | 2.18 |
| 3 | H183 | TECLHVHCR | +2 | Possible S-S bond | 16.46 |
| 4 | H185 | CLHVHCRGL | +3 | Possible S-S bond | 15.66 |
| 5 | H265 | GKGFHCKSG | +3 | Possible S-S bond | 10.84 |
| 6 | H334 | KNRMHIRRK | +6 | | 0.05 |
| 7 | C373 | YIGGCWILT | 0(CYS motif) | | 0.83 |
| 8 | H388 | ASKTHRYQI | +3 | | 0.05 |
| 9 | C444 | NKKDCELPR | +1 | | 2.41 |
| 10 | Y571 | DWISYHVGR | +1 | | 3.9 |
| 11 | H572 | WISYHVGRP | +2 | | 9.1 |

### 5. sp|P00746|CFAD_HUMAN Complement factor D OS=Homo sapiens OX=9606 GN=CFD PE=1 SV=5

```
MHSWERLAVLVLLGAAACAAPPRGRILGGREAEAHARPYMASVQLNGAHLCGGVLVAEQWVLSAAHCLED
AADGKVQVLLGAHSLSQPEPSKRLYDVLRAVPHPDSQPDTIDHDLLLLQLSEKATLGPAVRPLPWQRVDR
DVAPGTLCDVAGWGIVNHAGRRPDSLQHVLLPVLDRATCNRRTHHDGAITERLMCAESNRRDSCKGDSGG
PLVCGGVLEGVVTSGSRVCGNRKKPGIYTRVASYAAWIDSVLA
```

*Solvent accessibility predicted by WESA*

| S.No | Coord. residue | 9mer motif | Net charge | Comment | Predicted $K_D$ (µM) |
|---|---|---|---|---|---|
| 1 | H2 | ___MHSWER | +1 | | 4.31 |
| 2 | H158 | GIVNHAGRR | +3 | | 4.51 |
| 3 | H184 | NRRTHHDGA | +3 | | 4.76 |
| 4 | H185 | RRTHHDGAI | +3 | | 7.48 |

### 6. sp|P27918|PROP_HUMAN Properdin OS=Homo sapiens OX=9606 GN=CFP PE=1 SV=2

```
MITEGAQAPRLLLPPLLLLLTLPATGSDPVLCFTQYEESSGKCKGLLGGGVSVEDCCLNTAFAYQKRSGG
LCQPCRSPRWSLWSTWAPCSVTCSEGSQLRYRRCVGWNGQCSGKVAPGTLEWQLQACEDQQCCPEMGGWS
GWGPWEPCSVTCSKGTRTRRRACNHPAPKCGGHCPGQAQESEACDTQQVCPTHGAWATWGPWTPCSASCH
GGPHEPKETRSRKCSAPEPSQKPPGKPCPGLAYEQRRCTGLPPCPVAGGWGPWGPVSPCPVTCGLGQTME
QRTCNHPVPQHGGPFCAGDATRTHICNTAVPCPVDGEWDSWGEWSPCIRRNMKSISCQEIPGQQSRGRTC
RGRKFDGHRCAGQQQDIRHCYSIQHCPLKGSWSEWSTWGLCMPPCGPNPTRARQRLCTPLLPKYPPTVSM
VEGQGEKNVTFWGRPLPRCEELQGQKLVVEEKRPCLHVPACKDPEEEEL
```

*Solvent accessibility predicted by WESA*

| S.No | Coord. residue | 9mer motif | Net charge | Comment | Predicted $K_D$ (µM) |
|---|---|---|---|---|---|
| 1 | Y64 | TAFAYQKRS | +2 | | 18.81 |
| 2 | C72 | SGGLCQPCR | +1 | Possible S-S bond | 4.49 |
| 3 | C75 | LCQPCRSPR | +2 | Possible S-S bond | 4.78 |
| 4 | Y101 | SQLRYRRCV | +3 | Possible S-S bond | 2.51 |
| 5 | C111 | WNGQCSGKV | +1 | | 3.6 |
| 6 | H165 | RACNHPAPK | +3 | Possible S-S bond | 13.69 |
| 7 | C170 | PAPKCGGHC | +2 | Possible S-S bond | 3.91 |
| 8 | H173 | KCGGHCPGQ | +2 | Possible S-S bond | 3.76 |
| 9 | C190 | TQQVCPTHG | +1 | | 3.93 |
| 10 | C205 | PWTPCSASC | 0(CYS motif) | Possible S-S bond | 10.41 |
| 11 | C209 | CSASCHGGP | +1 | Possible S-S bond | 7.14 |
| 12 | H210 | SASCHGGPH | +2 | Possible S-S bond | 0.71 |
| 13 | H214 | HGGPHEPKE | +1 | | 1.37 |
| 14 | Y243 | PGLAYEQRR | +1 | | 3.18 |
| 15 | C248 | EQRRCTGLP | +1 | | 1.16 |
| 16 | H286 | RTCNHPVPQ | +2 | Possible S-S bond | 1.15 |
| 17 | H304 | ATRTHICNT | +2 | Possible S-S bond | 3.5 |
| 18 | C306 | RTHICNTAV | +2 | | 15.13 |
| 19 | H358 | KFDGHRCAG | +2 | Possible S-S bond | 0.56 |
| 20 | H369 | QDIRHCYSI | +1 | Possible S-S bond | 15.4 |
| 21 | C370 | DIRHCYSIQ | +1 | | 7.01 |
| 22 | Y371 | IRHCYSIQH | +3 | Possible S-S bond | 0.75 |
| 23 | H375 | YSIQHCPLK | +2 | Possible S-S bond | 4.0 |
| 24 | Y414 | LLPKYPPTV | +1 | | 1.26 |
| 25 | H457 | RPCLHVPAC | +2 | Possible S-S bond | 3.0 |

### 7. sp|P08603|CFAH_HUMAN Complement factor H OS=Homo sapiens OX=9606 GN=CFH PE=1 SV=4

```
MRLLAKIICLMLWAICVAEDCNELPPRRNTEILTGSWSDQTYPEGTQAIYKCRPGYRSLGNVIMVCRKGE
WVALNPLRKCQKRPCGHPGDTPFGTFTLTGGNVFEYGVKAVYTCNEGYQLLGEINYRECDTDGWTNDIPI
CEVVKCLPVTAPENGKIVSSAMEPDREYHFGQAVRFVCNSGYKIEGDEEMHCSDDGFWSKEKPKCVEISC
KSPDVINGSPISQKIIYKENERFQYKCNMGYEYSERGDAVCTESGWRPLPSCEEKSCDNPYIPNGDYSPL
RIKHRTGDEITYQCRNGFYPATRGNTAKCTSTGWIPAPRCTLKPCDYPDIKHGGLYHENMRRPYFPVAVG
KYYSYYCDEHFETPSGSYWDHIHCTQDGWSPAVPCLRKCYFPYLENGYNQNYGRKFVQGKSIDVACHPGY
ALPKAQTTVTCMENGWSPTPRCIRVKTCSKSSIDIENGFISESQYTYALKEKAKYQCKLGYVTADGETSG
SITCGKDGWSAQPTCIKSCDIPVFMNARTKNDFTWFKLNDTLDYECHDGYESNTGSTTGSIVCGYNGWSD
LPICYERECELPKIDVHLVPDRKKDQYKVGEVLKFSCKPGFTIVGPNSVQCYHFGLSPDLPICKEQVQSC
GPPPELLNGNVKEKTKEEYGHSEVVEYYCNPRFLMKGPNKIQCVDGEWTTLPVCIVEESTCGDIPELEHG
WAQLSSPPYYYGDSVEFNCSESFTMIGHRSITCIHGVWTQLPQCVAIDKLKKCKSSNLIILEEHLKNKKE
FDHNSNIRYRCRGKEGWIHTVCINGRWDPEVNCSMAQIQLCPPPPQIPNSHNMTTTLNYRDGEKVSVLCQ
ENYLIQEGEEITCKDGRWQSIPLCVEKIPCSQPPQIEHGTINSSRSSQESYAHGTKLSYTCEGGFRISEE
NETTCYMGKWSSPPQCEGLPCKSPPEISHGVVAHMSDSYQYGEEVTYKCFEGFGIDGPAIAKCLGEKWSH
PPSCIKTDCLSLPSFENAIPMGEKKDVYKAGEQVTYTCATYYKMDGASNVTCINSRWTGRPTCRDTSCVN
PPTVQNAYIVSRQMSKYPSGERVRYQCRSPYEMFGDEEVMCLNGNWTEPPQCKDSTGKCGPPPPIDNGDI
TSFPLSVYAPASSVEYQCQNLYQLEGNKRITCRNGQWSEPPKCLHPCVISREIMENYNIALRWTAKQKLY
SRTGESVEFVCKRGYRLSSRSHTLRTTCWDGKLEYPTCAKR
```

*Solvent accessibility predicted by WESA*

| S.No | Coord. residue | 9mer motif | Net charge | Comment | Predicted $K_D$ (µM) |
|---|---|---|---|---|---|
| 1 | H87 | RPCGHPGDT | +1 | Possible S-S bond | 3.79 |
| 2 | Y299 | RNGFYPATR | +2 | | 5.81 |
| 3 | C320 | PAPRCTLKP | +2 | | 12.54 |
| 4 | H332 | PDIKHGGLY | +1 | | 2.72 |
| 5 | Y336 | HGGLYHENM | +1 | | 2.1 |
| 6 | Y344 | MRRPYFPVA | +2 | | 2.47 |
| 7 | Y352 | AVGKYYSYY | +1 | | 0.13 |
| 8 | C389 | CLRKCYFPY | +2 | Possible S-S bond | 10.64 |
| 9 | Y390 | LRKCYFPYL | +2 | Possible S-S bond | 17.42 |
| 10 | Y402 | YNQNYGRKF | +2 | | 1.26 |
| 11 | C448 | RVKTCSKSS | +3 | | 11.86 |
| 12 | H728 | TMIGHRSIT | +2 | | 4.31 |
| 13 | C753 | KLKKCKSSN | +4 | | 13.83 |
| 14 | H764 | ILEEHLKNK | +1 | | 0.95 |
| 15 | C811 | QIQLCPPPP | 0(CYS motif) | | 3.01 |
| 16 | C864 | SIPLCVEKI | 0(CYS motif) | | 0.24 |
| 17 | H980 | EKWSHPPSC | +1 | Possible S-S bond | 7.09 |
| 18 | C989 | IKTDCLSLP | 0(CYS motif) | | 9.5 |
| 19 | C1043 | GRPTCRDTS | +1 | | 6.53 |
| 20 | C1048 | RDTSCVNPP | 0(CYS motif) | | 3.88 |
| 21 | Y1058 | VQNAYIVSR | +1 | | 1.72 |
| 22 | H1165 | PKCLHPCVI | +2 | Possible S-S bond | 3.35 |
| 23 | Y1190 | KQKLYSRTG | +3 | | 1.45 |
| 24 | H1212 | SSRSHTLRT | +3 | | 1.81 |
| 25 | C1218 | LRTTCWDGK | +1 | | 1.77 |

### 8. sp|P08603-2|CFAH_HUMAN Isoform 2 of Complement factor H OS=Homo sapiens OX=9606 GN=CFH

```
MRLLAKIICLMLWAICVAEDCNELPPRRNTEILTGSWSDQTYPEGTQAIYKCRPGYRSLGNVIMVCRKGE
WVALNPLRKCQKRPCGHPGDTPFGTFTLTGGNVFEYGVKAVYTCNEGYQLLGEINYRECDTDGWTNDIPI
CEVVKCLPVTAPENGKIVSSAMEPDREYHFGQAVRFVCNSGYKIEGDEEMHCSDDGFWSKEKPKCVEISC
KSPDVINGSPISQKIIYKENERFQYKCNMGYEYSERGDAVCTESGWRPLPSCEEKSCDNPYIPNGDYSPL
RIKHRTGDEITYQCRNGFYPATRGNTAKCTSTGWIPAPRCTLKPCDYPDIKHGGLYHENMRRPYFPVAVG
KYYSYYCDEHFETPSGSYWDHIHCTQDGWSPAVPCLRKCYFPYLENGYNQNYGRKFVQGKSIDVACHPGY
ALPKAQTTVTCMENGWSPTPRCIRVSFTL
```

*Solvent accessibility predicted by WESA*

| S.No | Coord. residue | 9mer motif | Net charge | Comment | Predicted $K_D$ (µM) |
|---|---|---|---|---|---|
| 1 | H87 | RPCGHPGDT | +1 | Possible S-S bond | 6.32 |
| 2 | Y299 | RNGFYPATR | +2 | | 7.45 |
| 3 | C320 | PAPRCTLKP | +2 | | 4.08 |
| 4 | H332 | PDIKHGGLY | +1 | | 7.37 |
| 5 | Y344 | MRRPYFPVA | +2 | | 2.69 |
| 6 | Y352 | AVGKYYSYY | +1 | | 3.93 |
| 7 | H371 | SYWDHIHCT | +1 | Possible S-S bond | 2.03 |
| 8 | C385 | PAVPCLRKC | +2 | Possible S-S bond | 4.69 |
| 9 | Y390 | LRKCYFPYL | +2 | Possible S-S bond | 10.0 |
| 10 | Y402 | YNQNYGRKF | +2 | | 6.65 |

### 9. sp|P15529|MCP_HUMAN Membrane cofactor protein OS=Homo sapiens OX=9606 GN=CD46 PE=1 SV=3

```
MEPPGRRECPFPSWRFPGLLLAAMVLLLYSFSDACEEPPTFEAMELIGKPKPYYEIGERVDYKCKKGYFY
IPPLATHTICDRNHTWLPVSDDACYRETCPYIRDPLNGQAVPANGTYEFGYQMHFICNEGYYLIGEEILY
CELKGSVAIWSGKPPICEKVLCTPPPKIKNGKHTFSEVEVFEYLDAVTYSCDPAPGPDPFSLIGESTIYC
GDNSVWSRAAPECKVVKCRFPVVENGKQISGFGKKFYYKATVMFECDKGFYLDGSDTIVCDSNSTWDPPV
PKCLKVLPPSSTKPPALSHSVSTSSTTKSPASSASGPRPTYKPPVSNYPGYPKPEEGILDSLDVWVIAVI
VIAIVVGVAVICVVPYRYLQRRKKKGTYLTDETHREVKFTSL
```

*Solvent accessibility predicted by WESA*

| S.No | Coord. residue | 9mer motif | Net charge | Comment | Predicted $K_D$ (µM) |
|---|---|---|---|---|---|
| 1 | Y53 | KPKPYYEIG | +1 | | 10.0 |
| 2 | Y70 | KGYFYIPPL | +1 | | 2.58 |
| 3 | H84 | CDRNHTWLP | +1 | Possible S-S bond | 6.93 |
| 4 | Y247 | GKKFYYKAT | +3 | | 10.09 |
| 5 | Y321 | PRPTYKPPV | +2 | | 8.99 |

### 10. sp|P15529-2|MCP_HUMAN Isoform B of Membrane cofactor protein OS=Homo sapiens OX=9606 GN=CD46

```
MEPPGRRECPFPSWRFPGLLLAAMVLLLYSFSDACEEPPTFEAMELIGKPKPYYEIGERVDYKCKKGYFY
IPPLATHTICDRNHTWLPVSDDACYRETCPYIRDPLNGQAVPANGTYEFGYQMHFICNEGYYLIGEEILY
CELKGSVAIWSGKPPICEKVLCTPPPKIKNGKHTFSEVEVFEYLDAVTYSCDPAPGPDPFSLIGESTIYC
GDNSVWSRAAPECKVVKCRFPVVENGKQISGFGKKFYYKATVMFECDKGFYLDGSDTIVCDSNSTWDPPV
PKCLKVLPPSSTKPPALSHSVSTSSTTKSPASSASGPRPTYKPPVSNYPGYPKPEEGILDSLDVWVIAVI
VIAIVVGVAVICVVPYRYLQRRKKKGKADGGAEYATYQTKSTTPAEQRG
```

*Solvent accessibility predicted by WESA*

| S.No | Coord. residue | 9mer motif | Net charge | Comment | Predicted $K_D$ (µM) |
|---|---|---|---|---|---|
| 1 | Y53 | KPKPYYEIG | +1 | | 1.28 |
| 2 | Y70 | KGYFYIPPL | +1 | | 0.94 |
| 3 | H84 | CDRNHTWLP | +1 | Possible S-S bond | 3.77 |
| 4 | Y247 | GKKFYYKAT | +3 | | 7.03 |

### 11. sp|P15529-11|MCP_HUMAN Isoform C of Membrane cofactor protein OS=Homo sapiens OX=9606 GN=CD46

```
MEPPGRRECPFPSWRFPGLLLAAMVLLLYSFSDACEEPPTFEAMELIGKPKPYYEIGERVDYKCKKGYFY
IPPLATHTICDRNHTWLPVSDDACYRETCPYIRDPLNGQAVPANGTYEFGYQMHFICNEGYYLIGEEILY
CELKGSVAIWSGKPPICEKVLCTPPPKIKNGKHTFSEVEVFEYLDAVTYSCDPAPGPDPFSLIGESTIYC
GDNSVWSRAAPECKVVKCRFPVVENGKQISGFGKKFYYKATVMFECDKGFYLDGSDTIVCDSNSTWDPPV
PKCLKVSTSSTTKSPASSASGPRPTYKPPVSNYPGYPKPEEGILDSLDVWVIAVIVIAIVVGVAVICVVP
YRYLQRRKKKGTYLTDETHREVKFTSL
```

*Solvent accessibility predicted by WESA*

| S.No | Coord. residue | 9mer motif | Net charge | Comment | Predicted $K_D$ (µM) |
|---|---|---|---|---|---|
| 1 | Y53 | KPKPYYEIG | +1 | | 0.32 |
| 2 | Y70 | KGYFYIPPL | +1 | | 19.15 |
| 3 | H84 | CDRNHTWLP | +1 | Possible S-S bond | 7.49 |
| 4 | Y247 | GKKFYYKAT | +3 | | 16.09 |
| 5 | Y306 | PRPTYKPPV | +2 | | 11.64 |

### 12. sp|P15529-3|MCP_HUMAN Isoform D of Membrane cofactor protein OS=Homo sapiens OX=9606 GN=CD46

```
MEPPGRRECPFPSWRFPGLLLAAMVLLLYSFSDACEEPPTFEAMELIGKPKPYYEIGERVDYKCKKGYFY
IPPLATHTICDRNHTWLPVSDDACYRETCPYIRDPLNGQAVPANGTYEFGYQMHFICNEGYYLIGEEILY
CELKGSVAIWSGKPPICEKVLCTPPPKIKNGKHTFSEVEVFEYLDAVTYSCDPAPGPDPFSLIGESTIYC
GDNSVWSRAAPECKVVKCRFPVVENGKQISGFGKKFYYKATVMFECDKGFYLDGSDTIVCDSNSTWDPPV
PKCLKVSTSSTTKSPASSASGPRPTYKPPVSNYPGYPKPEEGILDSLDVWVIAVIVIAIVVGVAVICVVP
YRYLQRRKKKGKADGGAEYATYQTKSTTPAEQRG
```

*Solvent accessibility predicted by WESA*

| S.No | Coord. residue | 9mer motif | Net charge | Comment | Predicted $K_D$ (µM) |
|---|---|---|---|---|---|
| 1 | Y53 | KPKPYYEIG | +1 | | 12.19 |
| 2 | Y70 | KGYFYIPPL | +1 | | 16.12 |
| 3 | H84 | CDRNHTWLP | +1 | Possible S-S bond | 3.97 |
| 4 | Y247 | GKKFYYKAT | +3 | | 1.56 |
| 5 | Y306 | PRPTYKPPV | +2 | | 2.96 |

### 13. sp|P15529-12|MCP_HUMAN Isoform E of Membrane cofactor protein OS=Homo sapiens OX=9606 GN=CD46

```
MEPPGRRECPFPSWRFPGLLLAAMVLLLYSFSDACEEPPTFEAMELIGKPKPYYEIGERVDYKCKKGYFY
IPPLATHTICDRNHTWLPVSDDACYRETCPYIRDPLNGQAVPANGTYEFGYQMHFICNEGYYLIGEEILY
CELKGSVAIWSGKPPICEKVLCTPPPKIKNGKHTFSEVEVFEYLDAVTYSCDPAPGPDPFSLIGESTIYC
GDNSVWSRAAPECKVVKCRFPVVENGKQISGFGKKFYYKATVMFECDKGFYLDGSDTIVCDSNSTWDPPV
PKCLKGPRPTYKPPVSNYPGYPKPEEGILDSLDVWVIAVIVIAIVVGVAVICVVPYRYLQRRKKKGTYLT
DETHREVKFTSL
```

*Solvent accessibility predicted by WESA*

| S.No | Coord. residue | 9mer motif | Net charge | Comment | Predicted $K_D$ (µM) |
|---|---|---|---|---|---|
| 1 | Y53 | KPKPYYEIG | +1 | | 7.32 |
| 2 | Y70 | KGYFYIPPL | +1 | | 3.86 |
| 3 | H84 | CDRNHTWLP | +1 | Possible S-S bond | 9.25 |
| 4 | C157 | KPPICEKVL | +1 | | 0.06 |
| 5 | Y247 | GKKFYYKAT | +3 | | 2.32 |
| 6 | Y291 | PRPTYKPPV | +2 | | 0.61 |

### 14. sp|P15529-4|MCP_HUMAN Isoform F of Membrane cofactor protein OS=Homo sapiens OX=9606 GN=CD46

```
MEPPGRRECPFPSWRFPGLLLAAMVLLLYSFSDACEEPPTFEAMELIGKPKPYYEIGERVDYKCKKGYFY
IPPLATHTICDRNHTWLPVSDDACYRETCPYIRDPLNGQAVPANGTYEFGYQMHFICNEGYYLIGEEILY
CELKGSVAIWSGKPPICEKVLCTPPPKIKNGKHTFSEVEVFEYLDAVTYSCDPAPGPDPFSLIGESTIYC
GDNSVWSRAAPECKVVKCRFPVVENGKQISGFGKKFYYKATVMFECDKGFYLDGSDTIVCDSNSTWDPPV
PKCLKGPRPTYKPPVSNYPGYPKPEEGILDSLDVWVIAVIVIAIVVGVAVICVVPYRYLQRRKKKGKADG
GAEYATYQTKSTTPAEQRG
```

*Solvent accessibility predicted by WESA*

| S.No | Coord. residue | 9mer motif | Net charge | Comment | Predicted $K_D$ (µM) |
|---|---|---|---|---|---|
| 1 | Y53 | KPKPYYEIG | +1 | | 1.53 |
| 2 | Y70 | KGYFYIPPL | +1 | | 0.67 |
| 3 | H84 | CDRNHTWLP | +1 | Possible S-S bond | 2.61 |
| 4 | C157 | KPPICEKVL | +1 | | 4.35 |
| 5 | Y247 | GKKFYYKAT | +3 | | 6.32 |
| 6 | Y291 | PRPTYKPPV | +2 | | 9.91 |

### 15. sp|P15529-13|MCP_HUMAN Isoform G of Membrane cofactor protein OS=Homo sapiens OX=9606 GN=CD46

```
MEPPGRRECPFPSWRFPGLLLAAMVLLLYSFSDACEEPPTFEAMELIGKPKPYYEIGERVDYKCKKGYFY
IPPLATHTICDRNHTWLPVSDDACYRETCPYIRDPLNGQAVPANGTYEFGYQMHFICNEGYYLIGEEILY
CELKGSVAIWSGKPPICEKVLCTPPPKIKNGKHTFSEVEVFEYLDAVTYSCDPAPGPDPFSLIGESTIYC
GDNSVWSRAAPECKVVKCRFPVVENGKQISGFGKKFYYKATVMFECDKGFYLDGSDTIVCDSNSTWDPPV
PKCLKVLPPSSTKPPALSHSVSTSSTTKSPASSASGYPKPEEGILDSLDVWVIAVIVIAIVVGVAVICVV
PYRYLQRRKKKGTYLTDETHREVKFTSL
```

*Solvent accessibility predicted by WESA*

| S.No | Coord. residue | 9mer motif | Net charge | Comment | Predicted $K_D$ (µM) |
|---|---|---|---|---|---|
| 1 | Y53 | KPKPYYEIG | +1 | | 0.97 |
| 2 | Y70 | KGYFYIPPL | +1 | | 4.69 |
| 3 | H84 | CDRNHTWLP | +1 | Possible S-S bond | 3.5 |
| 4 | Y247 | GKKFYYKAT | +3 | | 1.62 |

### 16. sp|P15529-5|MCP_HUMAN Isoform H of Membrane cofactor protein OS=Homo sapiens OX=9606 GN=CD46

```
MEPPGRRECPFPSWRFPGLLLAAMVLLLYSFSDACEEPPTFEAMELIGKPKPYYEIGERVDYKCKKGYFY
IPPLATHTICDRNHTWLPVSDDACYRETCPYIRDPLNGQAVPANGTYEFGYQMHFICNEGYYLIGEEILY
CELKGSVAIWSGKPPICEKVLCTPPPKIKNGKHTFSEVEVFEYLDAVTYSCDPAPGPDPFSLIGESTIYC
GDNSVWSRAAPECKVVKCRFPVVENGKQISGFGKKFYYKATVMFECDKGFYLDGSDTIVCDSNSTWDPPV
PKCLKVLPPSSTKPPALSHSVSTSSTTKSPASSASGYPKPEEGILDSLDVWVIAVIVIAIVVGVAVICVV
PYRYLQRRKKKGKADGGAEYATYQTKSTTPAEQRG
```

*Solvent accessibility predicted by WESA*

| S.No | Coord. residue | 9mer motif | Net charge | Comment | Predicted $K_D$ (µM) |
|---|---|---|---|---|---|
| 1 | Y53 | KPKPYYEIG | +1 | | 7.46 |
| 2 | Y70 | KGYFYIPPL | +1 | | 0.71 |
| 3 | H84 | CDRNHTWLP | +1 | Possible S-S bond | 3.86 |
| 4 | Y247 | GKKFYYKAT | +3 | | 1.38 |
| 5 | Y248 | KKFYYKATV | +3 | | 3.29 |

### 17. sp|P15529-14|MCP_HUMAN Isoform I of Membrane cofactor protein OS=Homo sapiens OX=9606 GN=CD46

```
MEPPGRRECPFPSWRFPGLLLAAMVLLLYSFSDACEEPPTFEAMELIGKPKPYYEIGERVDYKCKKGYFY
IPPLATHTICDRNHTWLPVSDDACYRETCPYIRDPLNGQAVPANGTYEFGYQMHFICNEGYYLIGEEILY
CELKGSVAIWSGKPPICEKVLCTPPPKIKNGKHTFSEVEVFEYLDAVTYSCDPAPGPDPFSLIGESTIYC
GDNSVWSRAAPECKVVKCRFPVVENGKQISGFGKKFYYKATVMFECDKGFYLDGSDTIVCDSNSTWDPPV
PKCLKVSTSSTTKSPASSASGYPKPEEGILDSLDVWVIAVIVIAIVVGVAVICVVPYRYLQRRKKKGTYL
TDETHREVKFTSL
```

*Solvent accessibility predicted by WESA*

| S.No | Coord. residue | 9mer motif | Net charge | Comment | Predicted $K_D$ (µM) |
|---|---|---|---|---|---|
| 1 | Y53 | KPKPYYEIG | +1 | | 1.09 |
| 2 | Y70 | KGYFYIPPL | +1 | | 0.32 |
| 3 | H84 | CDRNHTWLP | +1 | Possible S-S bond | 4.89 |
| 4 | Y247 | GKKFYYKAT | +3 | | 4.78 |

### 18. sp|P15529-6|MCP_HUMAN Isoform J of Membrane cofactor protein OS=Homo sapiens OX=9606 GN=CD46

```
MEPPGRRECPFPSWRFPGLLLAAMVLLLYSFSDACEEPPTFEAMELIGKPKPYYEIGERVDYKCKKGYFY
IPPLATHTICDRNHTWLPVSDDACYRETCPYIRDPLNGQAVPANGTYEFGYQMHFICNEGYYLIGEEILY
CELKGSVAIWSGKPPICEKVLCTPPPKIKNGKHTFSEVEVFEYLDAVTYSCDPAPGPDPFSLIGESTIYC
GDNSVWSRAAPECKVVKCRFPVVENGKQISGFGKKFYYKATVMFECDKGFYLDGSDTIVCDSNSTWDPPV
PKCLKVSTSSTTKSPASSASGYPKPEEGILDSLDVWVIAVIVIAIVVGVAVICVVPYRYLQRRKKKGKAD
GGAEYATYQTKSTTPAEQRG
```

*Solvent accessibility predicted by WESA*

| S.No | Coord. residue | 9mer motif | Net charge | Comment | Predicted $K_D$ (µM) |
|---|---|---|---|---|---|
| 1 | Y53 | KPKPYYEIG | +1 | | 12.13 |
| 2 | Y70 | KGYFYIPPL | +1 | | 0.16 |
| 3 | H84 | CDRNHTWLP | +1 | Possible S-S bond | 7.54 |
| 4 | Y247 | GKKFYYKAT | +3 | | 4.02 |

### 19. sp|P15529-15|MCP_HUMAN Isoform K of Membrane cofactor protein OS=Homo sapiens OX=9606 GN=CD46

```
MEPPGRRECPFPSWRFPGLLLAAMVLLLYSFSDACEEPPTFEAMELIGKPKPYYEIGERVDYKCKKGYFY
IPPLATHTICDRNHTWLPVSDDACYRETCPYIRDPLNGQAVPANGTYEFGYQMHFICNEGYYLIGEEILY
CELKGSVAIWSGKPPICEKVLCTPPPKIKNGKHTFSEVEVFEYLDAVTYSCDPAPGPDPFSLIGESTIYC
GDNSVWSRAAPECKVVKCRFPVVENGKQISGFGKKFYYKATVMFECDKGFYLDGSDTIVCDSNSTWDPPV
PKCLKGYPKPEEGILDSLDVWVIAVIVIAIVVGVAVICVVPYRYLQRRKKKGTYLTDETHREVKFTSL
```

*Solvent accessibility predicted by WESA*

| S.No | Coord. residue | 9mer motif | Net charge | Comment | Predicted $K_D$ (µM) |
|---|---|---|---|---|---|
| 1 | Y53 | KPKPYYEIG | +1 | | 1.8 |
| 2 | Y70 | KGYFYIPPL | +1 | | 1.9 |
| 3 | H84 | CDRNHTWLP | +1 | Possible S-S bond | 1.28 |
| 4 | C157 | KPPICEKVL | +1 | | 9.85 |
| 5 | Y247 | GKKFYYKAT | +3 | | 1.34 |
| 6 | Y248 | KKFYYKATV | +3 | | 0.18 |
| 7 | Y287 | CLKGYPKPE | +1 | Possible S-S bond | 3.82 |

### 20. sp|P15529-7|MCP_HUMAN Isoform L of Membrane cofactor protein OS=Homo sapiens OX=9606 GN=CD46

```
MEPPGRRECPFPSWRFPGLLLAAMVLLLYSFSDACEEPPTFEAMELIGKPKPYYEIGERVDYKCKKGYFY
IPPLATHTICDRNHTWLPVSDDACYRETCPYIRDPLNGQAVPANGTYEFGYQMHFICNEGYYLIGEEILY
CELKGSVAIWSGKPPICEKVLCTPPPKIKNGKHTFSEVEVFEYLDAVTYSCDPAPGPDPFSLIGESTIYC
GDNSVWSRAAPECKVVKCRFPVVENGKQISGFGKKFYYKATVMFECDKGFYLDGSDTIVCDSNSTWDPPV
PKCLKGYPKPEEGILDSLDVWVIAVIVIAIVVGVAVICVVPYRYLQRRKKKGKADGGAEYATYQTKSTTP
AEQRG
```

*Solvent accessibility predicted by WESA*

| S.No | Coord. residue | 9mer motif | Net charge | Comment | Predicted $K_D$ (µM) |
|---|---|---|---|---|---|
| 1 | Y53 | KPKPYYEIG | +1 | | 1.98 |
| 2 | Y70 | KGYFYIPPL | +1 | | 14.79 |
| 3 | H84 | CDRNHTWLP | +1 | Possible S-S bond | 3.2 |
| 4 | C157 | KPPICEKVL | +1 | | 6.38 |
| 5 | Y247 | GKKFYYKAT | +3 | | 11.47 |
| 6 | Y248 | KKFYYKATV | +3 | | 0.01 |
| 7 | Y287 | CLKGYPKPE | +1 | Possible S-S bond | 14.78 |

### 21. sp|P15529-8|MCP_HUMAN Isoform M of Membrane cofactor protein OS=Homo sapiens OX=9606 GN=CD46

```
MEPPGRRECPFPSWRFPGLLLAAMVLLLYSFSDACEEPPTFEAMELIGKPKPYYEIGERVDYKCKKGYFY
IPPLATHTICDRNHTWLPVSDDACYRETCPYIRDPLNGQAVPANGTYEFGYQMHFICNEGYYLIGEEILY
CELKGSVAIWSGKPPICEKVLCTPPPKIKNGKHTFSEVEVFEYLDAVTYSCDPAPGPDPFSLIGESTIYC
GDNSVWSRAAPECKVVKCRFPVVENGKQISGFGKKFYYKATVMFECDKGFYLDGSDTIVCDSNSTWDPPV
PKCLKVSTSSTTKSPASSASGPRPTYKPPVSNYPGYPKPEEGILDSLDVWVIAVIVIAIGKQMVELNMPL
TRLNQPLQQSREAE
```

*Solvent accessibility predicted by WESA*

| S.No | Coord. residue | 9mer motif | Net charge | Comment | Predicted $K_D$ (µM) |
|---|---|---|---|---|---|
| 1 | Y53 | KPKPYYEIG | +1 | | 13.66 |
| 2 | Y70 | KGYFYIPPL | +1 | | 12.72 |
| 3 | H84 | CDRNHTWLP | +1 | Possible S-S bond | 1.7 |
| 4 | Y247 | GKKFYYKAT | +3 | | 2.41 |
| 5 | Y306 | PRPTYKPPV | +2 | | 5.43 |

### 22. sp|P15529-9|MCP_HUMAN Isoform N of Membrane cofactor protein OS=Homo sapiens OX=9606 GN=CD46

```
MEPPGRRECPFPSWRFPGLLLAAMVLLLYSFSDACEEPPTFEAMELIGKPKPYYEIGERVDYKCKKGYFY
IPPLATHTICDRNHTWLPVSDDACYRETCPYIRDPLNGQAVPANGTYEFGYQMHFICNEGYYLIGEEILY
CELKGSVAIWSGKPPICEKVLCTPPPKIKNGKHTFSEVEVFEYLDAVTYSCDPAPGPDPFSLIGESTIYC
GDNSVWSRAAPECKVVKCRFPVVENGKQISGFGKKFYYKATVMFECDKGFYLDGSDTIVCDSNSTWDPPV
PKCLKGPRPTYKPPVSNYPGYPKPEEGILDSLDVWVIAVIVIAIGKQMVELNMPLTRLNQPLQQSREAE
```

*Solvent accessibility predicted by WESA*

| S.No | Coord. residue | 9mer motif | Net charge | Comment | Predicted $K_D$ (µM) |
|---|---|---|---|---|---|
| 1 | Y53 | KPKPYYEIG | +1 | | 1.62 |
| 2 | H84 | CDRNHTWLP | +1 | Possible S-S bond | 3.97 |
| 3 | C157 | KPPICEKVL | +1 | | 0.7 |
| 4 | Y247 | GKKFYYKAT | +3 | | 3.12 |
| 5 | Y248 | KKFYYKATV | +3 | | 3.56 |
| 6 | Y291 | PRPTYKPPV | +2 | | 8.85 |

### 23. sp|P15529-10|MCP_HUMAN Isoform 2 of Membrane cofactor protein OS=Homo sapiens OX=9606 GN=CD46

```
MEPPGRRECPFPSWRFPGLLLAAMVLLLYSFSDACEEPPTFEAMELIGKPKPYYEIGERVDYKCKKGYFY
IPPLATHTICDRNHTWLPVSDDACYRETCPYIRDPLNGQAVPANGTYEFGYQMHFICNEGYYLIGEEILY
CELKGSVAIWSGKPPICEKVLCTPPPKIKNGKHTFSEVEVFEYLDAVTYSCDPAPGPDPFSLIGESTIYC
GDNSVWSRAAPECKVVKCRFPVVENGKQISGFGKKFYYKATVMFECDKGFYLDGSDTIVCDSNSTWDPPV
PKCLKVLPPSSTKPPALSHSTTKSPASSASGPRPTYKPPVSNYPGYPKPEEGILDSLDVWVIAVIVIAIV
VGVAVICVVPYRYLQRRKKKGTYLTDETHREVKFTSL
```

*Solvent accessibility predicted by WESA*

| S.No | Coord. residue | 9mer motif | Net charge | Comment | Predicted $K_D$ (µM) |
|---|---|---|---|---|---|
| 1 | Y53 | KPKPYYEIG | +1 | | 0.09 |
| 2 | Y70 | KGYFYIPPL | +1 | | 0.29 |
| 3 | H84 | CDRNHTWLP | +1 | Possible S-S bond | 2.52 |
| 4 | Y247 | GKKFYYKAT | +3 | | 1.59 |
| 5 | H299 | PALSHSTTK | +2 | | 2.47 |

### 24. sp|P15529-16|MCP_HUMAN Isoform 3 of Membrane cofactor protein OS=Homo sapiens OX=9606 GN=CD46

```
MEPPGRRECPFPSWRFPGLLLAAMVLLLYSFSGETCPYIRDPLNGQAVPANGTYEFGYQMHFICNEGYYL
IGEEILYCELKGSVAIWSGKPPICEKVLCTPPPKIKNGKHTFSEVEVFEYLDAVTYSCDPAPGPDPFSLI
GESTIYCGDNSVWSRAAPECKVVKCRFPVVENGKQISGFGKKFYYKATVMFECDKGFYLDGSDTIVCDSN
STWDPPVPKCLKVLPPSSTKPPALSHSVSTSSTTKSPASSASGPRPTYKPPVSNYPGYPKPEEGILDSLD
VWVIAVIVIAIVVGVAVICVVPYRYLQRRKKKGKADGGAEYATYQTKSTTPAEQRG
```

*Solvent accessibility predicted by WESA*

| S.No | Coord. residue | 9mer motif | Net charge | Comment | Predicted $K_D$ (µM) |
|---|---|---|---|---|---|
| 1 | Y184 | GKKFYYKAT | +3 | | 6.0 |
| 2 | Y185 | KKFYYKATV | +3 | | 5.09 |

### 25. sp|P08174|DAF_HUMAN Complement decay-accelerating factor OS=Homo sapiens OX=9606 GN=CD55 PE=1 SV=4

```
MTVARPSVPAALPLLGELPRLLLLVLLCLPAVWGDCGLPPDVPNAQPALEGRTSFPEDTVITYKCEESFV
KIPGEKDSVICLKGSQWSDIEEFCNRSCEVPTRLNSASLKQPYITQNYFPVGTVVEYECRPGYRREPSLS
PKLTCLQNLKWSTAVEFCKKKSCPNPGEIRNGQIDVPGGILFGATISFSCNTGYKLFGSTSSFCLISGSS
VQWSDPLPECREIYCPAPPQIDNGIIQGERDHYGYRQSVTYACNKGFTMIGEHSIYCTVNNDEGEWSGPP
PECRGKSLTSKVPPTVQKPTTVNVPTTEVSPTSQKTTTKTTTPNAQATRSTPVSRTTKHFHETTPNKGSG
TTSGTTRLLSGHTCFTLTGLLGTLVTMGLLT
```

*Solvent accessibility predicted by WESA*

| S.No | Coord. residue | 9mer motif | Net charge | Comment | Predicted $K_D$ (µM) |
|---|---|---|---|---|---|
| 1 | Y113 | LKQPYITQN | +1 | | 3.14 |
| 2 | H242 | GERDHYGYR | +1 | | 3.94 |
| 3 | Y245 | DHYGYRQSV | +1 | | 4.17 |
| 4 | H341 | TKHFHETTP | +2 | | 1.15 |

### 26. sp|P08174-2|DAF_HUMAN Isoform 1 of Complement decay-accelerating factor OS=Homo sapiens OX=9606 GN=CD55

```
MTVARPSVPAALPLLGELPRLLLLVLLCLPAVWGDCGLPPDVPNAQPALEGRTSFPEDTVITYKCEESFV
KIPGEKDSVICLKGSQWSDIEEFCNRSCEVPTRLNSASLKQPYITQNYFPVGTVVEYECRPGYRREPSLS
PKLTCLQNLKWSTAVEFCKKKSCPNPGEIRNGQIDVPGGILFGATISFSCNTGYKLFGSTSSFCLISGSS
VQWSDPLPECREIYCPAPPQIDNGIIQGERDHYGYRQSVTYACNKGFTMIGEHSIYCTVNNDEGEWSGPP
PECRGKSLTSKVPPTVQKPTTVNVPTTEVSPTSQKTTTKTTTPNAQATRSTPVSRTTKHFHETTPNKGSG
TTSGTTRLLSGSRPVTQAGMRWCDRSSLQSRTPGFKRSFHFSLPSSWYYRAHVFHVDRFAWDASNHGLAD
LAKEELRRKYTQVYRLFLVS
```

*Solvent accessibility predicted by WESA*

| S.No | Coord. residue | 9mer motif | Net charge | Comment | Predicted $K_D$ (µM) |
|---|---|---|---|---|---|
| 1 | Y113 | LKQPYITQN | +1 | | 6.23 |
| 2 | H242 | GERDHYGYR | +1 | | 2.64 |
| 3 | Y245 | DHYGYRQSV | +1 | | 2.86 |
| 4 | H339 | RTTKHFHET | +3 | | 0.29 |
| 5 | H341 | TKHFHETTP | +2 | | 0.42 |
| 6 | H405 | AHVFHVDRF | +2 | | 1.99 |

### 27. sp|P08174-3|DAF_HUMAN Isoform 3 of Complement decay-accelerating factor OS=Homo sapiens OX=9606 GN=CD55

```
MTVARPSVPAALPLLGELPRLLLLVLLCLPAVWGDCGLPPDVPNAQPALEGRTSFPEDTVITYKCEESFV
KIPGEKDSVICLKGSQWSDIEEFCNRSCEVPTRLNSASLKQPYITQNYFPVGTVVEYECRPGYRREPSLS
PKLTCLQNLKWSTAVEFCKKKSCPNPGEIRNGQIDVPGGILFGATISFSCNTGYKLFGSTSSFCLISGSS
VQWSDPLPECREIYCPAPPQIDNGIIQGERDHYGYRQSVTYACNKGFTMIGEHSIYCTVNNDEGEWSGPP
PECRGKSLTSKVPPTVQKPTTVNVPTTEVSPTSQKTTTKTTTPNAQATRSTPVSRTTKHFHETTPNKGSG
TTSGTTRLLSALQVRPFEVSGSSHISSKKMMCIL
```

*Solvent accessibility predicted by WESA*

Solvent accessibility predicted by WESA

| S.No | Coord. residue | 9mer motif | Net charge | Comment | Predicted $K_D$ (μM) |
|---|---|---|---|---|---|
| 1 | Y113 | LKQPYITQN | +1 | | 7.03 |
| 2 | H242 | GERDHYGYR | +1 | | 0.6 |
| 3 | Y245 | DHYGYRQSV | +1 | | 0.99 |
| 4 | H341 | TKHFHETTP | +2 | | 2.74 |
| 5 | H374 | SGSSHISSK | +2 | | 4.32 |

## 28. sp|P08174-4|DAF_HUMAN Isoform 4 of Complement decay-accelerating factor OS=Homo sapiens OX=9606 GN=CD55

```
MTVARPSVPAALPLLGELPRLLLLVLLCLPAVWGDCGLPPDVPNAQPALEGRTSFPEDTVITYKCEESFV
KIPGEKDSVICLKGSQWSDIEEFCNRSCEVPTRLNSASLKQPYITQNYFPVGTVVEYECRPGYRREPSLS
PKLTCLQNLKWSTAVEFCKKKSCPNPGEIRNGQIDVPGGILFGATISFSCNTGYKLFGSTSSFCLISGSS
VQWSDPLPECREIYCPAPPQIDNGIIQGERDHYGYRQSVTYACNKGFTMIGEHSIYCTVNNDEGEWSGPP
PECRGKSLTSKVPPTVQKPTTVNVPTTEVSPTSQKTTTKTTTPNAQATRSTPVSRTTKHFHETTPNKGSG
TTSGTTRLLSVLFM
```

*Solvent accessibility predicted by WESA*

| S.No | Coord. residue | 9mer motif | Net charge | Comment | Predicted $K_D$ (μM) |
|---|---|---|---|---|---|
| 1 | Y113 | LKQPYITQN | +1 | | 4.33 |
| 2 | H242 | GERDHYGYR | +1 | | 4.25 |
| 3 | Y245 | DHYGYRQSV | +1 | | 6.45 |
| 4 | H339 | RTTKHFHET | +3 | | 6.6 |
| 5 | H341 | TKHFHETTP | +2 | | 3.94 |

## 29. sp|P08174-5|DAF_HUMAN Isoform 5 of Complement decay-accelerating factor OS=Homo sapiens OX=9606 GN=CD55

```
MTVARPSVPAALPLLGELPRLLLLVLLCLPAVWGDCGLPPDVPNAQPALEGRTSFPEDTVITYKCEESFV
KIPGEKDSVICLKGSQWSDIEEFCNRSCEVPTRLNSASLKQPYITQNYFPVGTVVEYECRPGYRREPSLS
PKLTCLQNLKWSTAVEFCKKKSCPNPGEIRNGQIDVPGGILFGATISFSCNTGYKLFGSTSSFCLISGSS
VQWSDPLPECREIYCPAPPQIDNGIIQGERDHYGYRQSVTYACNKGFTMIGEHSIYCTVNNDEGEWSGPP
PECRGKSLTSKVPPTVQKPTTVNVPTTEVSPTSQKTTTKTTTPNAQATRSTPVSRTTKHFHETTPNKGSG
TTSGTTRLLSETVFHRVIQDGLDLLASRSACLGLPKCWDYRREPPHLARAHVFHVDRFAWDASNHGLADL
AKEELRRKYTQVYRLFLVS
```

*Solvent accessibility predicted by WESA*

| S.No | Coord. residue | 9mer motif | Net charge | Comment | Predicted $K_D$ (μM) |
|---|---|---|---|---|---|
| 1 | Y113 | LKQPYITQN | +1 | | 9.51 |
| 2 | H242 | GERDHYGYR | +1 | | 6.91 |
| 3 | Y245 | DHYGYRQSV | +1 | | 1.87 |
| 4 | H339 | RTTKHFHET | +3 | | 8.41 |
| 5 | H341 | TKHFHETTP | +2 | | 9.02 |
| 6 | H365 | ETVFHRVIQ | +1 | | 10.75 |
| 7 | C381 | SRSACLGLP | +1 | | 3.32 |
| 8 | C387 | GLPKCWDYR | +1 | | 5.77 |
| 9 | Y390 | KCWDYRREP | +1 | Possible S-S bond | 6.54 |
| 10 | H396 | REPPHLARA | +2 | | 0.94 |
| 11 | H401 | LARAHVFHV | +3 | | 7.23 |
| 12 | H404 | AHVFHVDRF | +2 | | 12.06 |

## 30. sp|P08174-6|DAF_HUMAN Isoform 6 of Complement decay-accelerating factor OS=Homo sapiens OX=9606 GN=CD55

```
MTVARPSVPAALPLLGELPRLLLLVLLCLPAVWGDCGLPPDVPNAQPALEGRTSFPEDTVITYKCEESFV
KIPGEKDSVICLKGSQWSDIEEFCNRSCEVPTRLNSASLKQPYITQNYFPVGTVVEYECRPGYRREPSLS
PKLTCLQNLKWSTAVEFCKKKSCPNPGEIRNGQIDVPGGILFGATISFSCNTGYKLFGSTSSFCLISGSS
VQWSDPLPECREIYCPAPPQIDNGIIQGERDHYGYRQSVTYACNKGFTMIGEHSIYCTVNNDEGEWSGPP
PECRGKSLTSKVPPTVQKPTTVNVPTTEVSPTSQKTTTKTTTPNAQGTETPSVLQKHTTENVSATRTPPT
PQKPTTVNVPATIVTPTPQKPTTINVPATGVSSTPQRHTIVNVSATGTLPTLQKPTRANDSATKSPAAAQ
TSFISKTLSTKTPSAAQNPMMTNASATQATLTAQKFTTAKVAFTQSPSAAPTRSTPVSRTTKHFHETTPN
KGSGTTSGTTRLLSGHTCFTLTGLLGTLVTMGLLT
```

*Solvent accessibility predicted by WESA*

| S.No | Coord. residue | 9mer motif | Net charge | Comment | Predicted $K_D$ (μM) |
|---|---|---|---|---|---|
| 1 | Y113 | LKQPYITQN | +1 | | 1.59 |
| 2 | H242 | GERDHYGYR | +1 | | 10.81 |
| 3 | H337 | VLQKHTTEN | +1 | | 14.5 |
| 4 | H388 | TPQRHTIVN | +2 | | 13.52 |
| 5 | H483 | RTTKHFHET | +3 | | 0.46 |
| 6 | H485 | TKHFHETTP | +2 | | 1.21 |

## 31. sp|P08174-7|DAF_HUMAN Isoform 7 of Complement decay-accelerating factor OS=Homo sapiens OX=9606 GN=CD55

```
MTVARPSVPAALPLLGELPRLLLLVLLCLPAVWGDCGLPPDVPNAQPALEGRTSFPEDTVITYKCEESFV
KIPGEKDSVICLKGSQWSDIEEFCNRSCEVPTRLNSASLKQPYITQNYFPVGTVVEYECRPGYRREPSLS
PKLTCLQNLKWSTAVEFCKKKSCPNPGEIRNGQIDVPGGILFGATISFSCNTGYKLFGSTSSFCLISGSS
VQWSDPLPECREIYCPAPPQIDNGIIQGERDHYGYRQSVTYACNKGFTMIGEHSIYCTVNNDEGEWSGPP
PECRGKSLTSKVPPTVQKPTTVNVPTTEVSPTSQKTTTKTTTPNAQGTETPSVLQKHTTENVSATRTPPT
PQKPTTVNVPATIVTPTPQKPTTINVPATGVSSTPQRHTIVNVSATGTLPTLQKPTRANDSATKSPAAAQ
TSFISKTLSTKTPSAAQNPMMTNASATQATLTAQKFTTAKVAFTQSPSAARKSTNVHSPVTNGLKSTQRF
PSAHITATRSTPVSRTTKHFHETTPNKGSGTTSGTTRLLSGHTCFTLTGLLGTLVTMGLLT
```

*Solvent accessibility predicted by WESA*

| S.No | Coord. residue | 9mer motif | Net charge | Comment | Predicted $K_D$ (μM) |
|---|---|---|---|---|---|
| 1 | Y113 | LKQPYITQN | +1 | | 8.59 |
| 2 | H242 | GERDHYGYR | +1 | | 4.96 |
| 3 | H337 | VLQKHTTEN | +1 | | 1.75 |
| 4 | H388 | TPQRHTIVN | +2 | | 8.7 |
| 5 | H509 | RTTKHFHET | +3 | | 0.11 |
| 6 | H511 | TKHFHETTP | +2 | | 0.52 |

## 32. sp|P17927-E|CR1_HUMAN Complement receptor type 1 OS=Homo sapiens OX=9606 GN=CR1 PE=1 SV=3

```
MGASSPRSPEPVGPPAPGLPFCCGGSLLAVVVLLALPVAWGQCNAPEWLPFARPTNLTDEFEFPIGTYLN
YECRPGYSGRPFSIICLKNSVWTGAKDRCRRKSCRNPPDPVNGMVHVIKGIQFGSQIKYSCTKGYRLIGS
SSATCIISGDTVIWDNETPICDRIPCGLPPTITNGDFISTNRENFHYGSVVTYRCNPGSGGRKVFELVGE
PSIYCTSNDDQVGIWSGPAPQCIIPNKCTPPNVENGILVSDNRSLFSLNEVVEFRCQPGFVMKGPRRVKC
QALNKWEPELPSCSRVCQPPPDVLHAERTQRDKDNFSPGQEVFYSCEPGYDLRGAASMRCTPQGDWSPAA
PTCEVKSCDDFMGQLLNGRVLFPVNLQLGAKVDFVCDEGFQLKGSSASYCVLAGMESLWNSSVPVCEQIF
CPSPPVIPNGRHTGKPLEVFPFGKTVNYTCDPHPDRGTSFDLIGESTIRCTSDPQGNGVWSSPAPRCGIL
GHCQAPDHFLFAKLKTQTNASDFPIGTSLKYECRPEYYGRPFSITCLDNLVWSSPKDVCKRKSCKTPPDP
VNGMVHVITDIQVGSRINYSCTTGHRLIGHSSAECILSGNAAHWSTKPPICQRIPCGLPPTIANGDFIST
NRENFHYGSVVTYRCNPGSGGRKVFELVGEPSIYCTSNDDQVGIWSGPAPQCIIPNKCTPPNVENGILVS
DNRSLFSLNEVVEFRCQPGFVMKGPRRVKCQALNKWEPELPSCSRVCQPPPDVLHAERTQRDKDNFSPGQ
EVFYSCEPGYDLRGAASMRCTPQGDWSPAAPTCEVKSCDDFMGQLLNGRVLFPVNLQLGAKVDFVCDEGF
QLKGSSASYCVLAGMESLWNSSVPVCEQIFCPSPPVIPNGRHTGKPLEVFPFGKAVNYTCDPHPDRGTSF
DLIGESTIRCTSDPQGNGVWSSPAPRCGILGHCQAPDHFLFAKLKTQTNASDFPIGTSLKYECRPEYYGR
PFSITCLDNLVWSSPKDVCKRKSCKTPPDPVNGMVHVITDIQVGSRINYSCTTGHRLIGHSSAECILSGN
TAHWSTKPPICQRIPCGLPPTIANGDFISTNRENFHYGSVVTYRCNLGSRGRKVFELVGEPSIYCTSNDD
QVGIWSGPAPQCIIPNKCTPPNVENGILVSDNRSLFSLNEVVEFRCQPGFVMKGPRRVKCQALNKWEPEL
PSCSRVCQPPPEILHGEHTPSHQDNFSPGQEVFYSCEPGYDLRGAASLHCTPQGDWSPEAPRCAVKSCDD
FLGQLPHGRVLFPLNLQLGAKVSFVCDEGFRLKGSSVSHCVLVGMRSLWNNSVPVCEHIFCPNPPAILNG
RHTGTPSGDIPYGKEISYTCDPHPDRGMTFNLIGESTIRCTSDPHGNGVWSSPAPRCELSVRAGHCKTPE
QFPFASPTIPINDFEFPVGTSLNYECRPGYFGKMFSISCLENLVWSSVEDNCRRKSCGPPPEPFNGMVHI
NTDTQFGSTVNYSCNEGFRLIGSPSTTCLVSGNNVTWDKKAPICEIISCEPPPTISNGDFYSNNRTSFHN
GTVVTYQCHTGPDGEQLFELVGERSIYCTSKDDQVGVWSSPPPRCISTNKCTAPEVENAIRVPGNRSFFS
LTEIIRFRCQPGFVMVGSHTVQCQTNGRWGPKLPHCSRVCQPPPEILHGEHTLSHQDNFSPGQEVFYSCE
PSYDLRGAASLHCTPQGDWSPEAPRCTVKSCDDFLGQLPHGRVLLPLNLQLGAKVSFVCDEGFRLKGRSA
SHCVLAGMKALWNSSVPVCEQIFCPNPPAILNGRHTGTPFGDIPYGKEISYACDTHPDRGMTFNLIGESS
IRCTSDPQGNGVWSSPAPRCELSVPAACPHPPKIQNGHYIGGHVSLYLPGMTISYICDPGYLLVGKGFIF
CTDQGIWSQLDHYCKEVNCSFPLFMNGISKELEMKKVYHYGDYVTLKCEDGYTLEGSPWSQCQADDRWDP
PLAKCTSRTHD
```

*Solvent accessibility predicted by WESA*

| S.No | Coord. residue | 9mer motif | Net charge | Comment | Predicted $K_D$ (μM) |
|---|---|---|---|---|---|
| 1 | H186 | RENFHYGSV | +1 | | 14.93 |
| 2 | C297 | CSRVCQPPP | +1 | Possible S-S bond | 11.98 |
| 3 | Y528 | RPEYYGRPF | +1 | | 11.12 |
| 4 | H590 | RLIGHSSAE | +1 | | 14.21 |
| 5 | C616 | QRIPCGLPP | +1 | | 9.44 |
| 6 | H636 | RENFHYGSV | +1 | | 1.17 |
| 7 | C747 | CSRVCQPPP | +1 | Possible S-S bond | 2.25 |
| 8 | Y978 | RPEYYGRPF | +1 | | 5.89 |
| 9 | H1040 | RLIGHSSAE | +1 | | 4.87 |
| 10 | H1053 | GNTAHWSTK | +2 | | 7.52 |
| 11 | C1061 | KPPICQRIP | +2 | | 8.66 |
| 12 | H1086 | RENFHYGSV | +1 | | 5.69 |
| 13 | C1132 | PAPQCIIPN | 0(CYS motif) | | 1.0 |
| 14 | C1197 | CSRVCQPPP | +1 | Possible S-S bond | 4.69 |
| 15 | H1212 | HTPSHQDNF | +1 | | 4.99 |
| 16 | C1253 | EAPRCAVKS | +1 | | 8.09 |
| 17 | H1267 | GQLPHGRVL | +2 | | 1.4 |
| 18 | C1316 | SVPVCEHIF | 0(CYS motif) | | 3.46 |
| 19 | H1395 | VRAGHCKTP | +3 | Possible S-S bond | 2.93 |
| 20 | H1539 | RTSFHNGTV | +2 | | 0.09 |
| 21 | C1585 | PPPRCISTN | +1 | | 1.24 |
| 22 | H1645 | PKLPHCSRV | +3 | Possible S-S bond | 7.46 |
| 23 | C1650 | CSRVCQPPP | +1 | Possible S-S bond | 2.86 |
| 24 | H1665 | HTLSHQDNF | +1 | | 1.36 |
| 25 | C1706 | EAPRCTVKS | +1 | | 9.31 |
| 26 | H1720 | GQLPHGRVL | +2 | | 0.61 |
| 27 | H1850 | AACPHPPKI | +2 | Possible S-S bond | 6.28 |
| 28 | H1929 | KKVYHYGDY | +2 | | 1.86 |
| 29 | Y1933 | HYGDYVTLK | +1 | | 1.78 |
| 30 | H1970 | TSRTHD___ | +1 | | 0.97 |

## 33. sp|P17927-C|CR1_HUMAN Complement receptor type 1 OS=Homo sapiens OX=9606 GN=CR1 PE=1 SV=3

```
KHRKGNNAHENPKEVAIHLHSQGGSSVHPRTLQTNEENSRVLP
```

*Solvent accessibility predicted by WESA*

| S.No | Coord. residue | 9mer motif | Net charge | Comment | Predicted $K_D$ (μM) |
|---|---|---|---|---|---|
| 1 | H2 | ___KHRKGN | +4 | | 1.33 |
| 2 | H9 | GNNAHENPK | +1 | | 0.42 |
| 3 | H18 | EVAIHLHSQ | +1 | | 0.29 |
| 4 | H20 | AIHLHSQGG | +2 | | 0.73 |
| 5 | H28 | GSSVHPRTL | +2 | | 18.58 |

## 34. sp|Q03591|FHR1_HUMAN Complement factor H-related protein 1 OS=Homo sapiens OX=9606 GN=CFHR1 PE=1 SV=2

```
MWLLVSVILISRISSVGGEATFCDFPKINHGILYDEEKYKPFSQVPTGEVFYYSCEYNFVSPSKSFWTRI
TCTEEGWSPTPKCLRLCFFPFVENGHSESSGQTHLEGDTVQIICNTGYRLQNNENNISCVERGWSTPPKC
RSTDTSCVNPPTVQNAHILSRQMSKYPSGERVRYECRSPYEMFGDEEVMCLNGNWTEPPQCKDSTGKCGP
PPPIDNGDITSFPLSVYAPASSVEYQCQNLYQLEGNKRITCRNGQWSEPPKCLHPCVISREIMENYNIAL
RWTAKQKLYLRTGESAEFVCKRGYRLSSRSHTLRTTCWDGKLEYPTCAKR
```

*Solvent accessibility predicted by WESA*

| S.No | Coord. residue | 9mer motif | Net charge | Comment | Predicted $K_D$ (μM) |
|---|---|---|---|---|---|
| 1 | H30 | PKINHGILY | +2 | | 0.73 |
| 2 | C87 | CLRLCFFPF | +1 | Possible S-S bond | 1.32 |
| 3 | H157 | VQNAHILSR | +2 | | 2.94 |
| 4 | C208 | STGKCGPPP | +1 | | 3.58 |
| 5 | H264 | PKCLHPCVI | +2 | Possible S-S bond | 9.21 |
| 6 | C266 | CLHPCVISR | +2 | Possible S-S bond | 4.01 |
| 7 | Y289 | KQKLYLRTG | +3 | | 13.25 |
| 8 | H311 | SSRSHTLRT | +3 | | 19.49 |
| 9 | C317 | LRTTCWDGK | +1 | | 10.37 |

## 35. sp|P36980|FHR2_HUMAN Complement factor H-related protein 2 OS=Homo sapiens OX=9606 GN=CFHR2 PE=1 SV=1

```
MWLLVSVILISRISSVGGEAMFCDFPKINHGILYDEEKYKPFSQVPTGEVFYYSCEYNFVSPSKSFWTRI
TCAEEGWSPTPKCLRLCFFPFVENGHSESSGQTHLEGDTVQIICNTGYRLQNNENNISCVERGWSTPPKC
RSTISAEKCGPPPPIDNGDITSFLLSVYAPGSSVEYQCQNLYQLEGNNQITCRNGQWSEPPKCLDPCVIS
QEIMEKYNIKLKWTNQQKLYSRTGDIVEFVCKSGYHPTKSHSFRAMCQNGKLVYPSCEEK
```

*Solvent accessibility predicted by WESA*

| S.No | Coord. residue | 9mer motif | Net charge | Comment | Predicted $K_D$ (μM) |
|---|---|---|---|---|---|
| 1 | H30 | PKINHGILY | +2 | | 9.64 |
| 2 | C87 | CLRLCFFPF | +1 | Possible S-S bond | 0.51 |
| 3 | Y217 | IMEKYNIKL | +1 | | 0.74 |
| 4 | Y230 | QQKLYSRTG | +2 | | 7.54 |
| 5 | H246 | KSGYHPTKS | +3 | | 1.67 |
| 6 | H251 | PTKSHSFRA | +3 | | 2.85 |

## 36. sp|P36980-2|FHR2_HUMAN Isoform Short of Complement factor H-related protein 2 OS=Homo sapiens OX=9606 GN=CFHR2

```
MWLLVSVILISRISSVGGEAMFCDFPKINHGILYDEEKYKPFSQVPTGEVFYYSCEYNFVSPSKSFWTRI
TCAEEGWSPTPKCLRLCFFPFVENGHSESSGQTHLEGDTVQIICNTGYRLQNNENNISCVERGWSTPPKC
RSTSSSVEYQCQNLYQLEGNNQITCRNGQWSEPPKCLDPCVISQEIMEKYNIKLKWTNQQKLYSRTGDIV
EFVCKSGYHPTKSHSFRAMCQNGKLVYPSCEEK
```

*Solvent accessibility predicted by WESA*

| S.No | Coord. residue | 9mer motif | Net charge | Comment | Predicted $K_D$ (μM) |
|---|---|---|---|---|---|
| 1 | H30 | PKINHGILY | +2 | | 5.47 |
| 2 | Y118 | CNTGYRLQN | +1 | Possible S-S bond | 11.73 |
| 3 | Y190 | IMEKYNIKL | +1 | | 3.87 |
| 4 | Y203 | QQKLYSRTG | +2 | | 9.68 |
| 5 | H219 | KSGYHPTKS | +3 | | 1.99 |
| 6 | H224 | PTKSHSFRA | +3 | | 2.94 |

## 37. sp|Q02985|FHR3_HUMAN Complement factor H-related protein 3 OS=Homo sapiens OX=9606 GN=CFHR3 PE=1 SV=2

```
MLLLINVILTLWVSCANGQVKPCDFPDIKHGGLFHENMRRPYFPVAVGKYYSYYCDEHFETPSGSYWDYI
HCTQNGWSPAVPCLRKCYFPYLENGYNQNYGRKFVQGNSTEVACHPGYGLPKAQTTVTCTEKGWSPTPRC
IRVRTCSKSDIEIENGFISESSSIYILNKEIQYKCKPGYATADGNSSGSITCLQNGWSAQPICINSSEKC
GPPPPISNGDTTSFLLKVYVPQSRVEYQCQPYYELQGSNYVTCSNGEWSEPPRCIHPCIITEENMNKNNI
KLKGRSDRKYYAKTGDTIEFMCKLGYNANTSILSFQAVCREGIVEYPRCE
```

*Solvent accessibility predicted by WESA*

| S.No | Coord. residue | 9mer motif | Net charge | Comment | Predicted $K_D$ (μM) |
|---|---|---|---|---|---|
| 1 | H30 | PDIKHGGLF | +1 | | 8.26 |
| 2 | Y42 | MRRPYFPVA | +2 | | 0.29 |
| 3 | Y50 | AVGKYYSYY | +1 | | 7.1 |
| 4 | Y88 | LRKCYFPYL | +2 | Possible S-S bond | 5.63 |
| 5 | Y100 | YNQNYGRKF | +2 | | 2.38 |
| 6 | C140 | PTPRCIRVR | +3 | | 3.41 |
| 7 | C264 | EPPRCIHPC | +1 | Possible S-S bond | 3.7 |
| 8 | H266 | PRCIHPCII | +2 | Possible S-S bond | 9.5 |
| 9 | C268 | CIHPCIITE | 0(CYS motif) | Possible S-S bond | 14.75 |
| 10 | Y290 | SDRKYYAKT | +2 | | 0.41 |
| 11 | Y291 | DRKYYAKTG | +2 | | 6.57 |
| 12 | C319 | FQAVCREGI | 0(CYS motif) | | 0.4 |

## 38. sp|Q02985-2|FHR3_HUMAN Isoform 2 of Complement factor H-related protein 3 OS=Homo sapiens OX=9606 GN=CFHR3

```
MLLLINVILTLWVSCANGQVKPCDFPDIKHGGLFHENMRRPYFPVAVGKYYSYYCDEHFETPSGSYWDYI
HCTQNGWSPAVPCLRKCYFPYLENGYNQNYGRKFVQGNSTEVACHPGYGLPKAQTTVTCTEKGWSPTPRC
IRVNSSEKCGPPPPISNGDTTSFLLKVYVPQSRVEYQCQPYYELQGSNYVTCSNGEWSEPPRCIHPCIIT
EENMNKNNIKLKGRSDRKYYAKTGDTIEFMCKLGYNANTSILSFQAVCREGIVEYPRCE
```

*Solvent accessibility predicted by WESA*

| S.No | Coord. residue | 9mer motif | Net charge | Comment | Predicted $K_D$ (μM) |
|---|---|---|---|---|---|
| 1 | H30 | PDIKHGGLF | +1 | | 1.96 |
| 2 | Y42 | MRRPYFPVA | +2 | | 7.76 |
| 3 | Y50 | AVGKYYSYY | +1 | | 8.91 |
| 4 | Y88 | LRKCYFPYL | +2 | Possible S-S bond | 4.95 |
| 5 | Y100 | YNQNYGRKF | +2 | | 6.03 |
| 6 | C203 | EPPRCIHPC | +1 | Possible S-S bond | 7.43 |
| 7 | H205 | PRCIHPCII | +2 | Possible S-S bond | 16.61 |
| 8 | Y229 | SDRKYYAKT | +2 | | 1.87 |
| 9 | Y230 | DRKYYAKTG | +2 | | 2.06 |
| 10 | C258 | FQAVCREGI | 0(CYS motif) | | 11.22 |

## 39. sp|Q92496|FHR4_HUMAN Complement factor H-related protein 4 OS=Homo sapiens OX=9606 GN=CFHR4 PE=1 SV=3

```
MLLLINVILTLWVSCANGQEVKPCDFPEIQHGGLYYKSLRRLYFPAAAGQSYSYYCDQNFVTPSGSYWDY
IHCTQDGWSPTVPCLRTCSKSDVEIENGFISESSSIYILNEETQYNCKPGYATAEGNSSGSITCLQNGWS
TQPICIKFCDMPVFENSRAKSNGMWFKLHDTLDYECYDGYESSYGNTTDSIVCGEDGWSHLPTCYNSSEN
CGPPPPISNGDTTSFPQKVYLPWSRVEYQCQSYYELQGSKYVTCSNGDWSEPPRCISMKPCEFPEIQHGH
LYYENTRRPYFPVATGQSYSYYCDQNFVTPSGSYWDYIHCTQDGWLPTVPCLRTCSKSDIEIENGFISES
SSIYILNKEIQYKCKPGYATADGNSSGSITCLQNGWSAQPICIKFCDMPVFENSRAKSNGMRFKLHDTLD
YECYDGYEISYGNTTGSIVCGEDGWSHFPTCYNSSEKCGPPPPISNGDTTSFLLKVYVPQSRVEYQCQSY
YELQGSNYVTCSNGEWSEPPRCIHPCIITEENMNKNNIQLKGKSDIKYYAKTGDTIEFMCKLGYNANTSV
LSFQAVCREGIVEYPRCE
```

*Solvent accessibility predicted by WESA*

| S.No | Coord. residue | 9mer motif | Net charge | Comment | Predicted $K_D$ (μM) |
|---|---|---|---|---|---|
| 1 | C15 | LWVSCANGQ | 0(CYS motif) | | 0.25 |
| 2 | Y43 | LRRLYFPAA | +2 | | 5.27 |
| 3 | C88 | CLRTCSKSD | +1 | Possible S-S bond | 3.76 |
| 4 | C204 | HLPTCYNSS | +1 | | 0.19 |
| 5 | Y251 | QGSKYVTCS | +1 | Possible S-S bond | 6.66 |
| 6 | H278 | PEIQHGHLY | +1 | | 2.87 |
| 7 | H280 | IQHGHLYYE | +1 | | 13.1 |
| 8 | Y290 | TRRPYFPVA | +2 | | 3.02 |
| 9 | C335 | CLRTCSKSD | +1 | Possible S-S bond | 9.46 |
| 10 | H416 | RFKLHDTLD | +1 | | 10.12 |
| 11 | C512 | EPPRCIHPC | +1 | Possible S-S bond | 1.5 |
| 12 | H514 | PRCIHPCII | +2 | Possible S-S bond | 0.97 |
| 13 | Y538 | SDIKYYAKT | +1 | | 9.4 |
| 14 | Y539 | DIKYYAKTG | +1 | | 12.91 |

## 40. sp|Q92496-2|FHR4_HUMAN Isoform 2 of Complement factor H-related protein 4 OS=Homo sapiens OX=9606 GN=CFHR4

```
MLLLINVILTLWVSCANGQVKPCDFPEIQHGGLYYKSLRRLYFPAAAGQSYSYYCDQNFVTPSGSYWDYI
HCTQDGWSPTVPCLRTCSKSDVEIENGFISESSSIYILNEETQYNCKPGYATAEGNSSGSITCLQNGWST
QPICIKFCDMPVFENSRAKSNGMWFKLHDTLDYECYDGYESSYGNTTDSIVCGEDGWSHLPTCYNSSENC
GPPPPISNGDTTSFPQKVYLPWSRVEYQCQSYYELQGSKYVTCSNGDWSEPPRCISMKPCEFPEIQHGHL
YYENTRRPYFPVATGQSYSYYCDQNFVTPSGSYWDYIHCTQDGWLPTVPCLRTCSKSDIEIENGFISESS
SIYILNKEIQYKCKPGYATADGNSSGSITCLQNGWSAQPICIKFCDMPVFENSRAKSNGMRFKLHDTLDY
ECYDGYEISYGNTTGSIVCGEDGWSHFPTCYNSSEKCGPPPPISNGDTTSFLLKVYVPQSRVEYQCQSYY
ELQGSNYVTCSNGEWSEPPRCIHPCIITEENMNKNNIQLKGKSDIKYYAKTGDTIEFMCKLGYNANTSVL
SFQAVCREGIVEYPRCE
```

*Solvent accessibility predicted by WESA*

| S.No | Coord. residue | 9mer motif | Net charge | Comment | Predicted $K_D$ (μM) |
|---|---|---|---|---|---|
| 1 | Y34 | HGGLYYKSL | +2 | | 1.23 |
| 2 | Y42 | LRRLYFPAA | +2 | | 0.16 |
| 3 | C87 | CLRTCSKSD | +1 | Possible S-S bond | 0.1 |
| 4 | Y250 | QGSKYVTCS | +1 | Possible S-S bond | 5.12 |
| 5 | H277 | PEIQHGHLY | +1 | | 2.98 |
| 6 | H279 | IQHGHLYYE | +1 | | 2.7 |
| 7 | Y289 | TRRPYFPVA | +2 | | 13.13 |
| 8 | C330 | PTVPCLRTC | +1 | Possible S-S bond | 3.02 |
| 9 | C334 | CLRTCSKSD | +1 | Possible S-S bond | 13.9 |
| 10 | H415 | RFKLHDTLD | +1 | | 4.68 |
| 11 | C457 | SSEKCGPPP | 0(CYS motif) | | 2.81 |
| 12 | C511 | EPPRCIHPC | +1 | Possible S-S bond | 1.29 |
| 13 | H513 | PRCIHPCII | +2 | Possible S-S bond | 8.55 |
| 14 | Y537 | SDIKYYAKT | +1 | | 0.45 |
| 15 | Y538 | DIKYYAKTG | +1 | | 2.34 |

## 41. sp|Q92496-3|FHR4_HUMAN Isoform 3 of Complement factor H-related protein 4 OS=Homo sapiens OX=9606 GN=CFHR4

```
MLLLINVILTLWVSCANGQEVKPCDFPEIQHGGLYYKSLRRLYFPAAAGQSYSYYCDQNFVTPSGSYWDY
IHCTQDGWSPTVPCLRTCSKSDIEIENGFISESSSIYILNKEIQYKCKPGYATADGNSSGSITCLQNGWS
AQPICIKFCDMPVFENSRAKSNGMRFKLHDTLDYECYDGYEISYGNTTGSIVCGEDGWSHFPTCYNSSEK
CGPPPPISNGDTTSFLLKVYVPQSRVEYQCQSYYELQGSNYVTCSNGEWSEPPRCIHPCIITEENMNKNN
IQLKGKSDIKYYAKTGDTIEFMCKLGYNANTSVLSFQAVCREGIVEYPRCE
```

*Solvent accessibility predicted by WESA*

| S.No | Coord. residue | 9mer motif | Net charge | Comment | Predicted $K_D$ (μM) |
|---|---|---|---|---|---|
| 1 | C15 | LWVSCANGQ | 0(CYS motif) | | 7.4 |
| 2 | Y35 | HGGLYYKSL | +2 | | 3.61 |
| 3 | Y36 | GGLYYKSLR | +2 | | 11.74 |
| 4 | Y43 | LRRLYFPAA | +2 | | 0.74 |
| 5 | C88 | CLRTCSKSD | +1 | Possible S-S bond | 0.21 |
| 6 | H169 | RFKLHDTLD | +1 | | 4.8 |
| 7 | H267 | PRCIHPCII | +2 | Possible S-S bond | 4.42 |
| 8 | Y291 | SDIKYYAKT | +1 | | 2.6 |
| 9 | Y292 | DIKYYAKTG | +1 | | 10.65 |
| 10 | C320 | FQAVCREGI | 0(CYS motif) | | 2.51 |

## 42. sp|Q9BXR6|FHR5_HUMAN Complement factor H-related protein 5 OS=Homo sapiens OX=9606 GN=CFHR5 PE=1 SV=1

```
MLLLFSVILISWVSTVGGEGTLCDFPKIHHGFLYDEEDYNPFSQVPTGEVFYYSCEYNFVSPSKSFWTRI
TCTEEGWSPTPKCLRMCSFPFVKNGHSESSGLIHLEGDTVQIICNTGYSLQNNEKNISCVERGWSTPPIC
SFTKGECHVPILEANVDAQPKKESYKVGDVLKFSCRKNLIRVGSDSVQCYQFGWSPNFPTCKGQVRSCGP
PPQLSNGEVKEIRKEEYGHNEVVEYDCNPNFIINGPKKIQCVDGEWTTLPTCVEQVKTCGYIPELEYGYV
QPSVPPYQHGVSVEVNCRNEYAMIGNNMITCINGIWTELPMCVATHQLKRCKIAGVNIKTLLKLSGKEFN
HNSRIRYRCSDIFRYRHSVCINGKWNPEVDCTEKREQFCPPPPQIPNAQNMTTTVNYQDGEKVAVLCKEN
YLLPEAKEIVCKDGRWQSLPRCVESTAYCGPPPSINNGDTTSFPLSVYPPGSTVTYRCQSFYKLQGSVTV
TCRNKQWSEPPRCLDPCVVSEENMNKNNIQLKWRNDGKLYAKTGDAVEFQCKFPHKAMISSPPFRAICQE
GKFEYPICE
```

*Solvent accessibility predicted by WESA*

| S.No | Coord. residue | 9mer motif | Net charge | Comment | Predicted $K_D$ (μM) |
|---|---|---|---|---|---|
| 1 | H29 | FPKIHHGFL | +3 | | 8.89 |
| 2 | H30 | PKIHHGFLY | +3 | | 1.38 |
| 3 | H96 | VKNGHSESS | +1 | | 0.97 |
| 4 | C140 | TPPICSFTK | +1 | | 12.27 |
| 5 | H148 | KGECHVPIL | +1 | Possible S-S bond | 12.57 |
| 6 | H326 | CVATHQLKR | +3 | Possible S-S bond | 7.07 |
| 7 | H351 | KEFNHNSRI | +2 | | 18.23 |
| 8 | C442 | SLPRCVEST | 0(CYS motif) | | 6.09 |

## 43. sp|P04003|C4BPA_HUMAN C4b-binding protein alpha chain OS=Homo sapiens OX=9606 GN=C4BPA PE=1 SV=2

```
MHPPKTPSGALHRKRKMAAWPFSRLWKVSDPILFQMTLIAALLPAVLGNCGPPPTLSFAAPMDITLTETR
FKTGTTLKYTCLPGYVRSHSTQTLTCNSDGEWVYNTFCIYKRCRHPGELRNGQVEIKTDLSFGSQIEFSC
SEGFFLIGSTTSRCEVQDRGVGWSHPLPQCEIVKCKPPPDIRNGRHSGEENFYAYGFSVTYSCDPRFSLL
GHASISCTVENETIGVWRPSPPTCEKITCRKPDVSHGEMVSGFGPIYNYKDTIVFKCQKGFVLRGSSVIH
CDADSKWNPSPPACEPNSCINLPDIPHASWETYPRPTKEDVYVVGTVLRYRCHPGYKPTTDEPTTVICQK
NLRWTPYQGCEALCCPEPKLNNGEITQHRKSRPANHCVYFYGDEISFSCHETSRFSAICQGDGTWSPRTP
SCGDICNFPPKIAHGHYKQSSSYSFFKEEIIYECDKGYILVGQAKLSCSYSHWSAPAPQCKALCRKPELV
NGRLSVDKDQYVEPENVTIQCDSGYGVVGPQSITCSGNRTWYPEVPKCEWETPEGCEQVLTGKRLMQCLP
NPEDVKMALEVYKLSLEIEQLELQRDSARQSTLDKEL
```

*Solvent accessibility predicted by WESA*

| S.No | Coord. residue | 9mer motif | Net charge | Comment | Predicted $K_D$ (μM) |
|---|---|---|---|---|---|
| 1 | H2 | ___MHPPKT | +2 | | 3.48 |
| 2 | H89 | YVRSHSTQT | +2 | | 5.23 |
| 3 | Y110 | TFCIYKRCR | +3 | Possible S-S bond | 14.55 |
| 4 | H115 | KRCRHPGEL | +3 | Possible S-S bond | 3.8 |
| 5 | H333 | RYRCHPGYK | +4 | Possible S-S bond | 3.09 |
| 6 | H378 | EITQHRKSR | +3 | | 9.42 |
| 7 | H386 | RPANHCVYF | +2 | Possible S-S bond | 2.92 |
| 8 | C387 | PANHCVYFY | +1 | | 0.58 |
| 9 | H400 | SFSCHETSR | +1 | Possible S-S bond | 1.51 |
| 10 | H434 | PKIAHGHYK | +4 | | 6.04 |
| 11 | H436 | IAHGHYKQS | +3 | | 0.54 |
| 12 | Y443 | QSSSYSFFK | +1 | | 5.41 |
| 13 | Y470 | LSCSYSHWS | +1 | Possible S-S bond | 0.75 |

## 44. sp|P20851|C4BPB_HUMAN C4b-binding protein beta chain OS=Homo sapiens OX=9606 GN=C4BPB PE=1 SV=1

```
MFFWCACCLMVAWRVSASDAEHCPELPPVDNSIFVAKEVEGQILGTYVCIKGYHLVGKKTLFCNASKEWD
NTTTECRLGHCPDPVLVNGEFSSSGPVNVSDKITFMCNDHYILKGSNRSQCLEDHTWAPPFPICKSRDCD
PPGNPVHGYFEGNNFTLGSTISYYCEDRYYLVGVQEQQCVDGEWSSALPVCKLIQEAPKPECEKALLAFQ
ESKNLCEAMENFMQQLKESGMTMEELKYSLELKKAELKAKLL
```

*Solvent accessibility predicted by WESA*

| S.No | Coord. residue | 9mer motif | Net charge | Comment | Predicted $K_D$ (μM) |
|---|---|---|---|---|---|
| 1 | H54 | IKGYHLVGK | +3 | | 6.87 |
| 2 | H80 | CRLGHCPDP | +1 | Possible S-S bond | 4.37 |
| 3 | H110 | MCNDHYILK | +1 | Possible S-S bond | 8.02 |

## 45. sp|P20851-2|C4BPB_HUMAN Isoform 2 of C4b-binding protein beta chain OS=Homo sapiens OX=9606 GN=C4BPB

```
MFFWCACCLMVAWRVSASDEHCPELPPVDNSIFVAKEVEGQILGTYVCIKGYHLVGKKTLFCNASKEWDN
TTTECRLGHCPDPVLVNGEFSSSGPVNVSDKITFMCNDHYILKGSNRSQCLEDHTWAPPFPICKSRDCDP
PGNPVHGYFEGNNFTLGSTISYYCEDRYYLVGVQEQQCVDGEWSSALPVCKLIQEAPKPECEKALLAFQE
SKNLCEAMENFMQQLKESGMTMEELKYSLELKKAELKAKLL
```

*Solvent accessibility predicted by WESA*

| S.No | Coord. residue | 9mer motif | Net charge | Comment | Predicted $K_D$ (μM) |
|---|---|---|---|---|---|
| 1 | H53 | IKGYHLVGK | +3 | | 11.59 |
| 2 | H79 | CRLGHCPDP | +1 | Possible S-S bond | 11.19 |
| 3 | H109 | MCNDHYILK | +1 | Possible S-S bond | 2.57 |

**Species with HeMoQuest predicted HBMs**

>sp|P01024|CO3_HUMAN Complement C3 OS=Homo sapiens OX=9606 GN=C3 PE=1 SV=2
MGPTSGPSLLLLLLTHLPLALGSPMYSIITPNILRLESEETMVLEAHDAQGDVPVTVTVH
DFPGKKLVLSSEKTVLTPATNHMGNVTFTIPANREFKSEKGRNKFVTVQATFGTQVVEKV
VLVSLQSGYLFIQTDKTIYTPGSTVLYRI**FTVNHKLLP**VGRTVMVNIENPEGIPVKQDSL
SSQNQLGVLPLSWDIPELVNMGQW**KIRAYYENS**PQQVFSTEFEVKEYVLPSFEVIVEPTE
KFYYIYNEKGLEVTITARFLYGKKVEGTAFVIFGIQDGEQRISLPESLKRIPIEDGSGEV
VLSRKVLLDGVQNPRAEDLVGKSLYVSATVILHSGSDMVQAERSGIPIVTS**PYQIHFTKT**
PKYFKPGMPFDLMVFVTNPD**GSPAYRVPV**AVQGEDTVQSLTQGDGVAKL**SINTHPSQK**PL
SITVRTKKQELSEAEQATRTMQALPYSTVGNSNNYLHLSVLRTELRPGETLNVNFLLR**MD**
**RAHEAKI**RYYTYLIMNKGRLLKAGRQVREPGQDLVVLPLSITTDFIPSFRLVAYYTLIGA
SGQREVVADSVWVDVKDSCVGSLVVKSGQSEDRQPVPGQQMTLKIEGDHGARVVLVAVDK
GVFVLNKKNKLTQSKIWDVVEKADIGCTPGSGKDYAGVFSDAGLTFTSSSGQQTAQRAEL
QCPQPAARRRRSVQLTEKRMDKVGKYPKELRKCCEDGMRENPMRFSCQRRTRFISLGEAC
KKVFLDCCNYITE**LRRQHARASHLGLA**RSNLDEDIIAEENIVSRSEFPESWLWNVEDLKE
PPKNGISTKLMNIFLKDSITTWEILAVSMSDKKGICVADPFEVTVMQDFFIDLRLPYSVV
RNEQVEIRAVLYNYRQNQELKVRVELLH**NPAFCSLATTKRRHQQTV**TIPPKSSLSVPYVI
VPLKTGLQEVEV**KAAVYHHFISD**GVRKSLKVVPEGIRMNKTVAVRTLDPERLGREGVQKE
DIPPADLSDQVPDTESETRILLQGTPVAQMTEDAVDA**ERLKHLIVT**PSGCGEQNMIGMTP
TVIAVHYLDETEQWEKFGLEKRQGALELIKKGYTQQLAFRQPSSAFAAFVKRAPSTWLTA
YVVKVFSLAVNLIAID**SQVLCGAVK**WLILEKQKPDGVFQEDAPVIHQEMIGGLRNNNEKD
MALTAFVLISLQEAKDICEEQVNSLPGSITKAGDFLEANYMNLQRSYTVAIAGYALAQMG
RLKGPLLNKFLTTAKDKNRWEDPGKQLYNVEATSYALLALLQLKDFDFVPPVVRWLNEQR
YYGGGYGSTQATFMVFQALAQYQKDAPDHQELNLDVSLQLPSRSSKI**THRIHWESA**SLLR
SEETKENEGFTVTAEGKGQGTLSVVTMYHAKAKDQLTCNKFDLKVTIKPAPETEKRPQDA
KNTMILEICTRYRGDQDATMSILDISMMTGFAPDTDDLKQLANGVDRYISKYELDKAFSD
RNTLIIYLDKVSHSEDDCL**AFKVHQYFNVE**LIQPGAVKVYAYYNLEESC**TRFYHPEKE**DG
KLNKLCRDELCRCAEENCFIQKSDDKVTLEERLDKACEPGVDYVYKTRLVKVQLSNDFDE
YIMAIEQTIKSGSDEVQVGQQRTFISPIKCREALKL**EEKKHYLMW**GLSSDFWGEKPNLSY
IIGKDTWVEHWPEEDECQDEENQKQCQDLGAFTESMVVFGCPN

>sp|P00751|CFAB_HUMAN Complement factor B OS=Homo sapiens OX=9606 GN=CFB PE=1 SV=2
MGSNLSPQLCLMPFILGLLSGGVTTTPWSLARPQGSCSLEGVEIKGGSFRLLQEGQALEY
VCPSGFYPYPVQTRTCRSTGSWSTLKTQDQKTVRKAE**CRAIHCPRP**HDFENGEYW**PRSPY**
**YNVS**DEISFHCYDGYTLRGSANRTCQVNGRWSGQTAICDNGAGYCSNPGIPIGTRKVGSQ
YRLEDSVTYHCSRGLTLRGSQRRTCQEGGSWSGTEPSCQDSFMYDTPQEVAEAFLSSLTE
TIEGVDAEDGHGPGEQQKRKIVLDPSGSMNIYLVLDGSDSIGASNFTGAKKCLVNLIE**KV**
**ASYGVKP**RYGLV**TYATYPKIW**VKVSEADSSNADWVTKQLNEI**NYEDHKLKS**GTNTKKALQ
AVYSMMSWPDDVPPEGWNRTRHVIILMTDGLHNMGGDPITVIDEIRDLLYIGKDRKNPRE
DYLDVYVFGVGPLVNQVNINALASKKDNEQHVFKVKDMENLEDVFYQMIDESQSLSLCG**M**
**VWEHRKGTDYHKQPW**QAKISVIR**PSKGHESCM**GAVVSEYFVLTAAHCFTVDDKEHSIKVS
VGGEKRDLEIEVVLFHPNYNINGKKEAGIPEFYDYDVALIKLKNKLKYGQTIRPICLPCT
EGTTRALRLPPTTTCQQQKEELLPAQDIKALFVSEEEKKLT**RKEVYIKNG**DKKGSCERDA
Q**YAPGYDKVK**DISEVVTPRFLCTGGVSPYADPNTCRGDSGG**PLIVHKRSR**FIQVGVISWG
VVDVCKNQKRQK**QVPAHARDF**HINLFQVLPWLKEKLQDEDLGFL

>sp|P00751-2|CFAB_HUMAN Isoform 2 of Complement factor B OS=Homo sapiens OX=9606 GN=CFB
MGSNLSPQLCLMPFILGLLSGGVTTTPWSLARPQGSCSLEGVEIKGGSFRLLQEGQALEY
VCPSGFYPYPVQTRTCRSTGSWSTLKTQDQKTVRKAE**CRAIHCPRP**HDFENGEYW**PRSPY**
**YNVS**DEISFHCYDGYTLRGSANRTCQVNGRWSGQTAICDNGAGYCSNPGIPIGTRKVGSQ
YRLED**SVTYHCSRG**LTLRGSQRRTCQEGGSWSGTEPSCQDSFMYDTPQEVAEAFLSSLTE

TIEGVDAEDGHGPGEQQKRKIVLDPSGSMNIYLVLDGSDSIGASNFTGAKKCLVNLIE**KV**
**ASYGVKP**RYGLV**TYATYPKIW**VKVSEADSSNADWVTKQLNEI**NYEDHKLKS**GTNTKKALQ
AVYSMMSWPDDVPPEGWNRTRHVIILMTDGLHNMGGDPITVIDEIRDLLYIGKDRKNPRE
DYLDVYVFGVGPLVNQVNINALASKKDNEQHVFKVKDMENLEDVFYQMIDESQSLSLCG**M**
**VWEHRKGTDYHKQPW**QAKISVIR**PSKGHESCM**GAVVSEYFVLTAAHCFTVDDKEHSIKVS
VGKDATEGPGLHLCSPGNTSHFLQI**LHSTHPQCS**PIPCTPDQSGMGEDVKLGMTRGQRQE
AAHKEVVPTLLLQEGRSGTWR

>sp|P05156|CFAI_HUMAN Complement factor I OS=Homo sapiens OX=9606 GN=CFI PE=1 SV=2
MKLLHVFLLFLCFHLRFCKVTYTSQEDLVEKKCLAKKYTHLSCDKVFCQPWQRCIEGTCV
CKLPYQCPKNGTAVCATNRRSFPTYCQQKS**LECLHPGTK**FLNNGTCTAEGKFS**VSLKHGN**
**TD**SEGIVEVKLVDQDKTMFICKSSWSMREANVACLDLGFQQGADTQRRFKLSDLSINS**TE**
**CLHVHCRGL**ETSLAECTFTKRRTMGYQDFADVVCYTQKADSPMDDFFQCVNGKYISQMKA
CDGINDCGDQSDELCCKACQ**GKGFHCKSG**VCIPSQYQCNGEVDCITGEDEVGCAGFASVT
QEETEILTADMDAERRRIKSLLPKLSCGV**KNRMHIRRK**RIVGGKRAQLGDLPWQVAIKDA
SGITCGGI**YIGGCWILT**AAHCLR**ASKTHRYQI**WTTVVDWIHPDLKRIVIEYVDRIIFHEN
YNAGTYQNDIALIEMKKDG**NKKDCELPR**SIPACVPWSPYLFQPNDTCIVSGWGREKDNER
VFSLQWGEVKLISNCSKFYGNRFYEKEMECAGTYDGSIDACKGDSGGPLVCMDANNVTYV
WGVVSWGENCGKPEFPGVYTKVANYF**DWISYHVGRP**FISQYNV

>sp|P00746|CFAD_HUMAN Complement factor D OS=Homo sapiens OX=9606 GN=CFD PE=1 SV=5
**MHSWER**LAVLVLLGAAACAAPPRGRILGGREAEAHARPYMASVQLNGAHLCGGVLVAEQW
VLSAAHCLEDAADGKVQVLLGAHSLSQPEPSKRLYDVLRAVPHPDSQPDTIDHDLLLLQL
SEKATLGPAVRPLPWQRVDRDVAPGTLCDVAGW**GIVNHAGRR**PDSLQHVLLPVLDRATC**N**
**RRTHHDGAI**TERLMCAESNRRDSCKGDSGGPLVCGGVLEGVVTSGSRVCGNRKKPGIYTR
VASYAAWIDSVLA

>sp|P27918|PROP_HUMAN Properdin OS=Homo sapiens OX=9606 GN=CFP PE=1 SV=2
MITEGAQAPRLLLPPLLLLLTLPATGSDPVLCFTQYEESSGKCKGLLGGGVSVEDCCLN**T**
**AFAYQKRSGGLCQPCRSPR**WSLWSTWAPCSVTCSEG**SQLRYRRCV**G**WNGQCSGKV**APGTL
EWQLQACEDQQCCPEMGGWSGWGPWEPCSVTCSKGTRTRR**RACNHPAPKCGGHCPGQ**AQE
SEACD**TQQVCPTHG**AWATWG**PWTPCSASCHGGPHEPKE**TRSRKCSAPEPSQKPPGKPC**PG**
**LAYEQRRCTGLP**PCPVAGGWGPWGPVSPCPVTCGLGQTMEQ**RTCNHPVPQ**HGGPFCAGD**A**
**TRTHICNTAV**PCPVDGEWDSWGEWSPCIRRNMKSISCQEIPGQQSRGRTCRGR**KFDGHRC**
**AG**QQ**QDIRHCYSIQHCPLK**GSWSEWSTWGLCMPPCGPNPTRARQRLCTP**LLPKYPPTV**SM
VEGQGEKNVTFWGRPLPRCEELQGQKLVVEEK**RPCLHVPAC**KDPEEEEL

>sp|P08603|CFAH_HUMAN Complement factor H OS=Homo sapiens OX=9606 GN=CFH PE=1 SV=4
MRLLAKIICLMLWAICVAEDCNELPPRRNTEILTGSWSDQTYPEGTQAIYKCRPGYRSLG
NVIMVCRKGEWVALNPLRKCQK**RPCGHPGDT**PFGTFTLTGGNVFEYGVKAVYTCNEGYQL
LGEINYRECDTDGWTNDIPICEVVKCLPVTAPENGKIVSSAMEPDREYHFGQAVRFVCNS
GYKIEGDEEMHCSDDGFWSKEKPKCVEISCKSPDVINGSPISQKIIYKENERFQYKCNMG
YEYSERGDAVCTESGWRPLPSCEEKSCDNPYIPNGDYSPLRIKHRTGDEITYQC**RNGFYP**
**ATR**GNTAKCTSTGWI**PAPRCTLKP**CDY**PDIKHGGLYHENMRRPYFPVAVGKYYSYY**CDEH
FETPSGSYWDHIHCTQDGWSPAVP**CLRKCYFPYL**ENG**YNQNYGRKF**VQGKSIDVACHPGY
ALPKAQTTVTCMENGWSPTPRCI**RVKTCSKSS**IDIENGFISESQYTYALKEKAKYQCKLG
YVTADGETSGSITCGKDGWSAQPTCIKSCDIPVFMNARTKNDFTWFKLNDTLDYECHDGY
ESNTGSTTGSIVCGYNGWSDLPICYERECELPKIDVHLVPDRKKDQYKVGEVLKFSCKPG
FTIVGPNSVQCYHFGLSPDLPICKEQVQSCGPPPELLNGNVKEKTKEEYGHSEVVEYYCN
PRFLMKGPNKIQCVDGEWTTLPVCIVEESTCGDIPELEHGWAQLSSPPYYYGDSVEFNCS
ESF**TMIGHRSIT**CIHGVWTQLPQCVAID**KLKKCKSSN**L**IILEEHLKNK**KEFDHNSNIRYR
CRGKEGWIHTVCINGRWDPEVNCSMA**QIQLCPPPP**QIPNSHNMTTTLNYRDGEKVSVLCQ

ENYLIQEGEEITCKDGRWQ**SIPLCVEKI**PCSQPPQIEHGTINSSRSSQESYAHGTKLSYT
CEGGFRISEENETTCYMGKWSSPPQCEGLPCKSPPEISHGVVAHMSDSYQYGEEVTYKCF
EGFGIDGPAIAKCLG**EKWSHPPSCIKTDCLSLP**SFENAIPMGEKKDVYKAGEQVTYTCAT
YYKMDGASNVTCINSRWT**GRPTCRDTSCVNPP**T**VQNAYIVSR**QMSKYPSGERVRYQCRSP
YEMFGDEEVMCLNGNWTEPPQCKDSTGKCGPPPPIDNGDITSFPLSVYAPASSVEYQCQN
LYQLEGNKRITCRNGQWSEP**PKCLHPCVI**SREIMENYNIALRWTA**KQKLYSRTG**ESVEFV
CKRGYRL**SSRSHTLRTTCWDGK**LEYPTCAKR

>sp|P08603-2|CFAH_HUMAN Isoform 2 of Complement factor H OS=Homo sapiens OX=9606 GN=CFH
MRLLAKIICLMLWAICVAEDCNELPPRRNTEILTGSWSDQTYPEGTQAIYKCRPGYRSLG
NVIMVCRKGEWVALNPLRKCQK**RPCGHPGDT**PFGTFTLTGGNVFEYGVKAVYTCNEGYQL
LGEINYRECDTDGWTNDIPICEVVKCLPVTAPENGKIVSSAMEPDREYHFGQAVRFVCNS
GYKIEGDEEMHCSDDGFWSKEKPKCVEISCKSPDVINGSPISQKIIYKENERFQYKCNMG
YEYSERGDAVCTESGWRPLPSCEEKSCDNPYIPNGDYSPLRIKHRTGDEITYQC**RNGFYP**
**ATR**GNTAKCTSTGWI**PAPRCTLKP**CDY**PDIKHGGLY**HEN**MRRPYFPVAVGKYYSYY**CDEH
FETPSG**SYWDHIHCT**QDGWS**PAVPCLRKCYFPYL**ENG**YNQNYGRKF**VQGKSIDVACHPGY
ALPKAQTTVTCMENGWSPTPRCIRVSFTL

>sp|P15529|MCP_HUMAN Membrane cofactor protein OS=Homo sapiens OX=9606 GN=CD46 PE=1 SV=3
MEPPGRRECPFPSWRFPGLLLAAMVLLLYSFSDACEEPPTFEAMELIG**KPKPYYEIG**ERV
DYKCK**KGYFYIPPL**ATHTI**CDRNHTWLP**VSDDACYRETCPYIRDPLNGQAVPANGTYEFG
YQMHFICNEGYYLIGEEILYCELKGSVAIWSGKPPICEKVLCTPPPKIKNGKHTFSEVEV
FEYLDAVTYSCDPAPGPDPFSLIGESTIYCGDNSVWSRAAPECKVVKCRFPVVENGKQIS
GF**GKKFYYKAT**VMFECDKGFYLDGSDTIVCDSNSTWDPPVPKCLKVLPPSSTKPPALSHS
VSTSSTTKSPASSASG**PRPTYKPPV**SNYPGYPKPEEGILDSLDVWVIAVIVIAIVVGVAV
ICVVPYRYLQRRKKKGTYLTDETHREVKFTSL

>sp|P15529-2|MCP_HUMAN Isoform B of Membrane cofactor protein OS=Homo sapiens OX=9606 GN=CD46
MEPPGRRECPFPSWRFPGLLLAAMVLLLYSFSDACEEPPTFEAMELIG**KPKPYYEIG**ERV
DYKCK**KGYFYIPPL**ATHTI**CDRNHTWLP**VSDDACYRETCPYIRDPLNGQAVPANGTYEFG
YQMHFICNEGYYLIGEEILYCELKGSVAIWSGKPPICEKVLCTPPPKIKNGKHTFSEVEV
FEYLDAVTYSCDPAPGPDPFSLIGESTIYCGDNSVWSRAAPECKVVKCRFPVVENGKQIS
GF**GKKFYYKAT**VMFECDKGFYLDGSDTIVCDSNSTWDPPVPKCLKVLPPSSTKPPALSHS
VSTSSTTKSPASSASGPRPTYKPPVSNYPGYPKPEEGILDSLDVWVIAVIVIAIVVGVAV
ICVVPYRYLQRRKKKGKADGGAEYATYQTKSTTPAEQRG

>sp|P15529-11|MCP_HUMAN Isoform C of Membrane cofactor protein OS=Homo sapiens OX=9606 GN=CD46
MEPPGRRECPFPSWRFPGLLLAAMVLLLYSFSDACEEPPTFEAMELIG**KPKPYYEIG**ERV
DYKCK**KGYFYIPPL**ATHTI**CDRNHTWLP**VSDDACYRETCPYIRDPLNGQAVPANGTYEFG
YQMHFICNEGYYLIGEEILYCELKGSVAIWSGKPPICEKVLCTPPPKIKNGKHTFSEVEV
FEYLDAVTYSCDPAPGPDPFSLIGESTIYCGDNSVWSRAAPECKVVKCRFPVVENGKQIS
GF**GKKFYYKAT**VMFECDKGFYLDGSDTIVCDSNSTWDPPVPKCLKVSTSSTTKSPASSAS
G**PRPTYKPPV**SNYPGYPKPEEGILDSLDVWVIAVIVIAIVVGVAVICVVPYRYLQRRKKK
GTYLTDETHREVKFTSL

>sp|P15529-3|MCP_HUMAN Isoform D of Membrane cofactor protein OS=Homo sapiens OX=9606 GN=CD46
MEPPGRRECPFPSWRFPGLLLAAMVLLLYSFSDACEEPPTFEAMELIG**KPKPYYEIG**ERV
DYKCK**KGYFYIPPL**ATHTI**CDRNHTWLP**VSDDACYRETCPYIRDPLNGQAVPANGTYEFG
YQMHFICNEGYYLIGEEILYCELKGSVAIWSGKPPICEKVLCTPPPKIKNGKHTFSEVEV
FEYLDAVTYSCDPAPGPDPFSLIGESTIYCGDNSVWSRAAPECKVVKCRFPVVENGKQIS
GF**GKKFYYKAT**VMFECDKGFYLDGSDTIVCDSNSTWDPPVPKCLKVSTSSTTKSPASSAS

G**PRPTYKPPV**SNYPGYPKPEEGILDSLDVWVIAVIVIAIVVGVAVICVVPYRYLQRRKKK
GKADGGAEYATYQTKSTTPAEQRG

>sp|P15529-12|MCP_HUMAN Isoform E of Membrane cofactor protein OS=Homo sapiens OX=9606 GN=CD46
MEPPGRRECPFPSWRFPGLLLAAMVLLLYSFSDACEEPPTFEAMELIG**KPKPYYEIG**ERV
DYKCK**KGYFYIPPL**ATHTI**CDRNHTWLP**VSDDACYRETCPYIRDPLNGQAVPANGTYEFG
YQMHFICNEGYYLIGEEILYCELKGSVAIWSG**KPPICEKVL**CTPPPKIKNGKHTFSEVEV
FEYLDAVTYSCDPAPGPDPFSLIGESTIYCGDNSVWSRAAPECKVVKCRFPVVENGKQIS
GF**GKKFYYKAT**VMFECDKGFYLDGSDTIVCDSNSTWDPPVPKCLKG**PRPTYKPPV**SNYPG
YPKPEEGILDSLDVWVIAVIVIAIVVGVAVICVVPYRYLQRRKKKGTYLTDETHREVKFT
SL

>sp|P15529-4|MCP_HUMAN Isoform F of Membrane cofactor protein OS=Homo sapiens OX=9606 GN=CD46
MEPPGRRECPFPSWRFPGLLLAAMVLLLYSFSDACEEPPTFEAMELIG**KPKPYYEIG**ERV
DYKCK**KGYFYIPPL**ATHTI**CDRNHTWLP**VSDDACYRETCPYIRDPLNGQAVPANGTYEFG
YQMHFICNEGYYLIGEEILYCELKGSVAIWSG**KPPICEKVL**CTPPPKIKNGKHTFSEVEV
FEYLDAVTYSCDPAPGPDPFSLIGESTIYCGDNSVWSRAAPECKVVKCRFPVVENGKQIS
GF**GKKFYYKAT**VMFECDKGFYLDGSDTIVCDSNSTWDPPVPKCLKG**PRPTYKPPV**SNYPG
YPKPEEGILDSLDVWVIAVIVIAIVVGVAVICVVPYRYLQRRKKKGKADGGAEYATYQTK
STTPAEQRG

>sp|P15529-13|MCP_HUMAN Isoform G of Membrane cofactor protein OS=Homo sapiens OX=9606 GN=CD46
MEPPGRRECPFPSWRFPGLLLAAMVLLLYSFSDACEEPPTFEAMELIG**KPKPYYEIG**ERV
DYKCK**KGYFYIPPL**ATHTI**CDRNHTWLP**VSDDACYRETCPYIRDPLNGQAVPANGTYEFG
YQMHFICNEGYYLIGEEILYCELKGSVAIWSGKPPICEKVLCTPPPKIKNGKHTFSEVEV
FEYLDAVTYSCDPAPGPDPFSLIGESTIYCGDNSVWSRAAPECKVVKCRFPVVENGKQIS
GF**GKKFYYKAT**VMFECDKGFYLDGSDTIVCDSNSTWDPPVPKCLKVLPPSSTKPPALSHS
VSTSSTTKSPASSASGYPKPEEGILDSLDVWVIAVIVIAIVVGVAVICVVPYRYLQRRKK
KGTYLTDETHREVKFTSL

>sp|P15529-5|MCP_HUMAN Isoform H of Membrane cofactor protein OS=Homo sapiens OX=9606 GN=CD46
MEPPGRRECPFPSWRFPGLLLAAMVLLLYSFSDACEEPPTFEAMELIG**KPKPYYEIG**ERV
DYKCK**KGYFYIPPL**ATHTI**CDRNHTWLP**VSDDACYRETCPYIRDPLNGQAVPANGTYEFG
YQMHFICNEGYYLIGEEILYCELKGSVAIWSGKPPICEKVLCTPPPKIKNGKHTFSEVEV
FEYLDAVTYSCDPAPGPDPFSLIGESTIYCGDNSVWSRAAPECKVVKCRFPVVENGKQIS
GF**GKKFYYKATV**MFECDKGFYLDGSDTIVCDSNSTWDPPVPKCLKVLPPSSTKPPALSHS
VSTSSTTKSPASSASGYPKPEEGILDSLDVWVIAVIVIAIVVGVAVICVVPYRYLQRRKK
KGKADGGAEYATYQTKSTTPAEQRG

>sp|P15529-14|MCP_HUMAN Isoform I of Membrane cofactor protein OS=Homo sapiens OX=9606 GN=CD46
MEPPGRRECPFPSWRFPGLLLAAMVLLLYSFSDACEEPPTFEAMELIG**KPKPYYEIG**ERV
DYKCK**KGYFYIPPL**ATHTI**CDRNHTWLP**VSDDACYRETCPYIRDPLNGQAVPANGTYEFG
YQMHFICNEGYYLIGEEILYCELKGSVAIWSGKPPICEKVLCTPPPKIKNGKHTFSEVEV
FEYLDAVTYSCDPAPGPDPFSLIGESTIYCGDNSVWSRAAPECKVVKCRFPVVENGKQIS
GF**GKKFYYKAT**VMFECDKGFYLDGSDTIVCDSNSTWDPPVPKCLKVSTSSTTKSPASSAS
GYPKPEEGILDSLDVWVIAVIVIAIVVGVAVICVVPYRYLQRRKKKGTYLTDETHREVKF
TSL

>sp|P15529-6|MCP_HUMAN Isoform J of Membrane cofactor protein OS=Homo sapiens OX=9606 GN=CD46
MEPPGRRECPFPSWRFPGLLLAAMVLLLYSFSDACEEPPTFEAMELIG**KPKPYYEIG**ERV

DYKCK**KGYFYIPPL**ATHTI**CDRNHTWLP**VSDDACYRETCPYIRDPLNGQAVPANGTYEFG
YQMHFICNEGYYLIGEEILYCELKGSVAIWSGKPPICEKVLCTPPPKIKNGKHTFSEVEV
FEYLDAVTYSCDPAPGPDPFSLIGESTIYCGDNSVWSRAAPECKVVKCRFPVVENGKQIS
GF**GKKFYYKAT**VMFECDKGFYLDGSDTIVCDSNSTWDPPVPKCLKVSTSSTTKSPASSAS
GYPKPEEGILDSLDVWVIAVIVIAIVVGVAVICVVPYRYLQRRKKKGKADGGAEYATYQT
KSTTPAEQRG

>sp|P15529-15|MCP_HUMAN Isoform K of Membrane cofactor protein OS=Homo sapiens OX=9606 GN=CD46
MEPPGRRECPFPSWRFPGLLLAAMVLLLYSFSDACEEPPTFEAMELIG**KPKPYYEIG**ERV
DYKCK**KGYFYIPPL**ATHTI**CDRNHTWLP**VSDDACYRETCPYIRDPLNGQAVPANGTYEFG
YQMHFICNEGYYLIGEEILYCELKGSVAIWSG**KPPICEKVL**CTPPPKIKNGKHTFSEVEV
FEYLDAVTYSCDPAPGPDPFSLIGESTIYCGDNSVWSRAAPECKVVKCRFPVVENGKQIS
GF**GKKFYYKATV**MFECDKGFYLDGSDTIVCDSNSTWDPPVPK**CLKGYPKPE**EGILDSLDV
WVIAVIVIAIVVGVAVICVVPYRYLQRRKKKGTYLTDETHREVKFTSL

>sp|P15529-7|MCP_HUMAN Isoform L of Membrane cofactor protein OS=Homo sapiens OX=9606 GN=CD46
MEPPGRRECPFPSWRFPGLLLAAMVLLLYSFSDACEEPPTFEAMELIG**KPKPYYEIG**ERV
DYKCK**KGYFYIPPL**ATHTI**CDRNHTWLP**VSDDACYRETCPYIRDPLNGQAVPANGTYEFG
YQMHFICNEGYYLIGEEILYCELKGSVAIWSG**KPPICEKVL**CTPPPKIKNGKHTFSEVEV
FEYLDAVTYSCDPAPGPDPFSLIGESTIYCGDNSVWSRAAPECKVVKCRFPVVENGKQIS
GF**GKKFYYKATV**MFECDKGFYLDGSDTIVCDSNSTWDPPVPK**CLKGYPKPE**EGILDSLDV
WVIAVIVIAIVVGVAVICVVPYRYLQRRKKKGKADGGAEYATYQTKSTTPAEQRG

>sp|P15529-8|MCP_HUMAN Isoform M of Membrane cofactor protein OS=Homo sapiens OX=9606 GN=CD46
MEPPGRRECPFPSWRFPGLLLAAMVLLLYSFSDACEEPPTFEAMELIG**KPKPYYEIG**ERV
DYKCK**KGYFYIPPL**ATHTI**CDRNHTWLP**VSDDACYRETCPYIRDPLNGQAVPANGTYEFG
YQMHFICNEGYYLIGEEILYCELKGSVAIWSGKPPICEKVLCTPPPKIKNGKHTFSEVEV
FEYLDAVTYSCDPAPGPDPFSLIGESTIYCGDNSVWSRAAPECKVVKCRFPVVENGKQIS
GF**GKKFYYKAT**VMFECDKGFYLDGSDTIVCDSNSTWDPPVPKCLKVSTSSTTKSPASSAS
G**PRPTYKPPV**SNYPGYPKPEEGILDSLDVWVIAVIVIAIGKQMVELNMPLTRLNQPLQQS
REAE

>sp|P15529-9|MCP_HUMAN Isoform N of Membrane cofactor protein OS=Homo sapiens OX=9606 GN=CD46
MEPPGRRECPFPSWRFPGLLLAAMVLLLYSFSDACEEPPTFEAMELIG**KPKPYYEIG**ERV
DYKCKKGYFYIPPLATHTI**CDRNHTWLP**VSDDACYRETCPYIRDPLNGQAVPANGTYEFG
YQMHFICNEGYYLIGEEILYCELKGSVAIWSG**KPPICEKVL**CTPPPKIKNGKHTFSEVEV
FEYLDAVTYSCDPAPGPDPFSLIGESTIYCGDNSVWSRAAPECKVVKCRFPVVENGKQIS
GF**GKKFYYKATV**MFECDKGFYLDGSDTIVCDSNSTWDPPVPKCLKG**PRPTYKPPV**SNYPG
YPKPEEGILDSLDVWVIAVIVIAIGKQMVELNMPLTRLNQPLQQSREAE

>sp|P15529-10|MCP_HUMAN Isoform 2 of Membrane cofactor protein OS=Homo sapiens OX=9606 GN=CD46
MEPPGRRECPFPSWRFPGLLLAAMVLLLYSFSDACEEPPTFEAMELIG**KPKPYYEIG**ERV
DYKCK**KGYFYIPPL**ATHTI**CDRNHTWLP**VSDDACYRETCPYIRDPLNGQAVPANGTYEFG
YQMHFICNEGYYLIGEEILYCELKGSVAIWSGKPPICEKVLCTPPPKIKNGKHTFSEVEV
FEYLDAVTYSCDPAPGPDPFSLIGESTIYCGDNSVWSRAAPECKVVKCRFPVVENGKQIS
GF**GKKFYYKAT**VMFECDKGFYLDGSDTIVCDSNSTWDPPVPKCLKVLPPSSTKP**PALSHS**
**TTK**SPASSASGPRPTYKPPVSNYPGYPKPEEGILDSLDVWVIAVIVIAIVVGVAVICVVP
YRYLQRRKKKGTYLTDETHREVKFTSL

>sp|P15529-16|MCP_HUMAN Isoform 3 of Membrane cofactor protein OS=Homo sapiens OX=9606 GN=CD46

MEPPGRRECPFPSWRFPGLLLAAMVLLLYSFSGETCPYIRDPLNGQAVPANGTYEFGYQM
HFICNEGYYLIGEEILYCELKGSVAIWSGKPPICEKVLCTPPPKIKNGKHTFSEVEVFEY
LDAVTYSCDPAPGPDPFSLIGESTIYCGDNSVWSRAAPECKVVKCRFPVVENGKQISGF**G**
**KKFYYKATV**MFECDKGFYLDGSDTIVCDSNSTWDPPVPKCLKVLPPSSTKPPALSHSVST
SSTTKSPASSASGPRPTYKPPVSNYPGYPKPEEGILDSLDVWVIAVIVIAIVVGVAVICV
VPYRYLQRRKKKGKADGGAEYATYQTKSTTPAEQRG

>sp|P08174|DAF_HUMAN Complement decay-accelerating factor OS=Homo sapiens OX=9606 GN=CD55 PE=1 SV=4
MTVARPSVPAALPLLGELPRLLLLVLLCLPAVWGDCGLPPDVPNAQPALEGRTSFPEDTV
ITYKCEESFVKIPGEKDSVICLKGSQWSDIEEFCNRSCEVPTRLNSAS**LKQPYITQN**YFP
VGTVVEYECRPGYRREPSLSPKLTCLQNLKWSTAVEFCKKKSCPNPGEIRNGQIDVPGGI
LFGATISFSCNTGYKLFGSTSSFCLISGSSVQWSDPLPECREIYCPAPPQIDNGIIQ**GER**
**DHYGYRQSV**TYACNKGFTMIGEHSIYCTVNNDEGEWSGPPPECRGKSLTSKVPPTVQKPT
TVNVPTTEVSPTSQKTTTKTTTPNAQATRSTPVSRT**TKHFHETTP**NKGSGTTSGTTRLLS
GHTCFTLTGLLGTLVTMGLLT

>sp|P08174-2|DAF_HUMAN Isoform 1 of Complement decay-accelerating factor OS=Homo sapiens OX=9606 GN=CD55
MTVARPSVPAALPLLGELPRLLLLVLLCLPAVWGDCGLPPDVPNAQPALEGRTSFPEDTV
ITYKCEESFVKIPGEKDSVICLKGSQWSDIEEFCNRSCEVPTRLNSAS**LKQPYITQN**YFP
VGTVVEYECRPGYRREPSLSPKLTCLQNLKWSTAVEFCKKKSCPNPGEIRNGQIDVPGGI
LFGATISFSCNTGYKLFGSTSSFCLISGSSVQWSDPLPECREIYCPAPPQIDNGIIQ**GER**
**DHYGYRQSV**TYACNKGFTMIGEHSIYCTVNNDEGEWSGPPPECRGKSLTSKVPPTVQKPT
TVNVPTTEVSPTSQKTTTKTTTPNAQATRSTPVS**RTTKHFHETTP**NKGSGTTSGTTRLLS
GSRPVTQAGMRWCDRSSLQSRTPGFKRSFHFSLPSSWYYR**AHVFHVDRF**AWDASNHGLAD
LAKEELRRKYTQVYRLFLVS

>sp|P08174-3|DAF_HUMAN Isoform 3 of Complement decay-accelerating factor OS=Homo sapiens OX=9606 GN=CD55
MTVARPSVPAALPLLGELPRLLLLVLLCLPAVWGDCGLPPDVPNAQPALEGRTSFPEDTV
ITYKCEESFVKIPGEKDSVICLKGSQWSDIEEFCNRSCEVPTRLNSAS**LKQPYITQN**YFP
VGTVVEYECRPGYRREPSLSPKLTCLQNLKWSTAVEFCKKKSCPNPGEIRNGQIDVPGGI
LFGATISFSCNTGYKLFGSTSSFCLISGSSVQWSDPLPECREIYCPAPPQIDNGIIQ**GER**
**DHYGYRQSV**TYACNKGFTMIGEHSIYCTVNNDEGEWSGPPPECRGKSLTSKVPPTVQKPT
TVNVPTTEVSPTSQKTTTKTTTPNAQATRSTPVSRT**TKHFHETTP**NKGSGTTSGTTRLLS
ALQVRPFEV**SGSSHISSK**KMMCIL

>sp|P08174-4|DAF_HUMAN Isoform 4 of Complement decay-accelerating factor OS=Homo sapiens OX=9606 GN=CD55
MTVARPSVPAALPLLGELPRLLLLVLLCLPAVWGDCGLPPDVPNAQPALEGRTSFPEDTV
ITYKCEESFVKIPGEKDSVICLKGSQWSDIEEFCNRSCEVPTRLNSAS**LKQPYITQN**YFP
VGTVVEYECRPGYRREPSLSPKLTCLQNLKWSTAVEFCKKKSCPNPGEIRNGQIDVPGGI
LFGATISFSCNTGYKLFGSTSSFCLISGSSVQWSDPLPECREIYCPAPPQIDNGIIQ**GER**
**DHYGYRQSV**TYACNKGFTMIGEHSIYCTVNNDEGEWSGPPPECRGKSLTSKVPPTVQKPT
TVNVPTTEVSPTSQKTTTKTTTPNAQATRSTPVS**RTTKHFHETTP**NKGSGTTSGTTRLLS
VLFM

>sp|P08174-5|DAF_HUMAN Isoform 5 of Complement decay-accelerating factor OS=Homo sapiens OX=9606 GN=CD55
MTVARPSVPAALPLLGELPRLLLLVLLCLPAVWGDCGLPPDVPNAQPALEGRTSFPEDTV
ITYKCEESFVKIPGEKDSVICLKGSQWSDIEEFCNRSCEVPTRLNSAS**LKQPYITQN**YFP
VGTVVEYECRPGYRREPSLSPKLTCLQNLKWSTAVEFCKKKSCPNPGEIRNGQIDVPGGI
LFGATISFSCNTGYKLFGSTSSFCLISGSSVQWSDPLPECREIYCPAPPQIDNGIIQ**GER**
**DHYGYRQSV**TYACNKGFTMIGEHSIYCTVNNDEGEWSGPPPECRGKSLTSKVPPTVQKPT
TVNVPTTEVSPTSQKTTTKTTTPNAQATRSTPVS**RTTKHFHETTP**NKGSGTTSGTTRLLS

**ETVFHRVIQ**DGLDLLA**SRSACLGLPKCWDYRREPPHLARAHVFHVDRF**AWDASNHGLADL
AKEELRRKYTQVYRLFLVS

>sp|P08174-6|DAF_HUMAN Isoform 6 of Complement decay-accelerating factor OS=Homo sapiens OX=9606 GN=CD55
MTVARPSVPAALPLLGELPRLLLLVLLCLPAVWGDCGLPPDVPNAQPALEGRTSFPEDTV
ITYKCEESFVKIPGEKDSVICLKGSQWSDIEEFCNRSCEVPTRLNSAS**LKQPYITQN**YFP
VGTVVEYECRPGYRREPSLSPKLTCLQNLKWSTAVEFCKKKSCPNPGEIRNGQIDVPGGI
LFGATISFSCNTGYKLFGSTSSFCLISGSSVQWSDPLPECREIYCPAPPQIDNGIIQ**GER**
**DHYGYR**QSVTYACNKGFTMIGEHSIYCTVNNDEGEWSGPPPECRGKSLTSKVPPTVQKPT
TVNVPTTEVSPTSQKTTTKTTTPNAQGTETPS**VLQKHTTEN**VSATRTPPTPQKPTTVNVP
ATIVTP**TPQKPTTINV**PATGVSSTPQRHTIVNVSATGTLPTLQKPTRANDSATKSPAAAQ
TSFISKTLSTKTPSAAQNPMMTNASATQATLTAQKFTTAKVAFTQSPSAAPTRSTPVS**RT**
**TKHFHETTP**NKGSGTTSGTTRLLSGHTCFTLTGLLGTLVTMGLLT

>sp|P08174-7|DAF_HUMAN Isoform 7 of Complement decay-accelerating factor OS=Homo sapiens OX=9606 GN=CD55
MTVARPSVPAALPLLGELPRLLLLVLLCLPAVWGDCGLPPDVPNAQPALEGRTSFPEDTV
ITYKCEESFVKIPGEKDSVICLKGSQWSDIEEFCNRSCEVPTRLNSAS**LKQPYITQN**YFP
VGTVVEYECRPGYRREPSLSPKLTCLQNLKWSTAVEFCKKKSCPNPGEIRNGQIDVPGGI
LFGATISFSCNTGYKLFGSTSSFCLISGSSVQWSDPLPECREIYCPAPPQIDNGIIQ**GER**
**DHYGYR**QSVTYACNKGFTMIGEHSIYCTVNNDEGEWSGPPPECRGKSLTSKVPPTVQKPT
TVNVPTTEVSPTSQKTTTKTTTPNAQGTETPS**VLQKHTTEN**VSATRTPPTPQKPTTVNVP
ATIVTPTPQKPTTINVPATGVSS**TPQRHTIVNV**SATGTLPTLQKPTRANDSATKSPAAAQ
TSFISKTLSTKTPSAAQNPMMTNASATQATLTAQKFTTAKVAFTQSPSAARKSTNVHSPV
TNGLKSTQRFPSAHITATRSTPVS**RTTKHFHETTP**NKGSGTTSGTTRLLSGHTCFTLTGL
LGTLVTMGLLT

>sp|P17927-E|CR1_HUMAN Complement receptor type 1 OS=Homo sapiens OX=9606 GN=CR1 PE=1 SV=3
MGASSPRSPEPVGPPAPGLPFCCGGSLLAVVVLLALPVAWGQCNAPEWLPFARPTNLTDE
FEFPIGTYLNYECRPGYSGRPFSIICLKNSVWTGAKDRCRRKSCRNPPDPVNGMVHVIKG
IQFGSQIKYSCTKGYRLIGSSSATCIISGDTVIWDNETPICDRIPCGLPPTITNGDFIST
N**RENFHYGSV**VTYRCNPGSGGRKVFELVGEPSIYCTSNDDQVGIWSGPAPQCIIPNKCTP
PNVENGILVSDNRSLFSLNEVVEFRCQPGFVMKGPRRVKCQALNKWEPELPS**CSRVCQPP**
**P**DVLHAERTQRDKDNFSPGQEVFYSCEPGYDLRGAASMRCTPQGDWSPAAPTCEVKSCDD
FMGQLLNGRVLFPVNLQLGAKVDFVCDEGFQLKGSSASYCVLAGMESLWNSSVPVCEQIF
CPSPPVIPNGRHTGKPLEVFPFGKTVNYTCDPHPDRGTSFDLIGESTIRCTSDPQGNGVW
SSPAPRCGILGHCQAPDHFLFAKLKTQTNASDFPIGTSLKYEC**RPEYYGRPF**SITCLDNL
VWSSPKDVCKRKSCKTPPDPVNGMVHVITDIQVGSRINYSCTTGH**RLIGHSSAE**CILSGN
AAHWSTKPPIC**QRIPCGLPP**TIANGDFISTN**RENFHYGSV**VTYRCNPGSGGRKVFELVGE
PSIYCTSNDDQVGIWSGPAPQCIIPNKCTPPNVENGILVSDNRSLFSLNEVVEFRCQPGF
VMKGPRRVKCQALNKWEPELPS**CSRVCQPPP**DVLHAERTQRDKDNFSPGQEVFYSCEPGY
DLRGAASMRCTPQGDWSPAAPTCEVKSCDDFMGQLLNGRVLFPVNLQLGAKVDFVCDEGF
QLKGSSASYCVLAGMESLWNSSVPVCEQIFCPSPPVIPNGRHTGKPLEVFPFGKAVNYTC
DPHPDRGTSFDLIGESTIRCTSDPQGNGVWSSPAPRCGILGHCQAPDHFLFAKLKTQTNA
SDFPIGTSLKYEC**RPEYYGRPF**SITCLDNLVWSSPKDVCKRKSCKTPPDPVNGMVHVITD
IQVGSRINYSCTTGH**RLIGHSSAE**CILS**GNTAHWSTKPPICQRIP**CGLPPTIANGDFIST
N**RENFHYGSV**VTYRCNLGSRGRKVFELVGEPSIYCTSNDDQVGIWSG**PAPQCIIPN**KCTP
PNVENGILVSDNRSLFSLNEVVEFRCQPGFVMKGPRRVKCQALNKWEPELPS**CSRVCQPP**
**P**EILHGE**HTPSHQDNF**SPGQEVFYSCEPGYDLRGAASLHCTPQGDWSP**EAPRCAVKS**CDD
FL**GQLPHGRVL**FPLNLQLGAKVSFVCDEGFRLKGSSVSHCVLVGMRSLWNN**SVPVCEHIF**
CPNPPAILNGRHTGTPSGDIPYGKEISYTCDPHPDRGMTFNLIGESTIRCTSDPHGNGVW
SSPAPRCELS**VRAGHCKTP**EQFPFASPTIPINDFEFPVGTSLNYECRPGYFGKMFSISCL
ENLVWSSVEDNCRRKSCGPPPEPFNGMVHINTDTQFGSTVNYSCNEGFRLIGSPSTTCLV
SGNNVTWDKKAPICEIISCEPPPTISNGDFYSNN**RTSFHNGTV**VTYQCHTGPDGEQLFEL

VGERSIYCTSKDDQVGVWSS**PPPRCISTN**KCTAPEVENAIRVPGNRSFFSLTEIIRFRCQ
PGFVMVGSHTVQCQTNGRWG**PKLPHCSRVCQPPP**EILHGE**HTLSHQDNF**SPGQEVFYSCE
PSYDLRGAASLHCTPQGDWSP**EAPRCTVKS**CDDFL**GQLPHGRVL**LPLNLQLGAKVSFVCD
EGFRLKGRSASHCVLAGMKALWNSSVPVCEQIFCPNPPAILNGRHTGTPFGDIPYGKEIS
YACDTHPDRGMTFNLIGESSIRCTSDPQGNGVWSSPAPRCELSVP**AACPHPPKI**QNGHYI
GGHVSLYLPGMTISYICDPGYLLVGKGFIFCTDQGIWSQLDHYCKEVNCSFPLFMNGISK
ELEM**KKVYHYGDYVTLK**CEDGYTLEGSPWSQCQADDRWDPPLAKC**TSRTHD**

>sp|P17927-T|CR1_HUMAN Complement receptor type 1 OS=Homo sapiens OX=9606 GN=CR1 PE=1 SV=3
ALIVGTLSGTIFFILLIIFLSWIIL

>sp|P17927-C|CR1_HUMAN Complement receptor type 1 OS=Homo sapiens OX=9606 GN=CR1 PE=1 SV=3
**KHRKGNNAHENPKEVAIHLHSQGGSSVHPRTL**QTNEENSRVLP

>sp|Q03591|FHR1_HUMAN Complement factor H-related protein 1 OS=Homo sapiens OX=9606 GN=CFHR1 PE=1 SV=2
MWLLVSVILISRISSVGGEATFCDF**PKINHGILY**DEEKYKPFSQVPTGEVFYYSCEYNFV
SPSKSFWTRITCTEEGWSPTPK**CLRLCFFPF**VENGHSESSGQTHLEGDTVQIICNTGYRL
QNNENNISCVERGWSTPPKCRSTDTSCVNPPT**VQNAHILSR**QMSKYPSGERVRYECRSPY
EMFGDEEVMCLNGNWTEPPQCKD**STGKCGPPP**PIDNGDITSFPLSVYAPASSVEYQCQNL
YQLEGNKRITCRNGQWSEP**PKCLHPCVISR**EIMENYNIALRWTA**KQKLYLRTG**ESAEFVC
KRGYRL**SSRSHTLRTTCWDGK**LEYPTCAKR

>sp|P36980|FHR2_HUMAN Complement factor H-related protein 2 OS=Homo sapiens OX=9606 GN=CFHR2 PE=1 SV=1
MWLLVSVILISRISSVGGEAMFCDF**PKINHGILY**DEEKYKPFSQVPTGEVFYYSCEYNFV
SPSKSFWTRITCAEEGWSPTPK**CLRLCFFPF**VENGHSESSGQTHLEGDTVQIICNTGYRL
QNNENNISCVERGWSTPPKCRSTISAEKCGPPPPIDNGDITSFLLSVYAPGSSVEYQCQN
LYQLEGNNQITCRNGQWSEPPKCLDPCVISQE**IMEKYNIKL**KWTN**QQKLYSRTG**DIVEFV
C**KSGYHPTKSHSFRA**MCQNGKLVYPSCEEK

>sp|P36980-2|FHR2_HUMAN Isoform Short of Complement factor H-related protein 2 OS=Homo sapiens OX=9606 GN=CFHR2
MWLLVSVILISRISSVGGEAMFCDF**PKINHGILY**DEEKYKPFSQVPTGEVFYYSCEYNFV
SPSKSFWTRITCAEEGWSPTPK**CLRLCFFPF**VENGHSESSGQTHLEGDTVQII**CNTGYRL**
**QN**NENNISCVERGWSTPPKCRSTSSSVEYQCQNLYQLEGNNQITCRNGQWSEPPKCLDPC
VISQE**IMEKYNIKL**KWTN**QQKLYSRTG**DIVEFVC**KSGYHPTKSHSFRA**MCQNGKLVYPSC
EEK

>sp|Q02985|FHR3_HUMAN Complement factor H-related protein 3 OS=Homo sapiens OX=9606 GN=CFHR3 PE=1 SV=2
MLLLINVILTLWVSCANGQVKPCDF**PDIKHGGLF**HEN**MRRPYFPVAVGKYYSYY**CDEHFE
TPSGSYWDYIHCTQNGWSPAVPC**LRKCYFPYL**ENG**YNQNYGRKF**VQGNSTEVACHPGYGL
PKAQTTVTCTEKGWS**PTPRCIRVR**TCSKSDIEIENGFISESSSIYILNKEIQYKCKPGYA
TADGNSSGSITCLQNGWSAQPICINSSEKCGPPPPISNGDTTSFLLKVYVPQSRVEYQCQ
PYYELQGSNYVTCSNGEWS**EPPRCIHPCIITE**ENMNKNNIKLKGR**SDRKYYAKTG**DTIEF
MCKLGYNANTSILS**FQAVCREGI**VEYPRCE

>sp|Q02985-2|FHR3_HUMAN Isoform 2 of Complement factor H-related protein 3 OS=Homo sapiens OX=9606 GN=CFHR3
MLLLINVILTLWVSCANGQVKPCDF**PDIKHGGLF**HEN**MRRPYFPVAVGKYYSYY**CDEHFE
TPSGSYWDYIHCTQNGWSPAVPC**LRKCYFPYL**ENG**YNQNYGRKF**VQGNSTEVACHPGYGL
PKAQTTVTCTEKGWSPTPRCIRVNSSEKCGPPPPISNGDTTSFLLKVYVPQSRVEYQCQP
YYELQGSNYVTCSNGEWS**EPPRCIHPCII**TEENMNKNNIKLKGR**SDRKYYAKTG**DTIEFM

CKLGYNANTSILS**FQAVCREGI**VEYPRCE

>sp|Q92496|FHR4_HUMAN Complement factor H-related protein 4 OS=Homo sapiens OX=9606 GN=CFHR4 PE=1 SV=3
MLLLINVILT**LWVSCANGQ**EVKPCDFPEIQHGGLYYKS**LRRLYFPAA**AGQSYSYYCDQNF
VTPSGSYWDYIHCTQDGWSPTVP**CLRTCSKSD**VEIENGFISESSSIYILNEETQYNCKPG
YATAEGNSSGSITCLQNGWSTQPICIKFCDMPVFENSRAKSNGMWFKLHDTLDYECYDGY
ESSYGNTTDSIVCGEDGWS**HLPTCYNSS**ENCGPPPPISNGDTTSFPQKVYLPWSRVEYQC
QSYYEL**QGSKYVTCS**NGDWSEPPRCISMKPCEF**PEIQHGHLYYE**N**TRRPYFPVA**TGQSYS
YYCDQNFVTPSGSYWDYIHCTQDGWLPTVP**CLRTCSKSD**IEIENGFISESSSIYILNKEI
QYKCKPGYATADGNSSGSITCLQNGWSAQPICIKFCDMPVFENSRAKSNGM**RFKLHDTLD**
YECYDGYEISYGNTTGSIVCGEDGWSHFPTCYNSSEKCGPPPPISNGDTTSFLLKVYVPQ
SRVEYQCQSYYELQGSNYVTCSNGEWS**EPPRCIHPCII**TEENMNKNNIQLKGK**SDIKYYA**
**KTG**DTIEFMCKLGYNANTSVLSFQAVCREGIVEYPRCE

>sp|Q92496-2|FHR4_HUMAN Isoform 2 of Complement factor H-related protein 4 OS=Homo sapiens OX=9606 GN=CFHR4
MLLLINVILTLWVSCANGQVKPCDFPEIQ**HGGLYYKSLRRLYFPAA**AGQSYSYYCDQNFV
TPSGSYWDYIHCTQDGWSPTVP**CLRTCSKSD**VEIENGFISESSSIYILNEETQYNCKPGY
ATAEGNSSGSITCLQNGWSTQPICIKFCDMPVFENSRAKSNGMWFKLHDTLDYECYDGYE
SSYGNTTDSIVCGEDGWSHLPTCYNSSENCGPPPPISNGDTTSFPQKVYLPWSRVEYQCQ
SYYEL**QGSKYVTCS**NGDWSEPPRCISMKPCEF**PEIQHGHLYYE**N**TRRPYFPVA**TGQSYSY
YCDQNFVTPSGSYWDYIHCTQDGWL**PTVPCLRTCSKSD**IEIENGFISESSSIYILNKEIQ
YKCKPGYATADGNSSGSITCLQNGWSAQPICIKFCDMPVFENSRAKSNGM**RFKLHDTLD**Y
ECYDGYEISYGNTTGSIVCGEDGWSHFPTCYN**SSEKCGPPP**PISNGDTTSFLLKVYVPQS
RVEYQCQSYYELQGSNYVTCSNGEWS**EPPRCIHPCII**TEENMNKNNIQLKGK**SDIKYYAK**
**TG**DTIEFMCKLGYNANTSVLSFQAVCREGIVEYPRCE

>sp|Q92496-3|FHR4_HUMAN Isoform 3 of Complement factor H-related protein 4 OS=Homo sapiens OX=9606 GN=CFHR4
MLLLINVILT**LWVSCANGQ**EVKPCDFPEIQ**HGGLYYKSLRRLYFPAA**AGQSYSYYCDQNF
VTPSGSYWDYIHCTQDGWSPTVP**CLRTCSKSD**IEIENGFISESSSIYILNKEIQYKCKPG
YATADGNSSGSITCLQNGWSAQPICIKFCDMPVFENSRAKSNGM**RFKLHDTLD**YECYDGY
EISYGNTTGSIVCGEDGWSHFPTCYNSSEKCGPPPPISNGDTTSFLLKVYVPQSRVEYQC
QSYYELQGSNYVTCSNGEWSEP**PRCIHPCII**TEENMNKNNIQLKGK**SDIKYYAKTG**DTIE
FMCKLGYNANTSVLS**FQAVCREGI**VEYPRCE

>sp|Q9BXR6|FHR5_HUMAN Complement factor H-related protein 5 OS=Homo sapiens OX=9606 GN=CFHR5 PE=1 SV=1
MLLLFSVILISWVSTVGGEGTLCD**FPKIHHGFLY**DEEDYNPFSQVPTGEVFYYSCEYNFV
SPSKSFWTRITCTEEGWSPTPKCLRMCSFPF**VKNGHSESS**GLIHLEGDTVQIICNTGYSL
QNNEKNISCVERGWS**TPPICSFTKGECHVPIL**EANVDAQPKKESYKVGDVLKFSCRKNLI
RVGSDSVQCYQFGWSPNFPTCKGQVRSCGPPPQLSNGEVKEIRKEEYGHNEVVEYDCNPN
FIINGPKKIQCVDGEWTTLPTCVEQVKTCGYIPELEYGYVQPSVPPYQHGVSVEVNCRNE
YAMIGNNMITCINGIWTELPM**CVATHQLKR**CKIAGVNIKTLLKLSG**KEFNHNSRI**RYRCS
DIFRYRHSVCINGKWNPEVDCTEKREQFCPPPPQIPNAQNMTTTVNYQDGEKVAVLCKEN
YLLPEAKEIVCKDGRWQ**SLPRCVEST**AYCGPPPSINNGDTTSFPLSVYPPGSTVTYRCQS
FYKLQGSVTVTCRNKQWSEPPRCLDPCVVSEENMNKNNIQLKWRNDGKLYAKTGDAVEFQ
CKFPHKAMISSPPFRAICQEGKFEYPICE

>sp|P04003|C4BPA_HUMAN C4b-binding protein alpha chain OS=Homo sapiens OX=9606 GN=C4BPA PE=1 SV=2
**MHPPKT**PSGALHRKRKMAAWPFSRLWKVSDPILFQMTLIAALLPAVLGNCGPPPTLSFAA
PMDITLTETRFKTGTTLKYTCLPG**YVRSHSTQT**LTCNSDGEWVYN**TFCIYKRCRHPGEL**R
NGQVEIKTDLSFGSQIEFSCSEGFFLIGSTTSRCEVQDRGVGWSHPLPQCEIVKCKPPPD
IRNGRHSGEENFYAYGFSVTYSCDPRFSLLGHASISCTVENETIGVWRPSPPTCEKITCR

KPDVSHGEMVSGFGPIYNYKDTIVFKCQKGFVLRGSSVIHCDADSKWNPSPPACEPNSCI
NLPDIPHASWETYPRPTKEDVYVVGTVL**RYRCHPGYK**PTTDEPTTVICQKNLRWTPYQGC
EALCCPEPKLNNG**EITQHRKSRPANHCVYFY**GDEI**SFSCHETSR**FSAICQGDGTWSPRTP
SCGDICNFP**PKIAHGHYKQSSSYSFFK**EEIIYECDKGYILVGQAK**LSCSYSHWS**APAPQC
KALCRKPELVNGRLSVDKDQYVEPENVTIQCDSGYGVVGPQSITCSGNRTWYPEVPKCEW
ETPEGCEQVLTGKRLMQCLPNPEDVKMALEVYKLSLEIEQLELQRDSARQSTLDKEL

>sp|P20851|C4BPB_HUMAN C4b-binding protein beta chain OS=Homo sapiens OX=9606 GN=C4BPB PE=1 SV=1
MFFWCACCLMVAWRVSASDAEHCPELPPVDNSIFVAKEVEGQILGTYVC**IKGYHLVGK**KT
LFCNASKEWDNTTTE**CRLGHCPDP**VLVNGEFSSSGPVNVSDKITF**MCNDHYILK**GSNRSQ
CLEDHTWAPPFPICKSRDCDPPGNPVHGYFEGNNFTLGSTISYYCEDRYYLVGVQEQQCV
DGEWSSALPVCKLIQEAPKPECEKALLAFQESKNLCEAMENFMQQLKESGMTMEELKYSL
ELKKAELKAKLL

>sp|P20851-2|C4BPB_HUMAN Isoform 2 of C4b-binding protein beta chain OS=Homo sapiens OX=9606 GN=C4BPB
MFFWCACCLMVAWRVSASDEHCPELPPVDNSIFVAKEVEGQILGTYVC**IKGYHLVGK**KTL
FCNASKEWDNTTTE**CRLGHCPDP**VLVNGEFSSSGPVNVSDKITF**MCNDHYILK**GSNRSQC
LEDHTWAPPFPICKSRDCDPPGNPVHGYFEGNNFTLGSTISYYCEDRYYLVGVQEQQCVD
GEWSSALPVCKLIQEAPKPECEKALLAFQESKNLCEAMENFMQQLKESGMTMEELKYSLE
LKKAELKAKLL

**16 unique entries/genes**
**46 sequences**

**Species with detected HBM variations**

>sp|P01024|CO3_HUMAN Complement C3 OS=Homo sapiens OX=9606 GN=C3 PE=1 SV=2
MGPTSGPSLLLLLLTHLPLALGSPMYSIITPNILRLESEETMVLEAHDAQGDVPVTVTVH
DFPGKKLVLSSEKTVLTPATNHMGNVTFTIPANREFKSEKGRNKFVTVQATFGTQVVEKV
VLVSLQSGYLFIQTDKTIYTPGSTVLYRI**FTVNHKLLP**VGRTVMVNIENPEGIPVKQDSL
SSQNQLGVLPLSWDIPELVNMGQW**KIRAYYENS**PQQVFSTEFEVKEYVLPSFEVIVEPTE
KFYYIYNEKGLEVTITARFLYGKKVEGTAFVIFGIQDGEQRISLPESLKRIPIEDGSGEV
VLSRKVLLDGVQNPRAEDLVGKSLYVSATVILHSGSDMVQAERSGIPIVTS**PYQIHFTKT**
PKYFKPGMPFDLMVFVTNPD**GSPAYRVPV**AVQGEDTVQSLTQGDGVAKL**SINTHPSQK**PL
SITVRTKKQELSEAEQATRTMQALPYSTVGNSNNYLHLSVLRTELRPGETLNVNFLLR**MD**
**RAHEAKI**RYYTYLIMNKGRLLKAGRQVREPGQDLVVLPLSITTDFIPSFRLVAYYTLIGA
SGQREVVADSVWVDVKDSCVGSLVVKSGQSEDRQPVPGQQMTLKIEGDHGARVVLVAVDK
GVFVLNKKNKLTQSKIWDVVEKADIGCTPGSGKDYAGVFSDAGLTFTSSSGQQTAQRAEL
QCPQPAARRRRSVQLTEKRMDKVGKYPKELRKCCEDGMRENPMRFSCQRRTRFISLGEAC
KKVFLDCCNYITE**LRRQHARASHLGLA**RSNLDEDIIAEENIVSRSEFPESWLWNVEDLKE
PPKNGISTKLMNIFLKDSITTWEILAVSMSDKKGICVADPFEVTVMQDFFIDLRLPYSVV
RNEQVEIRAVLYNYRQNQELKVRVELLH**NPAFCSLATTKRRHQQTV**TIPPKSSLSVPYVI
VPLKTGLQEVEV**KAAVYHHFISD**GVRKSLKVVPEGIRMNKTVAVRTLDPERLGREGVQKE
DIPPADLSDQVPDTESETRILLQGTPVAQMTEDAVDA**ERLKHLIVT**PSGCGEQNMIGMTP
TVIAVHYL**DE**T**E**QW**E**KFGLEKRQGALELIKKGYTQQLAFRQPSSAFAAFVKRAPSTWLTA
YVVKVFSLAVNLIAID**SQVLCGAVK**WLILEKQKPDGVFQEDAPVIHQEMIGGLRNNNEKD
MALTAFVLISLQEAKDICEEQVNSLPGSITKAGDFLEANYMNLQRSYTVAIAGYALAQMG
RLKGPLLNKFLTTAKDKNRWEDPGKQLYNVEATSYALLALLQLKDFDFVPPVVRWLNEQR
YYGGGYGSTQATFMVFQALAQYQKDAPDHQELNLDVSLQLPSRSSKI**THRIHWESA**SLLR
SEETKENEGFTVTAEGKGQGTLSVVTMYHAKAKDQLTCNKFDLKVTIKPAPETEKRPQDA
KNTMILEICTRYRGDQDATMSILDISMMTGFAPDTDDLKQLANGVDRYISKYELDKAFSD
RNTLIIYLDKVSHSEDDCL**AFKVHQYFNVE**LIQPGAVKVYAYYNLEESC**TRFYHPEKE**DG
KLNKLCRDELCRCAEENCFIQKSDDKVTLEERLDKACEPGVDYVYKTRLVKVQLSNDFDE
YIMAIEQTIKSGSDEVQVGQQRTFISPIKCREALKL**EEKKHYLMW**GLSSDFWGEKPNLSY
IIGKDTWVEHWPEEDECQDEENQKQCQDLGAFTESMVVFGCPN

**VAR 070941, CA213419, RCV000077796, RCV000202831, RCV000765476, RCV000963525, RCV001130000, RCV001130001, RCV001130002, rs147859257, 155, K>Q, ARMD9; results in resistance to proteolytic inactivation by CFH and CFI (UniProt), MACULAR DEGENERATION, AGE-RELATED, 9, SUSCEPTIBILITY TO (ClinVar), Age related macular degeneration 9 (ClinVar), Atypical hemolytic-uremic syndrome with C3 anomaly (ClinVar), Complement component 3 deficiency (ClinVar)**
**VAR 063215, CA9129171, COSM3797670, rs117793540, 735, R>W, AHUS5 (UniProt), urinary tract (Cosmic)**
**CA9129168, RCV001129557, RCV001129558, RCV001129559,rs578116271, 736, R>Q, Age related macular degeneration 9 (ClinVar), Atypical hemolytic-uremic syndrome with C3 anomaly (ClinVar), Complement component 3 deficiency (ClinVar)**
**RCV001136442, RCV001136443, RCV001136444, rs1967565177, 873, C>R, Age related macular degeneration 9 (ClinVar), Atypical hemolytic-uremic syndrome with C3 anomaly (ClinVar), Complement component 3 deficiency (ClinVar)**
**CA403633250, RCV001328277, RCV001880191, rs1443451793, 881, R>H, Variant assessed as Somatic; 0.0 impact. (NCI-TCGA), Atypical hemolytic-uremic syndrome (ClinVar)**

**CA9128779, RCV000311409, RCV000350872, RCV000398615, RCV001859955, rs750654763, 1100, L>P, Age related macular degeneration 9 (ClinVar), Atypical hemolytic-uremic syndrome with C3 anomaly (ClinVar), Complement component 3 deficiency (ClinVar)**
**AR 063220, 1464, H>D, Hemolytic uremic syndrome atypical 5 (AHUS5) (UniProt)**

>sp|P00751|CFAB_HUMAN Complement factor B OS=Homo sapiens OX=9606 GN=CFB PE=1 SV=2
MGSNLSPQLCLMPFILGLLSGGVTTTPWSLARPQGSCSLEGVEIKGGSFRLLQEGQALEY
VCPSGFYPYPVQTRTCRSTGSWSTLKTQDQKTVRKAE**CRAIHCPRP**HDFENGEYW**PRSPY**
**YNVS**DEISFHCYDGYTLRGSANRTCQVNGRWSGQTAICDNGAGYCSNPGIPIGTRKVGSQ
YRLEDSVTYHCSRGLTLRGSQRRTCQEGGSWSGTEPSCQDSFMYDTPQEVAEAFLSSLTE
TIEGVDAEDGHGPGEQQKRKIVLDPSGSMNIYLVLDGSDSIGASNFTGAKKCLVNLIE**KV**
**ASYGVKP**RYGLV**TYATYPKIW**VKVSEADSSNADWVTKQLNEI**NYEDHKLKS**GTNTKKALQ
AVYSMMSWPDDVPPEGWNRTRHVIILMTDGLHNMGGDPITVIDEIRDLLYIGKDRKNPRE
DYLDVYVFGVGPLVNQVNINALASKKDNEQHVFKVKDMENLEDVFYQMIDESQSLSLCG**M**
**VWEHRKGTDYHKQPW**QAKISVIR**PSKGHESCM**GAVVSEYFVLTAAHCFTVDDKEHSIKVS
VGGEKRDLEIEVVLFHPNYNINGKKEAGIPEFYDYDVALIKLKNKLKYGQTIRPICLPCT
EGTTRALRLPPTTTCQQQKEELLPAQDIKALFVSEEEKKLT**RKEVYIKNG**DKKGSCERDA
Q**YAPGYDKVK**DISEVVTPRFLCTGGVSPYADPNTCRGDSGG**PLIVHKRSR**FIQVGVISWG
VVDVCKNQKRQK**QVPAHARDF**HINLFQVLPWLKEKLQDEDLGFL

**CA136894909, RCV001155151, RCV001155152, rs374738591, 306, K>R, Macular degeneration (ClinVar), Atypical hemolytic-uremic syndrome with B factor anomaly (ClinVar)**
**CA3728362, RCV000282119, RCV000405241, rs138207668, 508, H>Q, Macular degeneration (ClinVar), Atypical hemolytic-uremic syndrome with B factor anomaly (ClinVar)**

>sp|P00751-2|CFAB_HUMAN Isoform 2 of Complement factor B OS=Homo sapiens OX=9606 GN=CFB
MGSNLSPQLCLMPFILGLLSGGVTTTPWSLARPQGSCSLEGVEIKGGSFRLLQEGQALEY
VCPSGFYPYPVQTRTCRSTGSWSTLKTQDQKTVRKAE**CRAIHCPRP**HDFENGEYW**PRSPY**
**YNVS**DEISFHCYDGYTLRGSANRTCQVNGRWSGQTAICDNGAGYCSNPGIPIGTRKVGSQ
YRLED**SVTYHCSRG**LTLRGSQRRTCQEGGSWSGTEPSCQDSFMYDTPQEVAEAFLSSLTE
TIEGVDAEDGHGPGEQQKRKIVLDPSGSMNIYLVLDGSDSIGASNFTGAKKCLVNLIE**KV**
**ASYGVKP**RYGLV**TYATYPKIW**VKVSEADSSNADWVTKQLNEI**NYEDHKLKS**GTNTKKALQ
AVYSMMSWPDDVPPEGWNRTRHVIILMTDGLHNMGGDPITVIDEIRDLLYIGKDRKNPRE
DYLDVYVFGVGPLVNQVNINALASKKDNEQHVFKVKDMENLEDVFYQMIDESQSLSLCG**M**
**VWEHRKGTDYHKQPW**QAKISVIR**PSKGHESCM**GAVVSEYFVLTAAHCFTVDDKEHSIKVS
VGKDATEGPGLHLCSPGNTSHFLQI**LHSTHPQCS**PIPCTPDQSGMGEDVKLGMTRGQRQE
AAHKEVVPTLLLQEGRSGTWR

**CA136894909, RCV001155151, RCV001155152, rs374738591, 306, K>R, Macular degeneration (ClinVar), Atypical hemolytic-uremic syndrome with B factor anomaly (ClinVar) [By similarity]**
**CA3728362, RCV000282119, RCV000405241, rs138207668, 508, H>Q, Macular degeneration (ClinVar), Atypical hemolytic-uremic syndrome with B factor anomaly (ClinVar) [By similarity]**

>sp|P05156|CFAI_HUMAN Complement factor I OS=Homo sapiens OX=9606 GN=CFI PE=1 SV=2
MKLLHVFLLFLCFHLRFCKVTYTSQEDLVEKKCLAKKYTHLSCDKVFCQPWQRCIEGTCV
CKLPYQCPKNGTAVCATNRRSFPTYCQQKS**LECLHPGTK**FLNNGTCTAEGKFS**VSLKHGN**

**TD**SEGIVEVKLVDQDKTMFICKSSWSMREANVACLDLGFQQGADTQRRFKLSDLSINS**TE**
**CLHVHCRGL**ETSLAECTFTKRRTMGYQDFADVVCYTQKADSPMDDFFQCVNGKYISQMKA
CDGINDCGDQSDELCCKACQ**GKGFHCKSG**VCIPSQYQCNGEVDCITGEDEVGCAGFASVT
QEETEILTADMDAERRRIKSLLPKLSCGV**KNRMHIRRK**RIVGGKRAQLGDLPWQVAIKDA
SGITCGGI**YIGGCWILT**AAHCLR**ASKTHRYQI**WTTVVDWIHPDLKRIVIEYVDRIIFHEN
YNAGTYQNDIALIEMKKDG**NKKDCELPR**SIPACVPWSPYLFQPNDTCIVSGWGREKDNER
VFSLQWGEVKLISNCSKFYGNRFYEKEMECAGTYDGSIDACKGDSGGPLVCMDANNVTYV
WGVVSWGENCGKPEFPGVYTKVANYF**DWISYHVGRP**FISQYNV

**CA357865066, RCV001328252, rs1478686846, 98, T>A, Atypical hemolytic-uremic syndrome (ClinVar)**
**VAR 063666, CA278398, rs141853578, 119, G>R, AHUS3 and ARMD13; the mutant is both expressed and secreted at lower levels than wild-type protein; mediates C3 degradation to a lesser extent than that of controls (UniProt)**
**VAR 063667, CA3042233, RCV000973356, rs75612300, 183, H>R, Hemolytic uremic syndrome atypical 3 (AHUS3) (UniProt)**
**CA3042231, RCV000778715, RCV001856163, rs368615806, 187, R>*, Atypical hemolytic-uremic syndrome with I factor anomaly (ClinVar)**
**CA3042230, COSM1309619, RCV001148815, RCV001858976, rs143366614, 187, R>Q, Atypical hemolytic-uremic syndrome with I factor anomaly (ClinVar), urinary tract (Cosmic)**
**CA3042165, RCV001148813, rs547901965, 261, G>S, Atypicalhemolytic-uremic syndrome with I factor anomaly (ClinVar)**
**RCV001328246, rs759676430, 336, R>*, Atypical hemolytic-uremic syndrome (ClinVar)**
**CA3042011, RCV000386149, rs763931500, 371, G>V, Atypicalhemolytic-uremic syndrome with I factor anomaly (ClinVar)**
**CA357858724, RCV001029951, rs1579173999, 373, C>S, Atypical hemolytic-uremic syndrome with I factor anomaly (ClinVar)**
**CA357857740, RCV001029952, rs1373768125, 387, T>I, Atypical hemolytic-uremic syndrome with I factor anomaly (ClinVar)**
**CA357857713, RCV000786883, rs1292929833, 389, R>C, Atypical hemolytic-uremic syndrome with I factor anomaly (ClinVar)**
**CA3041835, RCV000505641, RCV001865643, rs200973120, 570, S>T, Atypical hemolytic-uremic syndrome with I factor anomaly (ClinVar)**

>sp|P00746|CFAD_HUMAN Complement factor D OS=Homo sapiens OX=9606 GN=CFD PE=1 SV=5
**MHSWER**LAVLVLLGAAACAAPPRGRILGGREAEAHARPYMASVQLNGAHLCGGVLVAEQW
VLSAAHCLEDAADGKVQVLLGAHSLSQPEPSKRLYDVLRAVPHPDSQPDTIDHDLLLLQL
SEKATLGPAVRPLPWQRVDRDVAPGTLCDVAGW**GIVNHAGRR**PDSLQHVLLPVLDRATC**N**
**RRTHHDGAI**TERLMCAESNRRDSCKGDSGGPLVCGGVLEGVVTSGSRVCGNRKKPGIYTR
VASYAAWIDSVLA

**CA9026370, RCV000767925, rs373019471, 155, I>V, Recurrent Neisseria infections due to factor D deficiency (ClinVar)**

>sp|P27918|PROP_HUMAN Properdin OS=Homo sapiens OX=9606 GN=CFP PE=1 SV=2
MITEGAQAPRLLLPPLLLLLTLPATGSDPVLCFTQYEESSGKCKGLLGGGVSVEDCCLN**T**
**AFAYQKRSGGLCQPCRSPR**WSLWSTWAPCSVTCSEG**SQLRYRRCV**G**WNGQCSGKV**APGTL

EWQLQACEDQQCCPEMGGWSGWGPWEPCSVTCSKGTRTRR**RACNHPAPKCGGHCPGQ**AQE
SEACD**TQQVCPTHG**AWATWG**PWTPCSASCHGGPHEPKE**TRSRKCSAPEPSQKPPGKPC**PG**
**LAYEQRRCTGLP**PCPVAGGWGPWGPVSPCPVTCGLGQTMEQ**RTCNHPVPQ**HGGPFCAGD**A**
**TRTHICNTAV**PCPVDGEWDSWGEWSPCIRRNMKSISCQEIPGQQSRGRTCRGR**KFDGHRC**
**AG**QQ**QDIRHCYSIQHCPLK**GSWSEWSTWGLCMPPCGPNPTRARQRLCTP**LLPKYPPTV**SM
VEGQGEKNVTFWGRPLPRCEELQGQKLVVEEK**RPCLHVPAC**KDPEEEEL

**VAR 002002, CA121395, COSM296671, RCV000011932, rs132630259, 100, R>W, Properdin deficiency (PFD);Three phenotypes have been reported: complete deficiency (type I), incomplete deficiency (type II), and dysfunction of properdin (type III); type II (UniProt), Properdin deficiency, type II (ClinVar), large intestine (Cosmic), Variant assessed as Somatic; impact. (NCI-TCGA), endometrium (Cosmic)**
**CA121391, RCV000011931, rs132630258, 161, R>*, Properdin deficiency, X-linked (ClinVar), Properdin deficiency, x-linked (cfpd) (Ensembl)**
**CA121401, RCV000011934, rs132630260, 206, S>*, Properdindeficiency, X-linked (ClinVar), Properdin deficiency, x-linked (cfpd) (Ensembl)**
**VAR 083039, 244, E>K, PFD; type II; decreases expression, inhibits oligomerization and fails to stimulate bacteriolysis; does not affect binding to Complement C3 beta chain (UniProt)**
**VAR 002004, CA121405, RCV000011935, rs132630261, 414, Y>D, PFD; type III; significantly decreases Complement C3 beta chain binding (UniProt), Properdin deficiency, type III (ClinVar)**

>sp|P08603|CFAH_HUMAN Complement factor H OS=Homo sapiens OX=9606 GN=CFH PE=1 SV=4
MRLLAKIICLMLWAICVAEDCNELPPRRNTEILTGSWSDQTYPEGTQAIYKCRPGYRSLG
NVIMVCRKGEWVALNPLRKCQK**RPCGHPGDT**PFGTFTLTGGNVFEYGVKAVYTCNEGYQL
LGEINYRECDTDGWTNDIPICEVVKCLPVTAPENGKIVSSAMEPDREYHFGQAVRFVCNS
GYKIEGDEEMHCSDDGFWSKEKPKCVEISCKSPDVINGSPISQKIIYKENERFQYKCNMG
YEYSERGDAVCTESGWRPLPSCEEKSCDNPYIPNGDYSPLRIKHRTGDEITYQC**RNGFYP**
**ATR**GNTAKCTSTGWI**PAPRCTLKP**CDY**PDIKHGGLYHENMRRPYFPVAVGKYYSYY**CDEH
FETPSGSYWDHIHCTQDGWSPAVP**CLRKCYFPYL**ENG**YNQNYGRKF**VQGKSIDVACHPGY
ALPKAQTTVTCMENGWSPTPRCI**RVKTCSKSS**IDIENGFISESQYTYALKEKAKYQCKLG
YVTADGETSGSITCGKDGWSAQPTCIKSCDIPVFMNARTKNDFTWFKLNDTLDYECHDGY
ESNTGSTTGSIVCGYNGWSDLPICYERECELPKIDVHLVPDRKKDQYKVGEVLKFSCKPG
FTIVGPNSVQCYHFGLSPDLPICKEQVQSCGPPPELLNGNVKEKTKEEYGHSEVVEYYCN
PRFLMKGPNKIQCVDGEWTTLPVCIVEESTCGDIPELEHGWAQLSSPPYYYGDSVEFNCS
ESF**TMIGHRSIT**CIHGVWTQLPQCVAID**KLKKCKSSN**L**IILEEHLKNK**KEFDHNSNIRYR
CRGKEGWIHTVCINGRWDPEVNCSMA**QIQLCPPPP**QIPNSHNMTTTLNYRDGEKVSVLCQ
ENYLIQEGEEITCKDGRWQ**SIPLCVEKI**PCSQPPQIEHGTINSSRSSQESYAHGTKLSYT
CEGGFRISEENETTCYMGKWSSPPQCEGLPCKSPPEISHGVVAHMSDSYQYGEEVTYKCF
EGFGIDGPAIAKCLG**EKWSHPPSCIKTDCLSLP**SFENAIPMGEKKDVYKAGEQVTYTCAT
YYKMDGASNVTCINSRWT**GRPTCRDTSCVNPP**T**VQNAYIVSR**QMSKYPSGERVRYQCRSP
YEMFGDEEVMCLNGNWTEPPQCKDSTGKCGPPPPIDNGDITSFPLSVYAPASSVEYQCQN
LYQLEGNKRITCRNGQWSEP**PKCLHPCVI**SREIMENYNIALRWTA**KQKLYSRTG**ESVEFV
CKRGYRL**SSRSHTLRTTCWDGK**LEYPTCAKR

**CA1305197, RCV001100290, RCV001100291, RCV001100292, RCV001100293, RCV001856363, rs142937931, 303, R>W, Basal laminar drusen (ClinVar), Variant assessed as Somatic; 0.0 impact. (NCI-TCGA), CFH-Related Dense Deposit**

Disease/Membranoproliferative Glomerulonephritis Type II (ClinVar), Age related macular degeneration 4 (ClinVar), Hemolytic uremic syndrome, atypical, susceptibility to, 1 (ClinVar)
VAR 031980, CA1305282, RCV001304040, RCV001332256, rs201671665, 400, Q>K, Hemolytic uremic syndrome atypical 1 (AHUS1) (UniProt), Factor H deficiency (ClinVar)
VAR 001979, RCV000018015, RCV000018016, rs1061170, 402, Y>H, associated with ARMD4 (UniProt), Basal laminar drusen (ClinVar), Age related macular degeneration 4 (ClinVar)
CA1305626, RCV001097067, RCV001097068, RCV001097069, RCV001097070, rs772553879, 760, I>L, Basal laminar drusen (ClinVar), CFH-Related Dense Deposit Disease/Membranoproliferative Glomerulonephritis Type II (ClinVar), Hemolytic uremic syndrome, atypical, susceptibility to, 1 (ClinVar), Age related macular degeneration 4 (ClinVar)
CA1305665, RCV001098818, RCV001098819, RCV001098820, RCV001098821, rs752302466, 808, I>M, Basal laminar drusen (ClinVar), CFH-Related Dense Deposit Disease/Membranoproliferative Glomerulonephritis Type II (ClinVar), Hemolytic uremic syndrome, atypical, susceptibility to, 1 (ClinVar), Age related macular degeneration 4 (ClinVar)
VAR 025870, 978, W>C, Hemolytic uremic syndrome atypical 1 (AHUS1) (UniProt)
CA1305781, RCV001097264, RCV001097265, RCV001100998, RCV001100999, rs149938052, 982, P>S, Basal laminar drusen (ClinVar), CFH-Related Dense Deposit Disease/Membranoproliferative Glomerulonephritis Type II (ClinVar), Hemolytic uremic syndrome, atypical, susceptibility to, 1 (ClinVar), Age related macular degeneration 4 (ClinVar)
CA10588915, RCV000256395, rs886039869, 984, C>R, Hemolytic uremic syndrome, atypical, susceptibility to, 1 (ClinVar)
VAR 025872, 1043, C>R, Hemolytic uremic syndrome atypical 1 (AHUS1) (UniProt)
CA1305867, COSM146717, RCV000266613, RCV000324131, RCV000359462, RCV000362490, RCV001701841, rs55679475, 1058, Y>H, Basal laminar drusen (ClinVar), CFH-Related Dense Deposit Disease/Membranoproliferative Glomerulonephritis Type II (ClinVar), stomach (Cosmic), Hemolytic uremic syndrome, atypical, susceptibility to, 1 (ClinVar), Age related macular degeneration 4 (ClinVar)
CA1305869, COSM146718, RCV000296097, RCV000330790, RCV000334829, RCV000388133, RCV001701937, rs55771831, 1060, V>L, Basal laminar drusen (ClinVar), CFH-Related Dense Deposit Disease/Membranoproliferative Glomerulonephritis Type II (ClinVar), stomach (Cosmic), Age related macular degeneration 4 (ClinVar), Hemolytic uremic syndrome, atypical, susceptibility to, 1 (ClinVar)
VAR 025878, 1163, C>W, Hemolytic uremic syndrome atypical 1 (AHUS1) (UniProt)
VAR 063650, 1169, I>L, Hemolytic uremic syndrome atypical 1 (AHUS1) (UniProt)
VAR 019407, CA257503, RCV000018014, rs121913055, 1189, L>R, Hemolytic uremic syndrome atypical 1 (AHUS1) (UniProt), Hemolytic uremic syndrome, atypical, susceptibility to, 1

**(ClinVar), Hemolytic uremic syndrome, atypical, susceptibility to, 1 (ahus1) (Ensembl)**
**VAR 019408, CA341428, rs460897, 1191, S>L, Hemolytic uremic syndrome atypical 1 (AHUS1) (UniProt), Hemolytic uremic syndrome, atypical, susceptibility to, 1 (ahus1) (Ensembl)**
**VAR 025882, CA1305983, RCV000788876, rs761877050, 1194, G>D, Hemolytic uremic syndrome atypical 1 (AHUS1) (UniProt)**
**VAR 025885, CA128563, rs121913059, 1210, R>C, Complement factor H deficiency (CFHD) and Macular degeneration, age-related, 4 (ARMD4); rare penetrant mutation that confers high risk of age-related macular degeneration (UniProt), Hemolytic uremic syndrome, atypical, susceptibility to, 1 (ahus1) (Ensembl)**
**CA1305996, COSM901548, RCV000276556, rs121913051, 1215, R>*, CFH-Related Disorders (ClinVar), Variant assessed as Somatic; 0.0 impact. (NCI-TCGA), endometrium (Cosmic), Hemolytic uremic syndrome, atypical, susceptibility to, 1 (ahus1) (Ensembl)**
**VAR 025886, CA257490, RCV000018008, RCV001328244, rs121913051, 1215, R>G, Hemolytic uremic syndrome atypical 1 (AHUS1) (UniProt), Atypical hemolytic-uremic syndrome (ClinVar), Hemolytic uremic syndrome, atypical, susceptibility to, 1 (ClinVar), Hemolytic uremic syndrome, atypical, susceptibility to, 1 (ahus1) (Ensembl)**
**VAR_025887, 1215, R>Q, Complement factor H deficiency (CFHD) (UniProt)**

>sp|P08603-2|CFAH_HUMAN Isoform 2 of Complement factor H OS=Homo sapiens OX=9606 GN=CFH
MRLLAKIICLMLWAICVAEDCNELPPRRNTEILTGSWSDQTYPEGTQAIYKCRPGYRSLG
NVIMVCRKGEWVALNPLRKCQK**RPCGHPGDT**PFGTFTLTGGNVFEYGVKAVYTCNEGYQL
LGEINYRECDTDGWTNDIPICEVVKCLPVTAPENGKIVSSAMEPDREYHFGQAVRFVCNS
GYKIEGDEEMHCSDDGFWSKEKPKCVEISCKSPDVINGSPISQKIIYKENERFQYKCNMG
YEYSERGDAVCTESGWRPLPSCEEKSCDNPYIPNGDYSPLRIKHRTGDEITYQC**RNGFYP**
**ATR**GNTAKCTSTGWI**PAPRCTLKP**CDY**PDIKHGGLY**HEN**MRRPYFPVAVGKYYSYY**CDEH
FETPSG**SYWDHIHCT**QDGWS**PAVPCLRKCYFPYL**ENG**YNQNYGRKF**VQGKSIDVACHPGY
ALPKAQTTVTCMENGWSPTPRCIRVSFTL

**CA1305197, RCV001100290, RCV001100291, RCV001100292, RCV001100293, RCV001856363, rs142937931, 303, R>W, Basal laminar drusen (ClinVar), Variant assessed as Somatic; 0.0 impact. (NCI-TCGA), CFH-Related Dense Deposit Disease/Membranoproliferative Glomerulonephritis Type II (ClinVar), Age related macular degeneration 4 (ClinVar), Hemolytic uremic syndrome, atypical, susceptibility to, 1 (ClinVar) [By similarity]**
**VAR 031980, CA1305282, RCV001304040, RCV001332256, rs201671665, 400, Q>K, Hemolytic uremic syndrome atypical 1 (AHUS1) (UniProt), Factor H deficiency (ClinVar) [By similarity]**
**VAR 001979, RCV000018015, RCV000018016, rs1061170, 402, Y>H, associated with ARMD4 (UniProt), Basal laminar drusen (ClinVar), Age related macular degeneration 4 (ClinVar) [By similarity]**

>sp|P15529|MCP_HUMAN Membrane cofactor protein OS=Homo sapiens OX=9606 GN=CD46 PE=1 SV=3

MEPPGRRECPFPSWRFPGLLLAAMVLLLYSFSDACEEPPTFEAMELIG**KPKPYYEIG**ERV
DYKCK**KGYFYIPPL**ATHTI**CDRNHTWLP**VSDDACYRETCPYIRDPLNGQAVPANGTYEFG
YQMHFICNEGYYLIGEEILYCELKGSVAIWSGKPPICEKVLCTPPPKIKNGKHTFSEVEV
FEYLDAVTYSCDPAPGPDPFSLIGESTIYCGDNSVWSRAAPECKVVKCRFPVVENGKQIS
GF**GKKFYYKAT**VMFECDKGFYLDGSDTIVCDSNSTWDPPVPKCLKVLPPSSTKPPALSHS
VSTSSTTKSPASSASG**PRPTYKPPV**SNYPGYPKPEEGILDSLDVWVIAVIVIAIVVGVAV
ICVVPYRYLQRRKKKGTYLTDETHREVKFTSL

**CA1371561, COSM1295809, RCV001099808, rs761000846, 82, R>Q, urinary_tract (Cosmic), Atypical hemolytic-uremic syndrome with MCP/CD46 anomaly (ClinVar), Variant assessed as Somatic; 9.285e-05 impact. (NCI-TCGA)**
**VAR_022263, CA1371821, RCV001096379, RCV001521407, rs41317833, 324, P>L, Atypical hemolytic-uremic syndrome with MCP/CD46 anomaly (ClinVar)**

>sp|P15529-2|MCP_HUMAN Isoform B of Membrane cofactor protein OS=Homo sapiens OX=9606 GN=CD46
MEPPGRRECPFPSWRFPGLLLAAMVLLLYSFSDACEEPPTFEAMELIG**KPKPYYEIG**ERV
DYKCK**KGYFYIPPL**ATHTI**CDRNHTWLP**VSDDACYRETCPYIRDPLNGQAVPANGTYEFG
YQMHFICNEGYYLIGEEILYCELKGSVAIWSGKPPICEKVLCTPPPKIKNGKHTFSEVEV
FEYLDAVTYSCDPAPGPDPFSLIGESTIYCGDNSVWSRAAPECKVVKCRFPVVENGKQIS
GF**GKKFYYKAT**VMFECDKGFYLDGSDTIVCDSNSTWDPPVPKCLKVLPPSSTKPPALSHS
VSTSSTTKSPASSASGPRPTYKPPVSNYPGYPKPEEGILDSLDVWVIAVIVIAIVVGVAV
ICVVPYRYLQRRKKKGKADGGAEYATYQTKSTTPAEQRG

**CA1371561, COSM1295809, RCV001099808, rs761000846, 82, R>Q, urinary_tract (Cosmic), Atypical hemolytic-uremic syndrome with MCP/CD46 anomaly (ClinVar), Variant assessed as Somatic; 9.285e-05 impact. (NCI-TCGA) [By similarity]**

>sp|P15529-11|MCP_HUMAN Isoform C of Membrane cofactor protein OS=Homo sapiens OX=9606 GN=CD46
MEPPGRRECPFPSWRFPGLLLAAMVLLLYSFSDACEEPPTFEAMELIG**KPKPYYEIG**ERV
DYKCK**KGYFYIPPL**ATHTI**CDRNHTWLP**VSDDACYRETCPYIRDPLNGQAVPANGTYEFG
YQMHFICNEGYYLIGEEILYCELKGSVAIWSGKPPICEKVLCTPPPKIKNGKHTFSEVEV
FEYLDAVTYSCDPAPGPDPFSLIGESTIYCGDNSVWSRAAPECKVVKCRFPVVENGKQIS
GF**GKKFYYKAT**VMFECDKGFYLDGSDTIVCDSNSTWDPPVPKCLKVSTSSTTKSPASSAS
G**PRPTYKPPV**SNYPGYPKPEEGILDSLDVWVIAVIVIAIVVGVAVICVVPYRYLQRRKKK
GTYLTDETHREVKFTSL

**CA1371561, COSM1295809, RCV001099808, rs761000846, 82, R>Q, urinary_tract (Cosmic), Atypical hemolytic-uremic syndrome with MCP/CD46 anomaly (ClinVar), Variant assessed as Somatic; 9.285e-05 impact. (NCI-TCGA) [By similarity]**
**VAR_022263, CA1371821, RCV001096379, RCV001521407, rs41317833, 309, P>L, Atypical hemolytic-uremic syndrome with MCP/CD46 anomaly (ClinVar) [By similarity]**

>sp|P15529-3|MCP_HUMAN Isoform D of Membrane cofactor protein OS=Homo sapiens OX=9606 GN=CD46
MEPPGRRECPFPSWRFPGLLLAAMVLLLYSFSDACEEPPTFEAMELIG**KPKPYYEIG**ERV
DYKCK**KGYFYIPPL**ATHTI**CDRNHTWLP**VSDDACYRETCPYIRDPLNGQAVPANGTYEFG
YQMHFICNEGYYLIGEEILYCELKGSVAIWSGKPPICEKVLCTPPPKIKNGKHTFSEVEV
FEYLDAVTYSCDPAPGPDPFSLIGESTIYCGDNSVWSRAAPECKVVKCRFPVVENGKQIS
GF**GKKFYYKAT**VMFECDKGFYLDGSDTIVCDSNSTWDPPVPKCLKVSTSSTTKSPASSAS

G**PRPTYKPPV**SNYPGYPKPEEGILDSLDVWVIAVIVIAIVVGVAVICVVPYRYLQRRKKK
GKADGGAEYATYQTKSTTPAEQRG

**CA1371561, COSM1295809, RCV001099808, rs761000846, 82, R>Q, urinary_tract (Cosmic), Atypical hemolytic-uremic syndrome with MCP/CD46 anomaly (ClinVar), Variant assessed as Somatic; 9.285e-05 impact. (NCI-TCGA) [By similarity]**
**VAR_022263, CA1371821, RCV001096379, RCV001521407, rs41317833, 309, P>L, Atypical hemolytic-uremic syndrome with MCP/CD46 anomaly (ClinVar) [By similarity]**

>sp|P15529-12|MCP_HUMAN Isoform E of Membrane cofactor protein OS=Homo sapiens OX=9606 GN=CD46
MEPPGRRECPFFPSWRFPGLLLAAMVLLLYSFSDACEEPPTFEAMELIG**KPKPYYEIG**ERV
DYKCK**KGYFYIPPL**ATHTI**CDRNHTWLP**VSDDACYRETCPYIRDPLNGQAVPANGTYEFG
YQMHFICNEGYYLIGEEILYCELKGSVAIWSG**KPPICEKVL**CTPPPKIKNGKHTFSEVEV
FEYLDAVTYSCDPAPGPDPFSLIGESTIYCGDNSVWSRAAPECKVVKCRFPVVENGKQIS
GF**GKKFYYKAT**VMFECDKGFYLDGSDTIVCDSNSTWDPPVPKCLKG**PRPTYKPPV**SNYPG
YPKPEEGILDSLDVWVIAVIVIAIVVGVAVICVVPYRYLQRRKKKGTYLTDETHREVKFT
SL

**CA1371561, COSM1295809, RCV001099808, rs761000846, 82, R>Q, urinary_tract (Cosmic), Atypical hemolytic-uremic syndrome with MCP/CD46 anomaly (ClinVar), Variant assessed as Somatic; 9.285e-05 impact. (NCI-TCGA) [By similarity]**
**VAR_022263, CA1371821, RCV001096379, RCV001521407, rs41317833, 294, P>L, Atypical hemolytic-uremic syndrome with MCP/CD46 anomaly (ClinVar) [By similarity]**

>sp|P15529-4|MCP_HUMAN Isoform F of Membrane cofactor protein OS=Homo sapiens OX=9606 GN=CD46
MEPPGRRECPFFPSWRFPGLLLAAMVLLLYSFSDACEEPPTFEAMELIG**KPKPYYEIG**ERV
DYKCK**KGYFYIPPL**ATHTI**CDRNHTWLP**VSDDACYRETCPYIRDPLNGQAVPANGTYEFG
YQMHFICNEGYYLIGEEILYCELKGSVAIWSG**KPPICEKVL**CTPPPKIKNGKHTFSEVEV
FEYLDAVTYSCDPAPGPDPFSLIGESTIYCGDNSVWSRAAPECKVVKCRFPVVENGKQIS
GF**GKKFYYKAT**VMFECDKGFYLDGSDTIVCDSNSTWDPPVPKCLKG**PRPTYKPPV**SNYPG
YPKPEEGILDSLDVWVIAVIVIAIVVGVAVICVVPYRYLQRRKKKGKADGGAEYATYQTK
STTPAEQRG

**CA1371561, COSM1295809, RCV001099808, rs761000846, 82, R>Q, urinary_tract (Cosmic), Atypical hemolytic-uremic syndrome with MCP/CD46 anomaly (ClinVar), Variant assessed as Somatic; 9.285e-05 impact. (NCI-TCGA) [By similarity]**
**VAR_022263, CA1371821, RCV001096379, RCV001521407, rs41317833, 294, P>L, Atypical hemolytic-uremic syndrome with MCP/CD46 anomaly (ClinVar) [By similarity]**

>sp|P15529-13|MCP_HUMAN Isoform G of Membrane cofactor protein OS=Homo sapiens OX=9606 GN=CD46
MEPPGRRECPFFPSWRFPGLLLAAMVLLLYSFSDACEEPPTFEAMELIG**KPKPYYEIG**ERV
DYKCK**KGYFYIPPL**ATHTI**CDRNHTWLP**VSDDACYRETCPYIRDPLNGQAVPANGTYEFG
YQMHFICNEGYYLIGEEILYCELKGSVAIWSGKPPICEKVLCTPPPKIKNGKHTFSEVEV
FEYLDAVTYSCDPAPGPDPFSLIGESTIYCGDNSVWSRAAPECKVVKCRFPVVENGKQIS
GF**GKKFYYKAT**VMFECDKGFYLDGSDTIVCDSNSTWDPPVPKCLKVLPPSSTKPPALSHS
VSTSSTTKSPASSASGYPKPEEGILDSLDVWVIAVIVIAIVVGVAVICVVPYRYLQRRKK
KGTYLTDETHREVKFTSL

**CA1371561, COSM1295809, RCV001099808, rs761000846, 82, R>Q, urinary_tract (Cosmic), Atypical hemolytic-uremic syndrome with MCP/CD46 anomaly (ClinVar), Variant assessed as Somatic; 9.285e-05 impact. (NCI-TCGA) [By similarity]**

>sp|P15529-5|MCP_HUMAN Isoform H of Membrane cofactor protein OS=Homo sapiens OX=9606 GN=CD46
MEPPGRRECPFPSWRFPGLLLAAMVLLLYSFSDACEEPPTFEAMELIG**KPKPYYEIG**ERV
DYKCK**KGYFYIPPL**ATHTI**CDRNHTWLP**VSDDACYRETCPYIRDPLNGQAVPANGTYEFG
YQMHFICNEGYYLIGEEILYCELKGSVAIWSGKPPICEKVLCTPPPKIKNGKHTFSEVEV
FEYLDAVTYSCDPAPGPDPFSLIGESTIYCGDNSVWSRAAPECKVVKCRFPVVENGKQIS
GF**GKKFYYKATV**MFECDKGFYLDGSDTIVCDSNSTWDPPVPKCLKVLPPSSTKPPALSHS
VSTSSTTKSPASSASGYPKPEEGILDSLDVWVIAVIVIAIVVGVAVICVVPYRYLQRRKK
KGKADGGAEYATYQTKSTTPAEQRG

**CA1371561, COSM1295809, RCV001099808, rs761000846, 82, R>Q, urinary_tract (Cosmic), Atypical hemolytic-uremic syndrome with MCP/CD46 anomaly (ClinVar), Variant assessed as Somatic; 9.285e-05 impact. (NCI-TCGA) [By similarity]**

>sp|P15529-14|MCP_HUMAN Isoform I of Membrane cofactor protein OS=Homo sapiens OX=9606 GN=CD46
MEPPGRRECPFPSWRFPGLLLAAMVLLLYSFSDACEEPPTFEAMELIG**KPKPYYEIG**ERV
DYKCK**KGYFYIPPL**ATHTI**CDRNHTWLP**VSDDACYRETCPYIRDPLNGQAVPANGTYEFG
YQMHFICNEGYYLIGEEILYCELKGSVAIWSGKPPICEKVLCTPPPKIKNGKHTFSEVEV
FEYLDAVTYSCDPAPGPDPFSLIGESTIYCGDNSVWSRAAPECKVVKCRFPVVENGKQIS
GF**GKKFYYKAT**VMFECDKGFYLDGSDTIVCDSNSTWDPPVPKCLKVSTSSTTKSPASSAS
GYPKPEEGILDSLDVWVIAVIVIAIVVGVAVICVVPYRYLQRRKKKGTYLTDETHREVKF
TSL

**CA1371561, COSM1295809, RCV001099808, rs761000846, 82, R>Q, urinary_tract (Cosmic), Atypical hemolytic-uremic syndrome with MCP/CD46 anomaly (ClinVar), Variant assessed as Somatic; 9.285e-05 impact. (NCI-TCGA) [By similarity]**

>sp|P15529-6|MCP_HUMAN Isoform J of Membrane cofactor protein OS=Homo sapiens OX=9606 GN=CD46
MEPPGRRECPFPSWRFPGLLLAAMVLLLYSFSDACEEPPTFEAMELIG**KPKPYYEIG**ERV
DYKCK**KGYFYIPPL**ATHTI**CDRNHTWLP**VSDDACYRETCPYIRDPLNGQAVPANGTYEFG
YQMHFICNEGYYLIGEEILYCELKGSVAIWSGKPPICEKVLCTPPPKIKNGKHTFSEVEV
FEYLDAVTYSCDPAPGPDPFSLIGESTIYCGDNSVWSRAAPECKVVKCRFPVVENGKQIS
GF**GKKFYYKAT**VMFECDKGFYLDGSDTIVCDSNSTWDPPVPKCLKVSTSSTTKSPASSAS
GYPKPEEGILDSLDVWVIAVIVIAIVVGVAVICVVPYRYLQRRKKKGKADGGAEYATYQT
KSTTPAEQRG

**CA1371561, COSM1295809, RCV001099808, rs761000846, 82, R>Q, urinary_tract (Cosmic), Atypical hemolytic-uremic syndrome with MCP/CD46 anomaly (ClinVar), Variant assessed as Somatic; 9.285e-05 impact. (NCI-TCGA) [By similarity]**

>sp|P15529-15|MCP_HUMAN Isoform K of Membrane cofactor protein OS=Homo sapiens OX=9606 GN=CD46
MEPPGRRECPFPSWRFPGLLLAAMVLLLYSFSDACEEPPTFEAMELIG**KPKPYYEIG**ERV
DYKCK**KGYFYIPPL**ATHTI**CDRNHTWLP**VSDDACYRETCPYIRDPLNGQAVPANGTYEFG
YQMHFICNEGYYLIGEEILYCELKGSVAIWSG**KPPICEKVL**CTPPPKIKNGKHTFSEVEV

FEYLDAVTYSCDPAPGPDPFSLIGESTIYCGDNSVWSRAAPECKVVKCRFPVVENGKQIS
GF**GKKFYYKATV**MFECDKGFYLDGSDTIVCDSNSTWDPPVPK**CLKGYPKPE**EGILDSLDV
WVIAVIVIAIVVGVAVICVVPYRYLQRRKKKGTYLTDETHREVKFTSL

**CA1371561, COSM1295809, RCV001099808, rs761000846, 82, R>Q, urinary_tract (Cosmic), Atypical hemolytic-uremic syndrome with MCP/CD46 anomaly (ClinVar), Variant assessed as Somatic; 9.285e-05 impact. (NCI-TCGA) [By similarity]**
**CA10609582, RCV000342161, rs886045838, 288, P>A, Atypical hemolytic-uremic syndrome with MCP/CD46 anomaly (ClinVar)**

>sp|P15529-7|MCP_HUMAN Isoform L of Membrane cofactor protein OS=Homo sapiens OX=9606 GN=CD46
MEPPGRRECPFPSWRFPGLLLAAMVLLLYSFSDACEEPPTFEAMELIG**KPKPYYEIG**ERV
DYKCK**KGYFYIPPL**ATHTI**CDRNHTWLP**VSDDACYRETCPYIRDPLNGQAVPANGTYEFG
YQMHFICNEGYYLIGEEILYCELKGSVAIWSG**KPPICEKVL**CTPPPKIKNGKHTFSEVEV
FEYLDAVTYSCDPAPGPDPFSLIGESTIYCGDNSVWSRAAPECKVVKCRFPVVENGKQIS
GF**GKKFYYKATV**MFECDKGFYLDGSDTIVCDSNSTWDPPVPK**CLKGYPKPE**EGILDSLDV
WVIAVIVIAIVVGVAVICVVPYRYLQRRKKKGKADGGAEYATYQTKSTTPAEQRG

**CA1371561, COSM1295809, RCV001099808, rs761000846, 82, R>Q, urinary_tract (Cosmic), Atypical hemolytic-uremic syndrome with MCP/CD46 anomaly (ClinVar), Variant assessed as Somatic; 9.285e-05 impact. (NCI-TCGA) [By similarity]**
**CA10609582, RCV000342161, rs886045838, 288, P>A, Atypical hemolytic-uremic syndrome with MCP/CD46 anomaly (ClinVar)**

>sp|P15529-8|MCP_HUMAN Isoform M of Membrane cofactor protein OS=Homo sapiens OX=9606 GN=CD46
MEPPGRRECPFPSWRFPGLLLAAMVLLLYSFSDACEEPPTFEAMELIG**KPKPYYEIG**ERV
DYKCK**KGYFYIPPL**ATHTI**CDRNHTWLP**VSDDACYRETCPYIRDPLNGQAVPANGTYEFG
YQMHFICNEGYYLIGEEILYCELKGSVAIWSGKPPICEKVLCTPPPKIKNGKHTFSEVEV
FEYLDAVTYSCDPAPGPDPFSLIGESTIYCGDNSVWSRAAPECKVVKCRFPVVENGKQIS
GF**GKKFYYKAT**VMFECDKGFYLDGSDTIVCDSNSTWDPPVPKCLKVSTSSTTKSPASSAS
G**PRPTYKPPV**SNYPGYPKPEEGILDSLDVWVIAVIVIAIGKQMVELNMPLTRLNQPLQQS
REAE

**CA1371561, COSM1295809, RCV001099808, rs761000846, 82, R>Q, urinary_tract (Cosmic), Atypical hemolytic-uremic syndrome with MCP/CD46 anomaly (ClinVar), Variant assessed as Somatic; 9.285e-05 impact. (NCI-TCGA) [By similarity]**
**VAR_022263, CA1371821, RCV001096379, RCV001521407, rs41317833, 309, P>L, Atypical hemolytic-uremic syndrome with MCP/CD46 anomaly (ClinVar)**

>sp|P15529-9|MCP_HUMAN Isoform N of Membrane cofactor protein OS=Homo sapiens OX=9606 GN=CD46
MEPPGRRECPFPSWRFPGLLLAAMVLLLYSFSDACEEPPTFEAMELIG**KPKPYYEIG**ERV
DYKCKKGYFYIPPLATHTI**CDRNHTWLP**VSDDACYRETCPYIRDPLNGQAVPANGTYEFG
YQMHFICNEGYYLIGEEILYCELKGSVAIWSG**KPPICEKVL**CTPPPKIKNGKHTFSEVEV
FEYLDAVTYSCDPAPGPDPFSLIGESTIYCGDNSVWSRAAPECKVVKCRFPVVENGKQIS
GF**GKKFYYKATV**MFECDKGFYLDGSDTIVCDSNSTWDPPVPKCLKG**PRPTYKPPV**SNYPG
YPKPEEGILDSLDVWVIAVIVIAIGKQMVELNMPLTRLNQPLQQSREAE

**CA1371561, COSM1295809, RCV001099808, rs761000846, 82, R>Q, urinary_tract (Cosmic), Atypical hemolytic-uremic syndrome**

**with MCP/CD46 anomaly (ClinVar), Variant assessed as Somatic; 9.285e-05 impact. (NCI-TCGA) [By similarity]**
**VAR_022263, CA1371821, RCV001096379, RCV001521407, rs41317833, 294, P>L, Atypical hemolytic-uremic syndrome with MCP/CD46 anomaly (ClinVar)**

>sp|P15529-10|MCP_HUMAN Isoform 2 of Membrane cofactor protein OS=Homo sapiens OX=9606 GN=CD46
MEPPGRRECPFPSWRFPGLLLAAMVLLLYSFSDACEEPPTFEAMELIG**KPKPYYEIG**ERV
DYKCK**KGYFYIPPL**ATHTI**CDRNHTWLP**VSDDACYRETCPYIRDPLNGQAVPANGTYEFG
YQMHFICNEGYYLIGEEILYCELKGSVAIWSGKPPICEKVLCTPPPKIKNGKHTFSEVEV
FEYLDAVTYSCDPAPGPDPFSLIGESTIYCGDNSVWSRAAPECKVVKCRFPVVENGKQIS
GF**GKKFYYKAT**VMFECDKGFYLDGSDTIVCDSNSTWDPPVPKCLKVLPPSSTKP**PALSHS**
**TTK**SPASSASGPRPTYKPPVSNYPGYPKPEEGILDSLDVWVIAVIVIAIVVGVAVICVVP
YRYLQRRKKKGTYLTDETHREVKFTSL

**CA1371561, COSM1295809, RCV001099808, rs761000846, 82, R>Q, urinary_tract (Cosmic), Atypical hemolytic-uremic syndrome with MCP/CD46 anomaly (ClinVar), Variant assessed as Somatic; 9.285e-05 impact. (NCI-TCGA) [By similarity]**

>sp|P17927|CR1_HUMAN Complement receptor type 1 OS=Homo sapiens OX=9606 GN=CR1 PE=1 SV=3 [Isoform F/CR1-A/CR1*1)
MGASSPRSPEPVGPPAPGLPFCCGGSLLAVVVLLALPVAWGQCNAPEWLPFARPTNLTDE
FEFPIGTYLNYECRPGYSGRPFSIICLKNSVWTGAKDRCRRKSCRNPPDPVNGMVHVIKG
IQFGSQIKYSCTKGYRLIGSSSATCIISGDTVIWDNETPICDRIPCGLPPTITNGDFIST
NRENFHYGSVVTYRCNPGSGGRKVFELVGEPSIYCTSNDDQVGIWSGPAPQCIIPNKCTP
PNVENGILVSDNRSLFSLNEVVEFRCQPGFVMKGPRRVKCQALNKWEPELPSCSRVCQPP
PDVLHAERTQRDKDNFSPGQEVFYSCEPGYDLRGAASMRCTPQGDWSPAAPTCEVKSCDD
FMGQLLNGRVLFPVNLQLGAKVDFVCDEGFQLKGSSASYCVLAGMESLWNSSVPVCEQIF
CPSPPVIPNGRHTGKPLEVFPFGKTVNYTCDPHPDRGTSFDLIGESTIRCTSDPQGNGVW
SSPAPRCGILGHCQAPDHFLFAKLKTQTNASDFPIGTSLKYEC**RPEYYGRPF**SITCLDNL
VWSSPKDVCKRKSCKTPPDPVNGMVHVITDIQVGSRINYSCTTGH**RLIGHSSAE**CILS**GN**
**AAHWST**KPPICQRIPCGLPPTIANGDFISTN**RENFHYGSV**VTYRCNPGSGGRKVFELVGE
PSIYCTSNDDQVGIWSG**PAPQCIIPN**KCTPPNVENGILVSDNRSLFSLNEVVEFRCQPGF
VMKGPRRVKCQALNKWEPELPS**CSRVCQPPP**DVLHAERTQRDKDNFSPGQEVFYSCEPGY
DLRGAASMRCTPQGDWSPAAPTCEVKSCDDFMGQLLNGRVLFPVNLQLGAKVDFVCDEGF
QLKGSSASYCVLAGMESLWNSSVPVC**EQIFCPSPP**VIPNGRHTGKPLEVFPFGKAVNYTC
DPHPDRGTSFDLIGESTIRCTSDPQGNGVWSSPAPRCGILGHCQAPDHFLFAKLKTQTNA
SDFPIGTSLKYEC**RPEYYGRPF**SITCLDNLVWSS**PKDVCKRKS**CKTPPDPVNGMVHVITD
IQVGSRINYSCTTGH**RLIGHSSAE**CILS**GNTAHWSTK**PPICQRIPCGLPPTIANGDFIST
N**RENFHYGSV**VTYRCNLGSRGRKVFELVGEPSIYCTSNDDQVGIWSG**PAPQCIIPN**KCTP
PNVENGILVSDNRSLFSLNEVVEFRCQPGFVMKGPRRVKCQALNKWEPELPS**CSRVCQPP**
**P**EILHGE**HTPSHQDNF**SPGQEVFYSCEPGYDLRGAASLHCTPQGDWSP**EAPRCAVKS**CDD
FL**GQLPHGRVL**FPLNLQLGAKVSFVCDEGFRLKGSSVSHCVLVGMRSLWNNSVPVCEHIF
CPNPPAILNGRHTGTPSGDIPYGKEISYTCDPHPDRGMTFNLIGESTIRCTSDPHGNGVW
SSPAPRCELS**VRAGHCKTP**EQFPFASPTIPINDFEFPVGTSLNYECRPGYFGKMFSISCL
ENLVWSSVEDNCRRKSCGPPPEPFNGMVHINTDTQFGSTVNYSCNEGFRLIGSPSTTCLV
SGNNVTWDKKAPICEIISCEPPPTISNGDFYSNN**RTSFHNGTV**VTYQCHTGPDGEQLFEL
VGERSIYCTSKDDQVGVWSSPPPRCISTNKCTAPEVENAIRVPGNRSFFSLTEIIRFRCQ
PGFVMVGSHTVQCQTNGRWG**PKLPHCSRV**CQPPPEILHGE**HTLSHQDNF**SPGQEVFYSCE
PSYDLRGAASLHCTPQGDWSPEAPRCTVKSCDDFL**GQLPHGRVL**LPLNLQLGAKVSFVCD
EGFRLKG**RSASHCVLA**GMKALWNSSVPVCEQIFCPNPPAILNGRHTGTPFGDIPYGKEIS
YACDTHPDRGMTFNLIGESSIRCTSDPQGNGVWSSPAPRCELSVP**AACPHPPKI**QNGHYI
GGHVSLYLPGMTISYICDPGYLLVGKGFIFCTDQGIWSQLDHYCKEVNCSFPLFMNGISK
ELEM**KKVYHYGDYVTLK**CEDGYTLEGSPWSQCQADDRWDPPLAKC**TSRTHD**ALIVGTLSG

TIFFILLIIFLSWIILKHRKGNNAHENPKEVAIHLHSQGGSSVHPRTLQTNEENSRVLP

**VAR_013819, CA1370009, RCV000018595, rs2274567, 1208, H>R, Malaria, severe, resistance to (ClinVar)**

>tr|E9PDY4|E9PDY4_HUMAN Complement receptor type 1 OS=Homo sapiens OX=9606 GN=CR1 PE=1 SV=1 [Isoform S/CR1-B/CR1*2]
MGASSPRSPEPVGPPAPGLPFCCGGSLLAVVVLLALPVAWGQCNAPEWLPFARPTNLTDE
FEFPIGTYLNYECRPGYSGRPFSIICLKNSVWTGAKDRCRRKSCRNPPDPVNGMVHVIKG
IQFGSQIKYSCTKGYRLIGSSSATCIISGDTVIWDNETPICDRIPCGLPPTITNGDFIST
NRENFHYGSVVTYRCNPGSGGRKVFELVGEPSIYCTSNDDQVGIWSGPAPQCIIPNKCTP
PNVENGILVSDNRSLFSLNEVVEFRCQPGFVMKGPRRVKCQALNKWEPELPSCSRVCQPP
PDVLHAERTQRDKDNFSPGQEVFYSCEPGYDLRGAASMRCTPQGDWSPAAPTCEVKSCDD
FMGQLLNGRVLFPVNLQLGAKVDFVCDEGFQLKGSSASYCVLAGMESLWNSSVPVCEQIF
CPSPPVIPNGRHTGKPLEVFPFGKTVNYTCDPHPDRGTSFDLIGESTIRCTSDPQGNGVW
SSPAPRCGILGHCQAPDHFLFAKLKTQTNASDFPIGTSLKYEC**RPEYYGRPF**SITCLDNL
VWSSPKDVCKRKSCKTPPDPVNGMVHVITDIQVGSRINYSCTTGH**RLIGHSSAE**CILS**GN**
**AAHWSTK**PPICQRIPCGLPPTIANGDFISTN**RENFHYGSV**VTYRCNPGSGGRKVFELVGE
PSIYCTSNDDQVGIWSGPAPQCIIPNKCTPPNVENGILVSDNRSLFSLNEVVEFRCQPGF
VMKGPRRVKCQALNKWEPELPS**CSRVCQPPP**DVLHAERTQRDKDNFSPGQEVFYSCEPGY
DLRGAASMRCTPQGDWSP**AAPTCEVKS**CDDFMGQLLNGRVLFPVNLQLGAKVDFVCDEGF
QLKGSSASYCVLAGMESLWNSSVPVC**EQIFCPSPP**VIPNGRHTGKPLEVFPFGKTVNYTC
DPHPDRGTSFDLIGESTIRCTSDPQGNGVWSSPAPRCGILGHCQAPDHFLFAKLKTQTNA
SDFPIGTSLKYEC**RPEYYGRPF**SITCLDNLVWSSPKDVCKRKSCKTPPDPVNGMVHVITD
IQVGSRINYSCTTGH**RLIGHSSAE**CILS**GNAAHWSTKPPICQRIPCGLPP**TIANGDFIST
N**RENFHYGSV**VTYRCNPGSGGRKVFELVGEPSIYCTSNDDQVGIWSGPAPQCIIPNKCTP
PNVENGILVSDNRSLFSLNEVVEFRCQPGFVMKGPRRVKCQALNKWEPELPS**CSRVCQPP**
**P**DVLHAERTQRDKDNFSPGQEVFYSCEPGYDLRGAASMRCTPQGDWSPAAPTCEVKSCDD
FMGQLLNGRVLFPVNLQLGAKVDFVCDEGFQLKGSSASYCVLAGMESLWNSSVPVCEQIF
CPSPPVIPNGRHTGKPLEVFPFGKAVNYTCDPHPDRGTSFDLIGESTIRCTSDPQGNGVW
SS**PAPRCGILGHCQAPD**HFLFAKLKTQTNASDFPIGTSLKYEC**RPEYYGRPF**SITCLDNL
VWSSPKDVCKRKSCKTPPDPVNGMVHVITDIQVGSRINYSCTTGH**RLIGHSSAE**CILS**GN**
**TAHWSTKPPICQRIP**CGLPPTIANGDFISTN**RENFHYGSV**VTYRCNLGSRGRKVFELVGE
PSIYCTSNDDQVGIWSG**PAPQCIIPN**KCTPPNVENGILVSDNRSLFSLNEVVEFRCQPGF
VMKGPRRVKCQALNKWEP**ELPSCSRVCQPPP**EILHGE**HTPSHQDNF**SPGQEVFYSCEPGY
DLRGAASLHCTPQGDWSPEAPRCAVKSCDDFL**GQLPHGRVL**FPLNLQLGAKVSFVCDEGF
RLKGSSVSHCVLVGMRSLWNNSVPVCEHIFCPNPPAILNGRHTGTPSGDIPYGKEISYTC
DPHPDRGMTFNLIGESTIRCTSDPHGNGVWSSPAPRCELS**VRAGHCKTP**EQFPFASPTIP
INDFEFPVGTSLNYECRPGYFGKMFSISCLENLVWSSVEDNCRRKSCGPPPEPFNGMVHI
NTDTQFGSTVNYSCNEGFRLIGSPSTTCLVSGNNVTWDK**KAPICEIIS**CEPPPTISNGDF
YSNN**RTSFHNGTV**VTYQCHTGPDGEQLFELVGERSIYCTSKDDQVGVWSSPPPRCISTNK
CTAPEVENAIRVPGNRSFFTLTEIIRFRCQPGFVMVGSHTVQCQTNGRWG**PKLPHCSRVC**
**QPPP**EILHGE**HTLSHQDNF**SPGQEVFYSCEPSYDLRGAASLHCTPQGDWSP**EAPRCTVKS**
CDDFL**GQLPHGRVL**LPLNLQLGAKVSFVCDEGFRLKGRSASHCVLAGMKALWNSSVPVCE
QIFCPNPPAILNGRHTGTPFGDIPYGKEISYACDTHPDRGMTFNLIGESSIRCTSDPQGN
GVWSSPAPRCELSVP**AACPHPPKI**QNGHYIGGHVSLYLPGMTISYICDPGYLLVGKGFIF
CTDQGIWSQLDHYCKEVNCSFPLFMNGISKELEM**KKVYHYGDYVTLK**CEDGYTLEGSPWS
QCQADDRWDPPLAKC**TSRTHD**ALIVGTLSGTIFFILLIIFLSWIILKHRKGNNAHENPKE
VAIHLHSQGGSSVHPRTLQTNEENSRVLP

**VAR_013819, CA1370009, RCV000018595, rs2274567, 1658, H>R, Malaria, severe, resistance to (ClinVar)[By similarity]**

>sp|Q02985|FHR3_HUMAN Complement factor H-related protein 3 OS=Homo sapiens OX=9606 GN=CFHR3 PE=1 SV=2
MLLLINVILTLWVSCANGQVKPCDF**PDIKHGGLF**HEN**MRRPYFPVAVGKYYSYY**CDEHFE

TPSGSYWDYIHCTQNGWSPAVPC**LRKCYFPYL**ENG**YNQNYGRKF**VQGNSTEVACHPGYGL
PKAQTTVTCTEKGWS**PTPRCIRVR**TCSKSDIEIENGFISESSSIYILNKEIQYKCKPGYA
TADGNSSGSITCLQNGWSAQPICINSSEKCGPPPPISNGDTTSFLLKVYVPQSRVEYQCQ
PYYELQGSNYVTCSNGEWS**EPPRCIHPCIITE**ENMNKNNIKLKGR**SDRKYYAKTG**DTIEF
MCKLGYNANTSILS**FQAVCREGI**VEYPRCE

**CA1306293, RCV000626208, rs745503234, 268, C>F, Age related macular degeneration 1 (ClinVar)**

>sp|Q02985-2|FHR3_HUMAN Isoform 2 of Complement factor H-related protein 3 OS=Homo sapiens OX=9606 GN=CFHR3
MLLLINVILTLWVSCANGQVKPCDF**PDIKHGGLF**HEN**MRRPYFPVAVGKYYSYY**CDEHFE
TPSGSYWDYIHCTQNGWSPAVPC**LRKCYFPYL**ENG**YNQNYGRKF**VQGNSTEVACHPGYGL
PKAQTTVTCTEKGWSPTPRCIRVNSSEKCGPPPPISNGDTTSFLLKVYVPQSRVEYQCQP
YYELQGSNYVTCSNGEWS**EPPRCIHPCII**TEENMNKNNIKLKGR**SDRKYYAKTG**DTIEFM
CKLGYNANTSILS**FQAVCREGI**VEYPRCE

**CA1306293, RCV000626208, rs745503234, 207, C>F, Age related macular degeneration 1 (ClinVar)**

>sp|Q9BXR6|FHR5_HUMAN Complement factor H-related protein 5 OS=Homo sapiens OX=9606 GN=CFHR5 PE=1 SV=1
MLLLFSVILISWVSTVGGEGTLCD**FPKIHHGFLY**DEEDYNPFSQVPTGEVFYYSCEYNFV
SPSKSFWTRITCTEEGWSPTPKCLRMCSFPF**VKNGHSESS**GLIHLEGDTVQIICNTGYSL
QNNEKNISCVERGWS**TPPICSFTKGECHVPIL**EANVDAQPKKESYKVGDVLKFSCRKNLI
RVGSDSVQCYQFGWSPNFPTCKGQVRSCGPPPQLSNGEVKEIRKEEYGHNEVVEYDCNPN
FIINGPKKIQCVDGEWTTLPTCVEQVKTCGYIPELEYGYVQPSVPPYQHGVSVEVNCRNE
YAMIGNNMITCINGIWTELPM**CVATHQLKR**CKIAGVNIKTLLKLSG**KEFNHNSRI**RYRCS
DIFRYRHSVCINGKWNPEVDCTEKREQFCPPPPQIPNAQNMTTTVNYQDGEKVAVLCKEN
YLLPEAKEIVCKDGRWQ**SLPRCVEST**AYCGPPPSINNGDTTSFPLSVYPPGSTVTYRCQS
FYKLQGSVTVTCRNKQWSEPPRCLDPCVVSEENMNKNNIQLKWRNDGKLYAKTGDAVEFQ
CKFPHKAMISSPPFRAICQEGKFEYPICE

**RCV001097650, rs1653577983, 26, P>S, CFH-Related Dense Deposit Disease/Membranoproliferative Glomerulonephritis Type II (ClinVar)**
**CA1307751, RCV001101405, RCV001819805, RCV002069699, rs181511327, 144, K>N, CFH-Related Dense Deposit Disease/Membranoproliferative Glomerulonephritis Type II (ClinVar)**

**Defects in CFHR5 have been found in patients with atypical hemolytic uremic syndrome and may contribute to the disease. Atypical hemolytic uremic syndrome is a complex genetic disease characterized by microangiopathic hemolytic anemia, thrombocytopenia, renal failure and absence of episodes of enterocolitis and diarrhea. In contrast to typical hemolytic uremic syndrome, atypical forms have a poorer prognosis, with higher death rates and frequent progression to end-stage renal disease. Susceptibility to the development of atypical hemolytic uremic syndrome can be conferred by mutations in various components of or regulatory factors in the complement cascade system. Other genes may play a role in modifying the phenotype.**

**10 unique entries/genes**

**28 sequences**

## Species with detected PTMs (PhosphoSitePlus)

>sp|P01024|CO3_HUMAN Complement C3 OS=Homo sapiens OX=9606 GN=C3 PE=1 SV=2
MGPTSGPSLLLLLLTHLPLALGSPMYSIITPNILRLESEETMVLEAHDAQGDVPVTVTVH
DFPGKKLVLSSEKTVLTPATNHMGNVTFTIPAN**R**EFKSEKG**R**N**K**FVTVQATFGTQVVEKV
VLVSLQSGYLFIQTDKTIYTPGSTVLYRI**FTVNHKLLP**VGRTVMVNIENPEGIPVKQDSL
SSQNQLGVLPLSWDIPELVNMGQW**KIRAYYENS**PQQVFSTEFEVKEYVLPSFEVIVEPTE
KFYYIYNEKGLEVTITARFLYGKKVEGTAFVIFGIQDGEQRISLPESLKRIPIEDG**S**GEV
VLSRKVLLDGVQN**P**RAEDLVGKSLYVSATVILHSGSDMVQAERSGIPIVTS**PYQIHFTKT**
PKYFKPGMPFDLMVFVTNPD**GSPAYRVPV**AVQGEDTVQSLTQGDGVAKL**SINTHPSQK**PL
SITVRTKKQELSEAEQATRTMQALPYSTVGNSNNYLHLSVLRTELRPGETLNVNFLLR**MD**
**RAHEAKI**RYYTYLIMNKGRLLKAGRQVREPGQDLVVLPLSITTDFIPSFRLVAYYTLIGA
SGQREVVADSVWVDVKDSCVGSLVVKSGQSEDRQPVPGQQMTLKIEGDHGARVVLVAVDK
GVFVLNKKNKLTQSKIWDVVEKADIGCTPGSGKDYAGVFSDAGLTFTSSSGQQTAQRAEL
QCPQPAARRRR**S**VQLTEKRMDKVGKYPKELRKCCEDGMRENPMRFSCQRRTRFISLGEAC
KKVFLDCCNYITE**LRRQHARASHLGLA**RSNLDEDIIAEENIVSRSEFPESWLWNVEDLKE
PPKNGISTKLMNIFLKDSITTWEILAVSMSDKKGICVADPFEVTVMQDFFIDLRLPYSVV
RNEQVEIRAVLYNYRQNQELKVRVELLH**NPAFCSLATTKRRHQQTV**TIPPKSSLSVPYVI
VPLKTGLQEVEV**KAAVYHHFISD**GVRKSLKVVPEGIRMNKTVAVRTLDPERLGREGVQKE
DIPPADLSDQVPDTESETRILLQGTPVAQMTEDAVDA**ERLKHLIVT**PSGCGEQNMIGMTP
TVIAVHYL**DE**T**E**QW**E**KFGLEKRQGALELIKKGYTQQLAFRQPSSAFAAFVKRAPSTWLTA
YVVKVFSLAVNLIAID**SQVLCGAVK**WLILEKQKPDGVFQEDAPVIHQEMIGGLRNNNEKD
MALTAFVLISLQEAKDICEEQVNSLPGSITKAGDFLEANYMNLQRSYTVAIAGYALAQMG
RLKGPLLNKFLTTAKDKNRWEDPGKQLYNVEATSYALLALLQLKDFDFVPPVVRWLNEQR
YYGGGYGSTQATFMVFQALAQYQKDAPDHQELNLDVSLQLPSRSSKI**THRIHWESA**SLLR
SEETKENEGFTVTAEGKGQGTLSVVTMYHAKAKDQLTCNKFDLKVTIKPAPETEKRPQDA
KNTMILEICTRYRGDQDATMSILDISMMTGFAPDTDDLKQLANGVDRYISKYELDKAFSD
RNTLIIYLDKVSHSEDDCL**AFKVHQYFNVE**LIQPGAVKVYAYYNLEESC**TRFYHPEKE**DG
KLNKLCRDELCRCAEENCFIQKSDDKVTLEERLDKACEPGVDYVYKTRLVKVQLSNDFDE
YIMAIEQTIKSGSDEVQVGQQRTFISPIKCREALKL**EEKKHYLMW**GLSSDFWGEKPNLSY
IIGKDTWVEHWPEEDECQDEENQKQCQDLGAFTESMVVFGCPN

**From PhosphositePlus (Minimum 5 references)**
**S297, Phosphorylation**
**S672, Phosphorylation**

>sp|P08603|CFAH_HUMAN Complement factor H OS=Homo sapiens OX=9606 GN=CFH PE=1 SV=4
MRLLAKIICLMLWAICVAEDCNELPPRRNTEILTGSWSDQTYPEGTQAIYKCRPGYRSLG
NVIMVCRKGEWVALNPLRKCQK**RPCGHPGDT**PFGTFTLTGGNVFEYGVKAVYTCNEGYQL
LGEINYRECDTDGWTNDIPICEVVKCLPVTAPENGKIVSSAMEPDREYHFGQAVRFVCNS
GYKIEGDEEMHCSDDGFWSKEKPKCVEISCKSPDVINGSPISQKIIYKENERFQYKCNMG
YEYSERGDAVCTESGWRPLPSCEEKSCDNPYIPNGDYSPLRIKHRTGDEITYQC**RNGFYP**
**ATR**GNTAKCTSTGWI**PAPRCTLKP**CDY**PDIKHGGLYHENMRRPYFPVAVGKYYSYY**CDEH
FETPSGSYWDHIHCTQDGWSPAVP**CLRKCYFPYL**ENG**YNQNYGRKF**VQGKSIDVACHPGY
ALPKAQTTVTCMENGWSPTPRCI**RVKTCSKSS**IDIENGFISESQYTYALKEKAKYQCKLG
YVTADGETSGSITCGKDGWSAQPTCIKSCDIPVFMNARTKNDFTWFKLNDTLDYECHDGY
ESNTGSTTGSIVCGYNGWSDLPICYERECELPKIDVHLVPDRKKDQYKVGEVLKFSCKPG
FTIVGPNSVQCYHFGLSPDLPICKEQVQSCGPPPELLNGNVKEKTKEEYGHSEVVEYYCN
PRFLMKGPNKIQCVDGEWTTLPVCIVEESTCGDIPELEHGWAQLSSPPYYYGDSVEFNCS
ESF**TMIGHRSIT**CIHGVWTQLPQCVAID**KLKKCKSSN**L**IILEEHLKNK**KEFDHNSNIRYR
CRGKEGWIHTVCINGRWDPEVNCSMA**QIQLCPPPP**QIPNSHNMTTTLNYRDGEKVSVLCQ
ENYLIQEGEEITCKDGRWQ**SIPLCVEKI**PCSQPPQIEHGTINSSRSSQESYAHGTKLSYT
CEGGFRISEENETTCYMGKWSSPPQCEGLPCKSPPEISHGVVAHMSDSYQYGEEVTYKCF

EGFGIDGPAIAKCLG**EKWSHPPSCIKTDCLSLP**SFENAIPMGEKKDVYKAGEQVTYTCAT
YYKMDGASNVTCINSRWT**GRPTCRDTSCVNPP**T**VQNAYIVSR**QMSKYPSGERVRYQCRSP
YEMFGDEEVMCLNGNWTEPPQCKDSTGKCGPPPPIDNGDITSFPLSVYAPASSVEYQCQN
LYQLEGNKRITCRNGQWSEP**PKCLHPCVI**SREIMENYNIALRWTA**KQKLYSR**T**G**ESVEFV
CKRGYRL**SSRSHTLRTTCWDGK**LEYPTCAKR

**From PhosphositePlus (Minimum 5 references)**
**T1193, Phosphorylation**

>sp|P15529|MCP_HUMAN Membrane cofactor protein OS=Homo sapiens OX=9606 GN=CD46 PE=1 SV=3
MEPPGRRECPFPSWRFPGLLLAAMVLLLYSFSDACEEPPTFEAMELIG**KPKPYYEIG**ERV
DYKCK**KGYFYIPPL**ATHTI**CDRNHTWLP**VSDDACYRETCPYIRDPLNGQAVPANGTYEFG
YQMHFICNEGYYLIGEEILYCELKGSVAIWSGKPPICEKVLCTPPPKIKNGKHTFSEVEV
FEYLDAVTYSCDPAPGPDPFSLIGESTIYCGDNSVWSRAAPECKVVKCRFPVVENGKQIS
GF**GKKFYYKAT**VMFECDKGFYLDGSDTIVCDSNSTWDPPVPKCLKVLPPSSTKPPALSHS
VSTSSTTKSPASSASG**PRPTYKPPV**SNYPGYPKPEEGILDSLDVWVIAVIVIAIVVGVAV
ICVVPYRYLQRRKK**K**GT**Y**LTDETHREVKFTSL

**From PhosphositePlus (Minimum 5 references)**
**K375, Ubiquitylation**
**Y378, Phosphorylation**

>sp|P15529-2|MCP_HUMAN Isoform B of Membrane cofactor protein OS=Homo sapiens OX=9606 GN=CD46
MEPPGRRECPFPSWRFPGLLLAAMVLLLYSFSDACEEPPTFEAMELIG**KPKPYYEIG**ERV
DYKCK**KGYFYIPPL**ATHTI**CDRNHTWLP**VSDDACYRETCPYIRDPLNGQAVPANGTYEFG
YQMHFICNEGYYLIGEEILYCELKGSVAIWSGKPPICEKVLCTPPPKIKNGKHTFSEVEV
FEYLDAVTYSCDPAPGPDPFSLIGESTIYCGDNSVWSRAAPECKVVKCRFPVVENGKQIS
GF**GKKFYYKAT**VMFECDKGFYLDGSDTIVCDSNSTWDPPVPKCLKVLPPSSTKPPALSHS
VSTSSTTKSPASSASGPRPTYKPPVSNYPGYPKPEEGILDSLDVWVIAVIVIAIVVGVAV
ICVVPYRYLQRRKK**K**GKADGGAEYATYQTKSTTPAEQRG

**From PhosphositePlus (Minimum 5 references)**
**K375, Ubiquitylation [By similarity]**

>sp|P15529-11|MCP_HUMAN Isoform C of Membrane cofactor protein OS=Homo sapiens OX=9606 GN=CD46
MEPPGRRECPFPSWRFPGLLLAAMVLLLYSFSDACEEPPTFEAMELIG**KPKPYYEIG**ERV
DYKCK**KGYFYIPPL**ATHTI**CDRNHTWLP**VSDDACYRETCPYIRDPLNGQAVPANGTYEFG
YQMHFICNEGYYLIGEEILYCELKGSVAIWSGKPPICEKVLCTPPPKIKNGKHTFSEVEV
FEYLDAVTYSCDPAPGPDPFSLIGESTIYCGDNSVWSRAAPECKVVKCRFPVVENGKQIS
GF**GKKFYYKAT**VMFECDKGFYLDGSDTIVCDSNSTWDPPVPKCLKVSTSSTTKSPASSAS
G**PRPTYKPPV**SNYPGYPKPEEGILDSLDVWVIAVIVIAIVVGVAVICVVPYRYLQRRKK**K**
GT**Y**LTDETHREVKFTSL

**From PhosphositePlus (Minimum 5 references)**
**K360, Ubiquitylation [By similarity]**
**Y363, Phosphorylation [By similarity]**

>sp|P15529-3|MCP_HUMAN Isoform D of Membrane cofactor protein OS=Homo sapiens OX=9606 GN=CD46
MEPPGRRECPFPSWRFPGLLLAAMVLLLYSFSDACEEPPTFEAMELIG**KPKPYYEIG**ERV
DYKCK**KGYFYIPPL**ATHTI**CDRNHTWLP**VSDDACYRETCPYIRDPLNGQAVPANGTYEFG
YQMHFICNEGYYLIGEEILYCELKGSVAIWSGKPPICEKVLCTPPPKIKNGKHTFSEVEV
FEYLDAVTYSCDPAPGPDPFSLIGESTIYCGDNSVWSRAAPECKVVKCRFPVVENGKQIS

GF**GKKFYYKAT**VMFECDKGFYLDGSDTIVCDSNSTWDPPVPKCLKVSTSSTTKSPASSAS
G**PRPTYKPPV**SNYPGYPKPEEGILDSLDVWVIAVIVIAIVVGVAVICVVPYRYLQRRKK**K**
GKADGGAEYATYQTKSTTPAEQRG

**From PhosphositePlus (Minimum 5 references)**
**K360, Ubiquitylation [By similarity]**

>sp|P15529-12|MCP_HUMAN Isoform E of Membrane cofactor protein OS=Homo sapiens OX=9606 GN=CD46
MEPPGRRECPFPSWRFPGLLLAAMVLLLYSFSDACEEPPTFEAMELIG**KPKPYYEIG**ERV
DYKCK**KGYFYIPPL**ATHTI**CDRNHTWLP**VSDDACYRETCPYIRDPLNGQAVPANGTYEFG
YQMHFICNEGYYLIGEEILYCELKGSVAIWSG**KPPICEKVL**CTPPPKIKNGKHTFSEVEV
FEYLDAVTYSCDPAPGPDPFSLIGESTIYCGDNSVWSRAAPECKVVKCRFPVVENGKQIS
GF**GKKFYYKAT**VMFECDKGFYLDGSDTIVCDSNSTWDPPVPKCLKG**PRPTYKPPV**SNYPG
YPKPEEGILDSLDVWVIAVIVIAIVVGVAVICVVPYRYLQRRKK**K**GT**Y**LTDETHREVKFT
SL

**From PhosphositePlus (Minimum 5 references)**
**K345, Ubiquitylation [By similarity]**
**Y348, Phosphorylation [By similarity]**

>sp|P15529-4|MCP_HUMAN Isoform F of Membrane cofactor protein OS=Homo sapiens OX=9606 GN=CD46
MEPPGRRECPFPSWRFPGLLLAAMVLLLYSFSDACEEPPTFEAMELIG**KPKPYYEIG**ERV
DYKCK**KGYFYIPPL**ATHTI**CDRNHTWLP**VSDDACYRETCPYIRDPLNGQAVPANGTYEFG
YQMHFICNEGYYLIGEEILYCELKGSVAIWSG**KPPICEKVL**CTPPPKIKNGKHTFSEVEV
FEYLDAVTYSCDPAPGPDPFSLIGESTIYCGDNSVWSRAAPECKVVKCRFPVVENGKQIS
GF**GKKFYYKAT**VMFECDKGFYLDGSDTIVCDSNSTWDPPVPKCLKG**PRPTYKPPV**SNYPG
YPKPEEGILDSLDVWVIAVIVIAIVVGVAVICVVPYRYLQRRKK**K**GKADGGAEYATYQTK
STTPAEQRG

**From PhosphositePlus (Minimum 5 references)**
**K345, Ubiquitylation [By similarity]**

>sp|P15529-13|MCP_HUMAN Isoform G of Membrane cofactor protein OS=Homo sapiens OX=9606 GN=CD46
MEPPGRRECPFPSWRFPGLLLAAMVLLLYSFSDACEEPPTFEAMELIG**KPKPYYEIG**ERV
DYKCK**KGYFYIPPL**ATHTI**CDRNHTWLP**VSDDACYRETCPYIRDPLNGQAVPANGTYEFG
YQMHFICNEGYYLIGEEILYCELKGSVAIWSGKPPICEKVLCTPPPKIKNGKHTFSEVEV
FEYLDAVTYSCDPAPGPDPFSLIGESTIYCGDNSVWSRAAPECKVVKCRFPVVENGKQIS
GF**GKKFYYKAT**VMFECDKGFYLDGSDTIVCDSNSTWDPPVPKCLKVLPPSSTKPPALSHS
VSTSSTTKSPASSASGYPKPEEGILDSLDVWVIAVIVIAIVVGVAVICVVPYRYLQRRKK
**K**GT**Y**LTDETHREVKFTSL

**From PhosphositePlus (Minimum 5 references)**
**K361, Ubiquitylation [By similarity]**
**Y364, Phosphorylation [By similarity]**

>sp|P15529-5|MCP_HUMAN Isoform H of Membrane cofactor protein OS=Homo sapiens OX=9606 GN=CD46
MEPPGRRECPFPSWRFPGLLLAAMVLLLYSFSDACEEPPTFEAMELIG**KPKPYYEIG**ERV
DYKCK**KGYFYIPPL**ATHTI**CDRNHTWLP**VSDDACYRETCPYIRDPLNGQAVPANGTYEFG
YQMHFICNEGYYLIGEEILYCELKGSVAIWSGKPPICEKVLCTPPPKIKNGKHTFSEVEV
FEYLDAVTYSCDPAPGPDPFSLIGESTIYCGDNSVWSRAAPECKVVKCRFPVVENGKQIS
GF**GKKFYYKATV**MFECDKGFYLDGSDTIVCDSNSTWDPPVPKCLKVLPPSSTKPPALSHS
VSTSSTTKSPASSASGYPKPEEGILDSLDVWVIAVIVIAIVVGVAVICVVPYRYLQRRKK

KGKADGGAEYATYQTKSTTPAEQRG

**From PhosphositePlus (Minimum 5 references)**
**K361, Ubiquitylation [By similarity]**

>sp|P15529-14|MCP_HUMAN Isoform I of Membrane cofactor protein OS=Homo sapiens OX=9606 GN=CD46
MEPPGRRECPFPSWRFPGLLLAAMVLLLYSFSDACEEPPTFEAMELIG**KPKPYYEIG**ERV
DYKCK**KGYFYIPPL**ATHTI**CDRNHTWLP**VSDDACYRETCPYIRDPLNGQAVPANGTYEFG
YQMHFICNEGYYLIGEEILYCELKGSVAIWSGKPPICEKVLCTPPPKIKNGKHTFSEVEV
FEYLDAVTYSCDPAPGPDPFSLIGESTIYCGDNSVWSRAAPECKVVKCRFPVVENGKQIS
GF**GKKFYYKAT**VMFECDKGFYLDGSDTIVCDSNSTWDPPVPKCLKVSTSSTTKSPASSAS
GYPKPEEGILDSLDVWVIAVIVIAIVVGVAVICVVPYRYLQRRKKKGTYLTDETHREVKF
TSL

**From PhosphositePlus (Minimum 5 references)**
**K346, Ubiquitylation [By similarity]**
**Y349, Phosphorylation [By similarity]**

>sp|P15529-6|MCP_HUMAN Isoform J of Membrane cofactor protein OS=Homo sapiens OX=9606 GN=CD46
MEPPGRRECPFPSWRFPGLLLAAMVLLLYSFSDACEEPPTFEAMELIG**KPKPYYEIG**ERV
DYKCK**KGYFYIPPL**ATHTI**CDRNHTWLP**VSDDACYRETCPYIRDPLNGQAVPANGTYEFG
YQMHFICNEGYYLIGEEILYCELKGSVAIWSGKPPICEKVLCTPPPKIKNGKHTFSEVEV
FEYLDAVTYSCDPAPGPDPFSLIGESTIYCGDNSVWSRAAPECKVVKCRFPVVENGKQIS
GF**GKKFYYKAT**VMFECDKGFYLDGSDTIVCDSNSTWDPPVPKCLKVSTSSTTKSPASSAS
GYPKPEEGILDSLDVWVIAVIVIAIVVGVAVICVVPYRYLQRRKKKGKADGGAEYATYQT
KSTTPAEQRG

**From PhosphositePlus (Minimum 5 references)**
**K346, Ubiquitylation [By similarity]**

>sp|P15529-15|MCP_HUMAN Isoform K of Membrane cofactor protein OS=Homo sapiens OX=9606 GN=CD46
MEPPGRRECPFPSWRFPGLLLAAMVLLLYSFSDACEEPPTFEAMELIG**KPKPYYEIG**ERV
DYKCK**KGYFYIPPL**ATHTI**CDRNHTWLP**VSDDACYRETCPYIRDPLNGQAVPANGTYEFG
YQMHFICNEGYYLIGEEILYCELKGSVAIWSG**KPPICEKVL**CTPPPKIKNGKHTFSEVEV
FEYLDAVTYSCDPAPGPDPFSLIGESTIYCGDNSVWSRAAPECKVVKCRFPVVENGKQIS
GF**GKKFYYKATV**MFECDKGFYLDGSDTIVCDSNSTWDPPVPK**CLKGYPKPE**EGILDSLDV
WVIAVIVIAIVVGVAVICVVPYRYLQRRKKKGTYLTDETHREVKFTSL

**From PhosphositePlus (Minimum 5 references)**
**K331, Ubiquitylation [By similarity]**
**Y334, Phosphorylation [By similarity]**

>sp|P15529-7|MCP_HUMAN Isoform L of Membrane cofactor protein OS=Homo sapiens OX=9606 GN=CD46
MEPPGRRECPFPSWRFPGLLLAAMVLLLYSFSDACEEPPTFEAMELIG**KPKPYYEIG**ERV
DYKCK**KGYFYIPPL**ATHTI**CDRNHTWLP**VSDDACYRETCPYIRDPLNGQAVPANGTYEFG
YQMHFICNEGYYLIGEEILYCELKGSVAIWSG**KPPICEKVL**CTPPPKIKNGKHTFSEVEV
FEYLDAVTYSCDPAPGPDPFSLIGESTIYCGDNSVWSRAAPECKVVKCRFPVVENGKQIS
GF**GKKFYYKATV**MFECDKGFYLDGSDTIVCDSNSTWDPPVPK**CLKGYPKPE**EGILDSLDV
WVIAVIVIAIVVGVAVICVVPYRYLQRRKKKGKADGGAEYATYQTKSTTPAEQRG

**From PhosphositePlus (Minimum 5 references)**
**K331, Ubiquitylation [By similarity]**

>sp|P15529-9|MCP_HUMAN Isoform N of Membrane cofactor protein OS=Homo sapiens OX=9606 GN=CD46
MEPPGRRECPFPSWRFPGLLLAAMVLLLYSFSDACEEPPTFEAMELIG**KPKPYYEIG**ERV
DYKCKKGYFYIPPLATHTI**CDRNHTWLP**VSDDACYRETCPYIRDPLNGQAVPANGTYEFG
YQMHFICNEGYYLIGEEILYCELKGSVAIWSG**KPPICEKVL**CTPPPKIKNGKHTFSEVEV
FEYLDAVTYSCDPAPGPDPFSLIGESTIYCGDNSVWSRAAPECKVVKCRFPVVENGKQIS
GF**GKKFYYKATV**MFECDKGFYLDGSDTIVCDSNSTWDPPVPKCLKG**PRPTYKPPV**SNYPG
YPKPEEGILDSLDVWVIAVIVIAIGKQMVELNMPLTRLNQPLQQSREAE

>sp|P15529-10|MCP_HUMAN Isoform 2 of Membrane cofactor protein OS=Homo sapiens OX=9606 GN=CD46
MEPPGRRECPFPSWRFPGLLLAAMVLLLYSFSDACEEPPTFEAMELIG**KPKPYYEIG**ERV
DYKCK**KGYFYIPPL**ATHTI**CDRNHTWLP**VSDDACYRETCPYIRDPLNGQAVPANGTYEFG
YQMHFICNEGYYLIGEEILYCELKGSVAIWSGKPPICEKVLCTPPPKIKNGKHTFSEVEV
FEYLDAVTYSCDPAPGPDPFSLIGESTIYCGDNSVWSRAAPECKVVKCRFPVVENGKQIS
GF**GKKFYYKAT**VMFECDKGFYLDGSDTIVCDSNSTWDPPVPKCLKVLPPSSTKP**PALSHS**
**TTK**SPASSASGPRPTYKPPVSNYPGYPKPEEGILDSLDVWVIAVIVIAIVVGVAVICVVP
YRYLQRRKK**K**GT**Y**LTDETHREVKFTSL

**From PhosphositePlus (Minimum 5 references)**
**K370, Ubiquitylation [By similarity]**
**Y373, Phosphorylation [By similarity]**

**3 unique entries/genes**
**16 sequences**
